# Indicator Compounds Representative of Contaminants of Emerging Concern (CECs) Found in the Water Cycle in the United States

**DOI:** 10.3390/ijerph18031288

**Published:** 2021-02-01

**Authors:** Shuangyi Zhang, Stephen Gitungo, John E. Dyksen, Robert F. Raczko, Lisa Axe

**Affiliations:** 1 Shanghai National Engineering Research Center of Urban Water Resources Co., Ltd., Shanghai 200082, China; 2 Department of Civil & Environmental Engineering, New Jersey Institute of Technology, Newark, NJ 07102, USA; swg5@njit.edu; 3 SUEZ North America, Paramus, NJ 07652, USA; consult0851@gmail.com (J.E.D.); bob.raczko@suez.com (R.F.R.); 4 Otto H. York Department of Chemical and Materials Engineering, New Jersey Institute of Technology, Newark, NJ 07102, USA; lisa.b.axe@njit.edu

**Keywords:** contaminants of emerging concern, indicator compounds, water cycle, occurrence and usage, water and wastewater treatment, physicochemical properties

## Abstract

The presence of contaminants of emerging concern (CECs) in the aquatic environment has recently become a global issue. The very large number of CECs reported in the literature makes it difficult to interpret potential risks as well as the removal efficiencies, especially for the more recalcitrant compounds. As such, there is a need for indicator compounds that are representative of CECs detected in systems worldwide. In an effort to develop such a list, five criteria were used to address the potential for applying indicator compounds; these criteria include usage, occurrence, resistance to treatment, persistence, and physicochemical properties that shed light on the potential degradability of a class of compounds. Additional constraints applied included the feasibility of procuring and analyzing compounds. In total, 22 CECs belonging to 13 groups were selected as indicator compounds. These compounds include acetaminophen and ibuprofen (analgesic); erythromycin, sulfamethoxazole, and trimethoprim (antibiotics); diazepam and fluoxetine (antidepressants); carbamazepine (antiepileptic); atenolol and propranolol (β-blockers); gemfibrozil (blood lipid regulator); tris(2-chloroethyl)phosphate (TCEP) (fire retardant); cotinine (nicotine metabolite); atrazine, metolachlor, and N,N-diethyl-meta-toluamide (DEET) (pesticides); 17β-estradiol and cholesterol (steroids); caffeine (psychomotor stimulant); perfluorooctane sulfonate (PFOS) and perfluorooctanoic acid (PFOA) (surfactants); and iopromide (X-ray contrast agent). These thirteen groups of compounds represent CECs with the greatest resistance to treatment processes, most persistent in surface waters, and detected with significant frequency throughout the water cycle. Among the important implications of using indicator compounds are the ability to better understand the efficacy of treatment processes as well as the transport and fate of these compounds in the environment.

## 1. Introduction

The detection of contaminants of emerging concern (CECs) [1] in the water cycle [2,3,4] has raised concern, as they have been found in effluents of wastewater treatment plants, in surface water, and even in drinking water treatment systems. The U.S. Geological Survey (USGS) conducted the first reconnaissance in 2002 that demonstrated the plethora of pharmaceuticals, steroid hormones, and other non-regulated organic compounds in the environment [5]. The presence of these emerging contaminants has led to a number of studies to better understand their fate, transport, and removal. While the consequence of environmental exposure is of the utmost importance, it has received less attention. Nevertheless, studies have demonstrated occurrences in aquatic systems all over the world—for example, in North America [6,7,8,9,10,11,12], in Europe [13,14,15,16,17,18], and in Asia [19,20,21,22,23]. These studies reveal observations of many classes of CECs, with concentrations ranging from nanograms per liter (ng/L) to micrograms per liter (µg/L).

Although CECs are present in the aquatic environment at what may appear to be lower concentrations [19,24,25] than observed for regulated contaminants, the large number of compounds reported in these studies makes it difficult to evaluate the impact as they involve compounds that are widely used, frequently detected, recalcitrant, as well as present as mixtures in the water cycle. Their occurrence in the effluents from the wastewater treatment plants (WWTPs) and the transport into source water for drinking water treatment facilities has led to the need to better understand the efficacy of treatment processes in degradation, transformation, and removal. Similarly, the large number of compounds makes the cost of analysis significant. Furthermore, once released, these products pose a potential threat to aquatic organisms, water resources, and human health [26,27,28]; studies have demonstrated, for example, that CEC exposure resulted in antibiotic resistance and intersex in fish, and hyperglycemia and hepatic histological abnormalities in mice [29,30,31]. Human health risks have also been evaluated in a number of studies [32,33,34,35,36,37]. Given the vast number of these compounds, using indicator compounds that are representative of CECs in water systems would be highly advantageous in tracking their behavior in the environment and in evaluating their removal through pilot plant studies and full-scale treatment plants; a further benefit is the potential for reducing the extent and cost of analysis.

The objective of this paper is to demonstrate that based on studies conducted to date, indicator compounds can be developed based on the vast array of CECs that are detected throughout the water cycle in the United States and recalcitrant to the treatment processes. These compounds are ones that can then be applied in further studies to address transport and fate in the environment as well as treatment efficacy in water and wastewater plants. The rationale for selecting criteria will be reviewed. Based on the criteria, indicator compounds are delineated with the expectation that they are representative of wide-scale use and occurrence, resistant to treatment, persistent in the environment, and the range of physiochemical properties; again, these compounds would aid in tracking CECs and would reduce the number of CECs to be analyzed.

## 2. Criteria for Selecting Indicator Compounds

**Figure 1:**
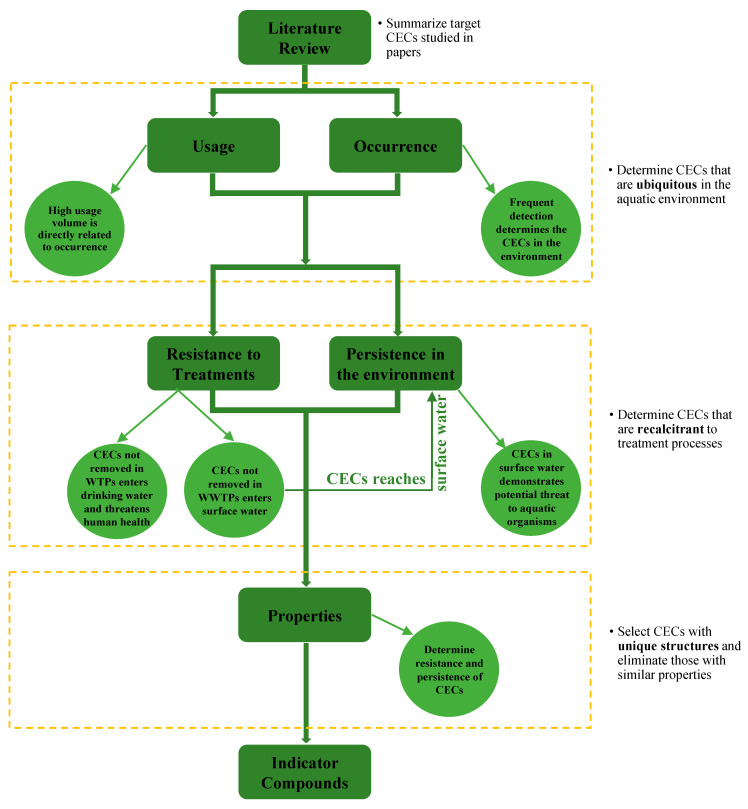
Criteria and flow for identifying indicator compounds.

Based on an exhaustive literature review, criteria that reflect issues related to CEC observances in the water cycle include usage, frequency of detection, resistance to treatment, persistence, and physicochemical properties (Figure 1). Usage of CECs directly corresponds to the probability of occurrence in the environment. Occurrence of CECs in the aquatic environment poses a potential risk to human health and the associated ecosystems. Resistance to treatment processes characterizes whether a CEC is removed through WWTPs and discharged to the surface water that may serve as the source water for water treatment plants (WTPs), while CECs that go untreated in WTPs reflect their potential presence in drinking water, which may pose potential human health risks. Persistence demonstrates the stability of the CECs as well as the resistance to degradation in the environment, which may pose potential risks to aquatic organisms. Some CECs that are recalcitrant through treatments and persistent in the environment may not be widely used or frequently studied but are always detected when analyzed; these compounds cannot be neglected and need to be considered. Physicochemical properties such as structure, K_ow_, K_oc_, k_O3_, and solubility determine the behavior of the CECs during treatment processes and in the environment and are therefore related to their fate during environment transport. In the following sections, these five criteria are examined with respect to CECs and the development of indicator compounds.

## 3. Usage and Occurrence of CECs in the United States

### 3.1. Usage of CECs

CECs refer, in general, to any product used by individuals for personal health or cosmetic reasons or used in the agricultural industry to enhance plant or animal growth and health. CECs comprise a diverse collection of thousands of chemical substances, including prescription and over-the-counter therapeutic medications, veterinary drugs, fragrances, cosmetics, and pesticides. Because of the significant use and sources of CECs, they have been present in water and the environment for as long as humans have been using them [38]. CECs enter WWTPs through human excrement [39], inappropriate disposal into sewage systems such as flushing unused medications in toilets (and other drains), runoff and infiltration from agriculture [40,41], as well as disposal from hospitals [42,43,44,45]. Because of the wide use of CECs and resulting release into the water cycle, they have been observed over the last decade worldwide and pose a potential threat to the environment. The occurrence of CECs in WWTPs and the environment is greatly dependent on their usage, which can be reflected by sales and dispensed prescriptions.

Drug sales in the U.S. grew annually from 2006, except in 2012. For example, the total spending increased by 13.1% to USD 373.9 billion in 2014 compared to 2013 and went up by 12.2% from 2014 to 2015 [46,47]. The number of prescriptions dispensed from retail channels grew by roughly 1.0% from 2014 to 2015 and 2.1% between 2013 and 2014. A total of 4.4 billion prescriptions were dispensed in 2015. Clearly, the large usage of pharmaceuticals has a potentially significant impact when excreted and not removed in WWTPs.

Among the top therapeutic classes by U.S. sales and dispensed prescriptions, lipid regulators, antidepressants, analgesics, β-blockers, antiepileptics, and antibiotics are of concern as they are the most frequently detected pharmaceutical classes in aqueous systems. Antidepressants were the most dispensed prescription in 2011 (264 prescriptions) (Table S1), attaining the seventh highest number of sales in that year (Table S2). Lipid regulators were ranked second in prescriptions dispensed, growing annually from 233 million in 2007 to 260 million in both 2010 and 2011, and ranking third in total sales. The usage of analgesics increased annually from 2007, totaling 244 million prescriptions in 2010 and 238 million in 2011, ranked third among all therapeutic classes and 10th based on sales. Beta-blockers are also widely used, ranking sixth, with 161 million prescriptions in 2011. Antiepileptics were 10th among the top therapeutic classes dispensed, totaling 128 million prescriptions in 2011, with its sales ranked 16th due to the high demand. The usage of antibiotics ranked 18th and totaled 69 million prescriptions in 2010. Not surprisingly, among pharmaceuticals, the following drugs are the most widely prescribed: lipid regulators, antidepressants, analgesics, β-blockers, antiepileptics, and antibiotics.

Contrast media agents represent a category of CECs that are widely used in hospitals. North America shared approximately 46% of the global market of contrast media agents in 2016 [48]. Among the sales of all types of contrast media in the United States, iodinated contrast media agents (ICMs) shared nearly 70% sales in the market, registering the greatest market size. Due to their wide use, ICMs have been detected in WWTP effluents, source water, and drinking water [49,50,51].

In addition to medical-related CECs, pesticides are another category of CECs that are frequently used and therefore found in aquatic environments [52]. In the U.S., pesticides applied totaled approximately 1.1 billion pounds in both 2006 and 2007 [53]. Atrazine has been ranked first in agriculture use for a number of years (1987 to 1999) [54] (Table S3). As a result, among the most widely used pesticides, atrazine and metolachlor were most frequently detected in the water cycle [5,49,51,55,56,57,58] and strongly related to agricultural land use [52]. Agriculture contributes 70% to 83% of pesticide usage in the U.S. [53] and expected runoff from the agricultural areas has been observed as the predominant source of pesticides in surface waters [59,60,61,62]. Usage of CECs is an indicator of estimated release into the environment. Usage is directly related to occurrence in aquatic environments.

### 3.2. Occurrence of CECs in Wastewater

**Figure 2:**
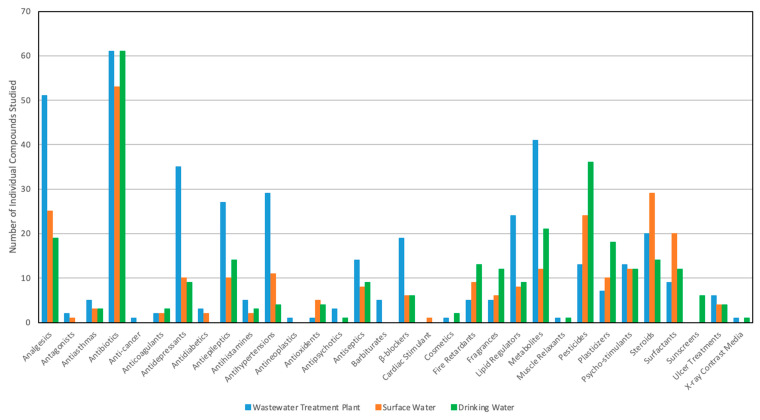
Number of individual compounds studied in each CEC class (data from Tables S5–S7).

Studies have been conducted on the occurrence of CECs in wastewater across the U.S. (Figures S1–S14) [6,9,10,12,49,56,63,64,65,66,67,68,69,70,71,72,73,74,75,76,77,78,79]. The most frequently reported classes include analgesics, antibiotics, antidepressants, antiepileptics, antihypertensions, antiseptics, β-blockers, lipid regulators, CEC metabolites, pesticides, psycho-stimulants, and steroids (Figure 2 and Figure S1). Acetaminophen, diclofenac, ibuprofen, and naproxen were the most detected analgesics (each reported in more than eight studies), with acetaminophen found in all samples collected from WWTP influents (Figures S1 and S2). The greatest concentrations were observed for acetaminophen, ibuprofen, naproxen, and salicylic acid, ranging from 370 to 218,000 ng/L; these concentrations are one to three orders of magnitude greater than other investigated analgesics (i.e., brompheniramine, codeine, diclofenac, indomethacin, and mefenamic acid) (Figure S2). The ubiquity and influent concentrations of analgesics observed are consistent with prescriptions dispensed and the overall sales (Tables S1 and S2). Usage is a predictor of occurrence in wastewater influents, as up to 95% of pharmaceuticals taken may be excreted [39,44,80]. Lienert et al. (2007) studied 42 pharmaceuticals from 22 therapeutic classes and found that as much as 88% of the parent dug was excreted via urine and as much as 90% via feces [39]. For the metabolites, 0–100% was excreted via urine and 0–34% via feces [39]. Specifically, the percent excretion on average for acetaminophen is 83%, 16% to 51% for diclofenac, and 5% to 100% for ibuprofen [39,80,81] (Table S4). These results confirm that a significant percentage of pharmaceuticals used today are excreted through urine and feces, which enter the municipal wastewater streams.

Similarly, antibiotics are the most frequently studied class, with sulfamethoxazole reported in 11 studies and trimethoprim found in seven studies (Figures S1 and S4). The influent concentrations range from 14 to 3905 ng/L for sulfamethoxazole and from 220 to 1140 ng/L for trimethoprim; these peak concentrations are approximately ten times greater than other antibiotics (Figures S1 and S4). With the total excretion as great as 98% for sulfamethoxazole [39] and 40–69% for trimethoprim [81] (Table S4), the average detection frequency in WWTP influent was 100% for both compounds (Figure S1 and Table S5).

Another frequently detected CEC class is antidepressants. Eight out of 13 antidepressants have been detected in 100% of WWTP influents sampled (concentrations range from 2.3 to 609 ng/L) (Figure S5), which is not only consistent with their usage across the U.S. (the most prescribed therapeutic class and the seventh based on sales) (Tables S1 and S2), but also relates to their excretion. For example, the most investigated antidepressants, diazepam (reported in four studies) and fluoxetine (reported in six studies), are excreted on average at 85% and 71%, respectively, including the excretion of the parent drug and metabolites [39] (Table S4). The occurrence of the CECs in the influents to WWTPs is strongly related to their usage and total excretion. Other CECs that were detected in 100% of the influent samples include carbamazepine (antiepileptic), diltiazem (antihypertension), atenolol and propranolol (β-blockers), gemfibrozil (lipid regulator), and caffeine (psycho-stimulant) (Figure S1), which are widely used (Tables S1 and S2) with high excretion (Table S4).

### 3.3. Occurrence of CECs in Surface Water

With incomplete removal of CECs in the WWTPs, wastewater discharges are the main source of their occurrence in surface water [58,63,82,83,84,85]. Bartelt-Hunt et al. (2009) compared the concentrations of 19 pharmaceuticals in the surface water that were sampled upstream and downstream of the WWTP discharges [63]. Acetaminophen, azithromycin, carbamazepine, sulfadimethoxine, sulfamethazine, sulfamethoxazole, and thiabendazole that were not detected in the upstream samples were found at concentrations ranging from 153.2 to 4679 ng/L in the receiving surface water. Concentrations of caffeine, DEET, and diphenhydramine were observed to be two orders of magnitude greater in the downstream samples. Significantly greater CEC concentrations in the downstream samples than those observed in the upstream samples demonstrate that WWTP effluents are a significant source for the downstream surface waters. As also observed in a number of studies, the most reported groups of CECs in effluent samples reveal influents to WWTP as the source (Figure S1). With incomplete removal through existing WWTP treatment processes and the release to surface water, CECs will not only be detected but may also be persistent in the environment; however, this will depend on the compound’s physicochemical properties (Figure S15).

Occurrence of CECs in the water cycle is impacted by runoff from agriculture as well [52,81]. Kim and Carlson (2006) examined the occurrence of three veterinary antibiotics (i.e., monensin, salinomycin, and narasin) in samples collected from the surface water close to an agricultural area during low-runoff (monthly mean stream flow 3.5 to 9.2 cm) and high-runoff (monthly mean stream flow 0.2 to 2.3 cm) conditions [86]. Concentrations ranged from below detection to 13 ng/L during the low-runoff period and increased to up to 36 ng/L during the high-runoff period. After a rain event with accumulated precipitation exceeding 24 mm in 48 h, Matamoros et al. (2012) observed an increase in herbicide/pesticide concentrations in a river receiving runoff from the agricultural fields [87]. For example, the concentration of mecoprop increased from less than 200 ng/L to approximately 1500 ng/L. As a result, herbicides, pesticides, and veterinary antibiotics enter the water cycle through agricultural practices.

Seasonal variation in the occurrence of CECs in surface water also needs to be considered. Compared to the winter season (dry season), CEC concentrations are lower in summer (wet season) by more than 50%, which is attributed to dilution from increased precipitation with higher temperatures [57]. Elevated temperatures may result in transformation of CECs [88]. Padhye et al. (2014) studied the impact of precipitation and dilution on a surface water serving as a source for potable water utilities and potentially not affected by anthropogenic activities [57]. Despite the reduced concentrations observed in the summer months, 20 CECs were at least detected once in the surface water while atrazine, DEET, carbamazepine, clarithromycin, erythromycin, sulfamethoxzole, trimethoprim, metoprolol, nonyphenol, and TCEP were detected in 88% of the samples, reflecting their persistence. It is clear that population density in the vicinity of surface water is considered to be another impact factor in the occurrence of CECs [89]. Other studies [52,90] have demonstrated that concentrations of CECs in water bodies adjacent to heavily populated areas were observed to be two to 10 times greater than those observed in pristine areas.

In summary, a number of studies have demonstrated the occurrence of CECs in surface water [5,57,58,63,82,83,84,85,86,88,89,90,91,92,93,94,95,96,97,98,99,100,101,102] (Figures S15–S22). The most reported classes include analgesics, antibiotics, antidepressants, antiepileptics, antihypertensions, metabolites, pesticides, plasticizers, psycho-stimulants, steroids, and surfactants (Figure 2 and Table S6). Acetaminophen, ibuprofen, and naproxen were the most frequently studied analgesics, with average detection frequency ranging from 18% to 33% (Figure S15). Of antibiotics, sulfamethoxazole and trimethoprim were most frequently detected (Figure S15), while the sulfamethoxazole concentrations were one to four orders of magnitude greater than the other antibiotics (Figure S17). Their occurrence in surface water was strongly correlated to the concentrations in WWTP effluents (Figures S4 and S17). Carbamazepine is the most reported antiepileptic, with a detection frequency of 69% (Figures S1 and S15), suggesting it is not only resistant to treatment processes but also possibly persistent in the environment. Recently, per- and polyfluoroalkyl substances (PFAS) have gained more attention for their persistence in surface water. Among the large number of PFAS, PFOA and PFOS are the two most reported and researched substances [95]; their concentration is as great as approximately 10^9^ ng/L in the surface water near a carpet industry area in Georgia [97]. The average detection frequencies across the U.S. surface water are 94% and 64% for PFOA and PFOS, respectively (Figures S15 and S22), suggesting their persistence in the environment, which may result from historic release, ongoing discharge, or the lack of degradation [95]. The antimicrobial triclosan, fire retardant TCEP, pesticide DEET, and psycho-stimulant caffeine are the most studied compounds in their classes and were detected at similar frequencies in samples collected from WWTP effluents (62.5% to 85%) and surface water (53% to 80%) (Figures S1 and S15). Other frequently studied CECs include the antidepressant fluoxetine, the blood lipid regulator gemfibrozil, the nicotine metabolite cotinine, the plasticizer bisphenol-A, and the steroid estrone. The occurrence of CECs in surface water indicates their persistence and presence in source water for drinking water treatment plants, which then raises the issue of potential adverse effects on human health from individual as well as mixtures of CECs.

### 3.4. Occurrence of CECs in Source Water and Finished Drinking Water

The occurrence of CECs in drinking water is of the utmost importance as it leads to direct exposure in human beings. Similar to surface water, the occurrence of CECs in the source water for WTPs is affected by upstream discharges from WWTPs [50,84,103,104] as well runoff from agriculture [51]. On the other hand, the occurrence of CECs in finished drinking water is dependent on the removal efficacy in WTPs, namely their resistance to treatment [57,104]. A number of studies have evaluated occurrence in source water and finished drinking water [50,51,55,57,84,103,104,105,106,107]. The most frequently observed classes in these systems included analgesics, antibiotics, antiepileptics, fire retardants, fragrances, CEC metabolites, pesticides, plasticizers, psycho-stimulants, steroids, and surfactants (Figure 2 and Table S7). Among 131 investigated CECs (Table S7), 22 were reported in more than four studies (Figures S23–S32) [50,51,55,57,84,95,98,103,104,105,106,107,108,109,110].

Compared to the wastewater and surface water, much lower detection frequencies have been reported in source water and finished drinking water (Figure S23). For example, of 29 antibiotics investigated in drinking water [51,57,103,104], only nine were detected in source water samples and four (clarithromycin, erythromycin, sulfamethoxazole, and trimethoprim) in finished drinking water (Figure S25). Similar trends were observed for fragrances, lipid regulators, and steroids, where less than 25% of the investigated compounds were detected in both source water and drinking water samples.

For CECs frequently reported in sampled source water, concentrations have been generally less than 1000 ng/L; exceptions included acetaminophen, ibuprofen, carbamazepine, and bisphenol-A, with the maximum concentration observed greater than 1500 ng/L (Figure S23). Compared to the source water samples, concentrations in finished drinking water are generally less than 100 ng/L, with the exception of ibuprofen, carbamazepine, triclosan, TCEP, nonylphenol, metolachlor, bisphenol-A, caffeine, and PFOA (Figure S23). The lower concentrations reported in the sampled finished drinking water compared to the source water reveal some degree of removal in WTPs. Nevertheless, CECs are frequently detected in finished drinking water for a number of classes, including the antibiotics, antidepressants, antiepileptics, fire retardants, fragrances, the nicotine metabolite cotinine, pesticides, the psycho-stimulant caffeine, and surfactants (Figure S23). As a result, these compounds may be resistant to conventional treatment processes in WTPs and their removal efficiencies need to be evaluated.

Furthermore, the high frequency of detection (Tables S6 and S7) demonstrates persistence in aquatic environments. For example, the steroid cholesterol was only investigated in two studies; however, its average frequency of detection in surface water samples was 90%, suggesting little to no degradation in the systems (Figure S22). Similarly, for the antibiotic erythromycin and the β-blocker metoprolol, the average frequencies are 59% and 100%, respectively, in surface water samples (Figures S17 and S19), while the detection frequency of surfactants PFOA and PFOS was more than 50% in the source water and the steroid estrone was detected in 77% of source waters sampled (Figures S31 and S32). Persistence in the aquatic environment therefore is an important criterion for determining what CECs make their way to the tap and are candidate indicator compounds.

## 4. Efficacy of Treatment Technologies

### 4.1. Removal Efficacy in WWTPs

As WWTPs are not designed to treat CECs, their removals are limited. For example, removals through primary clarification are reported to be less than 36% [69,75,111,112], while no reductions were reported for diclofenac, trimethoprim, carbamazepine, diltazem, propranolol, and caffeine in this unit process (Table 1a) [69,79].

Secondary biological treatment processes such as conventional activated sludge, trickling filters, and moving bed biofilm reactors (MBBRs) address the biochemical oxygen demand, chemical oxygen demand, nitrogen, and phosphorus [63,72] and are not efficient in treating CECs. For example, Bartelt-Hunt et al. (2009) investigated the effluent concentration of sulfamethoxazole in WWTPs that employed trickling filters [63]. An average effluent concentration of 141.4 ± 22.3 was observed. Other studies [69,111,113,114] have reported removals of less than 69% for sulfamethoxazole and less than 5% for trimethoprim using activated sludge, while their removals in MBBRs ranged from 0% to 36% (Table 1a). Because of the low removal efficacies, average detection frequencies for the most reported antibiotics, sulfamethoxazole and trimethoprim, were 88% and 43%, respectively. The most reported antidepressants are diazepam and fluoxetine, with detection frequencies of 71% and 38% in the effluent samples, respectively. This occurrence is consistent with the relatively low removal efficacies of 41% to 55% for diazepam and 72% for fluoxetine (Table 1b). Carbamazepine is the most studied antiepileptic, with a detection frequency of 100% in WWTP influent samples and 90% in sampled effluents (Figures S1 and S6). A number of studies have reported that carbamazepine is a recalcitrant compound for existing WWTPs, with removals of less than 10% through activated sludge and less than 11% through MBBRs [75,113,115,116] (Table 1a). Other recalcitrant CECs in the WWTPs include the antihypertension diltiazem and β-blockers atenolol and propranolol, with total removal efficacies of less than 50% in the WWTPs (Table 1b). The advanced treatment processes such as activated carbon and membrane technologies may improve their removals; however, these processes may not be sufficiently effective to remove the vast array of CECs from the wastewater to desired levels. For example, Khanzada et al. (2020) reported CEC removals ranging from poor (<20%) to fair (40–70%) in ultrafiltration and good (70–90%) to excellent (>90%) in activated carbon and nanofiltration [117]. The low removals result in their presence in effluents and surface water.

Studies to date have demonstrated that wide-scale usage results in occurrence throughout the water cycle, which then impacts aquatic ecosystems (Figure 1). The vast array of the recalcitrant CECs and their persistence in the environment pose potentially adverse effects through exposure to the individual compounds as well as the extensive mixtures.

### 4.2. Adverse Effects from CECs

CECs are making their way to surface water and therefore exert potentially adverse effects on aquatic ecosystems. Although concentrations in the ng/L may appear to be relatively low, environmental exposure cannot be ignored. For example, in a wetland receiving secondary-treated wastewater effluents, the accumulation of insecticide dieldrin was observed in Mosquitofish (*Gambusia affinis*) at 8.3 ng/g of the whole fish body [123]. Brozinski et al. (2013) detected diclofenac (6–148 μg/L), naproxen (6–103 μg/L), and ibuprofen (15–34 μg/L) in the bile of two wild fish bream (*Abramis brama*) and roach (*Rutilus rutilus*) living in a lake that receives discharges with CECs from WWTPs [124]. CECs have been found to alter the freshwater macroinvertebrate community structure [125]. For example, the discharge of carbamazepine (1 to 88 ng/L) from WWTP effluents and septic wastewater impacted the abundance of Baetidae in surface water downstream [125], where the Baetidae abundance (r = 0.52, *p* = 0.022) and macroinvertebrate richness (r = 0.48, *p* = 0.037) significantly increased.

Observations of antibiotic resistance of bacteria [30,126,127,128,129,130], intersex occurrence in freshwater fish [31,131,132,133], and oxidative stress in aquatic organisms [134,135,136,137] are major adverse effects from CECs in the water cycle [138]. Many researchers have investigated the effect of the wastewater treatment processes on the prevalence of antibiotic-resistant bacteria in the plants and receiving waters [126,128,129]. A number of stream surveys documented the significant prevalence of native bacteria that display resistance to a wide array of antibiotics, including vancomycin [139]. Lateef (2004) examined 25 bacterial strains isolated from a pharmaceutical company’s effluent and their resistance to commonly used antibiotics [127]. Around 80% of the isolates were resistant to amoxicillin, 76% to nitrofurantoin, 64% to cotrimoxazole, and 12% to gentamicin. These strains impact ecosystems, animal husbandry, and humans. WWTPs are important reservoirs of bacteria in which antibiotic-resistant organisms persist and may be released into the environment. As environmental compartments are interconnected, including municipal sewage and surface water, WWTPs may facilitate the spread of antibiotics, antibiotic resistance genes, and antibiotic-resistant bacteria [140]. Zhang et al. (2009) found that the frequency of antibiotic-resistant bacteria to rifampin, chloramphenicol, and amoxicillin/clavulanic acid was greater downstream than upstream in a river receiving a wastewater effluent discharge [140]. Such effluents contribute to the increase in antibiotic resistance in aquatic environments. These reports suggest that the occurrence of antibiotic-resistant bacteria is much greater than expected where the uncontrolled release of antibiotics into the environment may prompt an increase in resistance. Excluding the significance of antibiotics themselves in the environment, their occurrence implicates the presence of other CECs and suggests further adverse effects on humans, including increased antibiotic resistance, resulting in the formation of superbugs.

During the last decade, a significant amount of research has helped to clarify the potential risk of exposure to endocrine-disrupting chemicals (EDCs) [131,132,141,142,143,144,145]. Adverse effects on fish populations have been frequently recorded downstream of sources of aquatic contamination. Masculinization of female fish was one of the first recorded effects, when mosquito fish downstream of a pulp and paper mill were found to have male secondary sexual characteristics [146]. The converse effect has been frequently reported as well in freshwaters downstream of wastewater treatment plants, where feminization of male fish and mollusks through estrogen contamination in the effluent has a pronounced effect. Feminization of reproductive ducts in male fish, appearance of oocytes in male gonads, and the characteristic production of the female egg protein vitellin in male fish exposed to wastewater from sewage treatment plants have been recorded [147]. Feminization of fish in English rivers is widespread, attributed to estrogen in sewage effluent [143]. Jobling et al. were the first to document an example of widespread sexual disruption in wild populations of any vertebrate, demonstrating that reproductive and developmental effects do result from exposure to ambient levels of chemicals present in rivers. A 7-year lake experiment was conducted in Northwestern Ontario, Canada [132], for determining the adverse impact of chronic exposure to a complex mixture of compounds containing estrogens and estrogen-mimicking compounds on fish populations. The results showed that chronic exposure of fathead minnows to low concentrations (5–6 ng/L) of 17-α-ethynylestradiol led to feminization of males. Impacts on gonadal development as evidenced by intersex in males and altered oogenesis in females, and, ultimately, the near extinction of this species from the lake, demonstrated that what are considered low concentrations of estrogen and estrogen mimicking compounds may have profound developmental effects on wild fish populations. Hinck et al. (2009) found that intersex occurred in 3% of freshwater fish evaluated in nine river basins in the U.S. [131]. Intersex was most prevalent in largemouth bass (8–91% per site) and small mouth bass (14–73% per site). The authors hypothesized that the prevalence of intersex may be related to the season, the age of fish, and the endocrine active compounds in the environment.

A number of studies have focused on the oxidative stress induced by the exposure to environmentally relevant concentrations of CECs. Almedia et al. (2015) evaluated the chronic toxicity of carbamazepine on clams (*Ruditapes philippinarum*) exposed to 0.03, 0.30, 3.00, and 9.00 μg/L for 28 days [134]. At greater concentrations of 3.00 and 9.00 μg/L, a significant decrease in the antioxidant enzymes was observed and led to the impairment of the ability to cope with oxidative stress. Similarly, in galaxiid fish, an acute exposure to 0.17 μg/L of diclofenac for 96 h resulted in a decrease in catalase activity in the gill tissue [135]. Stancova et al. (2017) evaluated the early stages of oxidative stress in Tench fish exposed to environmentally relevant concentrations (0.02–60 μg/L) of ibuprofen, diclofenac, and carbamazepine [137]. Significant impacts were observed in oxidative stress parameters glutathione reductase, glutathione peroxidase, and glutathione-S-transferase after the exposure. These results suggest that CECs, even at low concentrations ranging from ng/L to µg/L, exert potentially adverse effects on aquatic organisms. Therefore, CECs that make their way through WTPs and persist in the drinking water may threaten human health and are most important.

### 4.3. Removal Efficacy in WTPs

A significant amount of work has been focused on treatment through WTPs. Studies have included evaluating the effectiveness of conventional processes that include coagulation, filtration, and disinfection along with advanced processes such as granular activated carbon (GAC), biologically active filters (BAFs), ozonation with and without H_2_O_2_, and UV/H_2_O_2_. A number of studies have been conducted to determine the degree to which individual and combined conventional and advanced water treatment processes are capable of removing CECs.

Coagulation has been reported as ineffective in treating CECs [16,104,148,149,150,151]. Westerhoff et al. (2005) evaluated the removal of CECs in coagulation with aluminum sulfate and ferric chloride [151]. Generally, at a pH of 6.8, removals were less than 50%. The exception included a few polycyclic aromatic hydrocarbons (e.g., benzo[k]fluoranthene, and benzo[a]pyrene) and the pesticide dichlorodiphenyl-dichloroethylene (DDE), with log K_ow_ values greater than 5.5 indicating their hydrophobic properties and potential for partitioning during coagulation. Similar trends were observed by Huerta-Fontela et al. (2011), where removals were less than 30% for hydrophilic compounds (log K_ow_ < 3) such as atenolol, propranolol, carbamazepine, and diltiazem [16]. Overall, coagulation is a relatively ineffective process. Similarly, dual media filtration yields poor removals as well. In a pilot plant study, anthracite/sand dual media filters with empty bed contact time (EBCT) of 6 min achieved removals of less than 50% for the 15 CECs studied [152] (Table 2). Chlorine was found to be an ineffective oxidizing reagent for CECs. Studies examined the removal efficiency of chlorination, with dosages ranging from 1 to 2.6 mg/L and a contact time of 60 min [152,153,154]. Limited removals (0% to 62%) were observed (Table 2). For example, with a dosage of 1 mg Cl_2_/L, removals of less than 40% were reported for the antiseptic triclosan (36%), analgesic ibuprofen (3%), steroid estrone (36%), lipid regulator clofibric acid (5%), and X-ray contrast agent iopromide (0%) [154]. Only steroids including estriol (53%), estradiol (58%), and ethynlestradiol (41%) were removed by greater than 40%. Overall, conventional treatment processes including coagulation, filtration, and chlorination showed limited CEC removal.

Virgin GAC was highly effective in treating CECs, with removals generally greater than 90%; the exception was DEET, with a removal of approximately 70% [52]. However, consideration of breakthrough and regeneration frequency needs to be addressed for GAC [155,156]. PFOA and PFOS were removed to below their detection limits in a full-scale GAC contactor with an EBCT of 13 min [148]. After a bed volume of approximately 65,000, the removal dropped to less than 50% and 75% for PFOA and PFOS, respectively. CECs were removed by greater than 90% in a full-scale GAC with an EBCT of 7.6 min. However, 20% breakthrough was observed after bed volumes ranging from 30,700 for sulfamethoxazole to 78,600 for carbamazepine [155]. K_oc_ is an important indicator of the GAC’s effectiveness; less than 570 mg/g C resulted in removals of less than 40% [52] (Table 3). For TCEP (K_oc_ = 71 mg/g C) and DEET (K_oc_ = 300 mg/g C), removals were less than 43.2% in a full-scale GAC filter with an EBCT ranging from 1.5 to 3 min [104]. Amitrol, nonylphenol, and bisphenol-A, with log K_ow_ of −0.86, 5.76, and 3.32, respectively, were studied, and while the latter two were effectively adsorbed, amitrol was not [157].

Biologically active filters (BAF) are gaining more attention in treating CECs. Recently, a number of studies have been conducted using BAFs to treat CECs in drinking water [160,161,162,163,164,165]. BAF efficiency was observed to be a function of EBCT [160,161,162]. For example, in a bench-scale study with an anthracite/sand BAF as the EBCT increased from 5 to 14 min, the removal of the pesticide DEET improved from less than 50% to as high as 70%, while ibuprofen removal increased from 70% to greater than 90%. Zearley and Summers (2012) observed improvements for acetaminophen, naproxen, caffeine, and gemfibrozil when the EBCT doubled from 7.9 to 15.8 min [161]. Pre-ozonation has been found to increase the biodegradable fraction of the natural organic matter (NOM) [163]. Lee et al. (2012) examined CEC removal as a function of pre-ozonation dosage in anthracite BAF [163]. Nine out of 15 CECs were removed at greater than 75% with an ozone dosage of 4 mg/L, while only three compounds were removed to the same extent when the dosage decreased to 2 mg/L. Zhang et al. (2017) converted existing filters in WTPs into BAFs and evaluated the CEC removals as a function of media, EBCT, and pre-ozonation [165]. At an 18-min EBCT, GAC BAFs without ozonation removed CECs studied at greater than 80%; the exceptions included TCEP and iopromide, which were removed at 76% and 59%, respectively. Reducing the EBCT to 10 min resulted in less than half of the CECs being removed to the same extent. Dual-media BAFs showed limited removals, with only four compounds removed at greater than 80% at either EBCT.

Ozonation degrades the contaminants by direct oxidation and hydroxyl radical (OH•). Generally, increased removals were observed with increasing ozone dosage. Wert et al. (2009) investigated three ozone dosages, 0.2, 0.6, and 1.0 mg O_3_/mg total organic carbon (TOC), in a pilot plant study where tertiary-treated effluents from three WWTPs with TOC ranging from 6.6 to 10.3 mg/L were tested [159]. Removals of 23 CECs through ozonation were evaluated. The most recalcitrant compounds were atrazine, iopromide, tris(1-chloro-2-propyl) phosphate (TCPP), and TCEP, with removals less than 60% even at the greatest ozone dosage 9.27 ± 2.31 mg/L. Thirteen compounds were removed at greater than 80%, with two of the ozone dosages of 5.87 ± 1.63 and 9.27 ± 2.31 mg O_3_/L, while all 23 compounds were removed at less than 80% for the lowest ozone dosage of 2.27 ± 0.49 mg O_3_/L. As a selective oxidant, ozone rate constants (k_O3_) vary between less than 1 and 3 × 10^6^ M^−1^ s^−1^. Compounds with k_O3_ greater than 10^5^ M^−1^ s^−1^ are highly reactive with ozone [166]. In a pilot plant study, with an ozone dosage of 0.3 mg O_3_/mg TOC, acetaminophen (k_O3_ = 2.1 × 10^5^ M^−1^ s^−1^), sulfamethoxazole (k_O3_ = 5.5 × 10^5^ M^−1^ s^−1^), trimethoprim (k_O3_ = 2.7 × 10^5^ M^−1^ s^−1^), carbamazepine (k_O3_ = 3 × 10^5^ M^−1^ s^−1^), and 17β-estradiol (k_O3_ = 10^6^ M^−1^ s^−1^) were removed at greater than 90%, while average removals were less than 60% for compounds with k_O3_ less than 10 M^−1^ s^−1^, such as atrazine, DEET, ibuprofen, TCEP, and iopromide [152].

Compared to ozone, OH• rate constants differ by less than one order of magnitude, with less selectivity than O_3_ [151,159,167]. Gerrity et al. (2011) evaluated the removals in ozonation with the addition of H_2_O_2_ at a mass ratio of 0.7 [49]. Removals were considerably improved, with almost all the compounds removed at greater than 80%; the exceptions were atrazine, TCEP, and TCPP. The recalcitrant compounds for ozonation included pesticide atrazine, fire retardants TCEP and TCPP, and the X-ray contrast agent iopromide.

Removal efficiency in advanced oxidation process UV/H_2_O_2_ is dependent on the UV intensity and H_2_O_2_ addition. At the typical UV dosage used in disinfection of 40 mJ/cm^2^ [168], CECs removals were poor (<20% for target compounds). Increasing the dosage by an order of magnitude resulted in increased removal of the majority of target compounds, with only four compounds (i.e., TCEP, DEET, metolachlor, and caffeine) removed at less than 20%. UV with H_2_O_2_ provides improved performance as UV activates H_2_O_2_ and leads to the formation of the OH• radical. Rosario-Ortiz et al. (2010) investigated the removal of six CECs (atenolol, carbamazepine, dilantin, meprobamate, primidone, and trimethoprim) as a function of UV intensity (300, 500, and 700 mJ/cm^2^) with a range of H_2_O_2_ dosages (0 to 20 mg/L) [169]. At the lowest UV intensity, removals were generally less than 50%. With UV intensity greater or equal to 500 mJ/cm^2^ and H_2_O_2_ dosages between 15 and 20 mg/L, removals of as great as 90% were observed for atenolol, carbamazepine, and trimethoprim. Generally, UV oxidation alone with an intensity of 500 mJ/cm^2^ or greater is a promising process for treating CECs [169]. With intensity of less than 500 mJ/cm^2^, the addition of H_2_O_2_ is required.

In a pilot plant study, 12 treatment trains were investigated to understand the optimal combination of unit processes for treating 15 indicator CECs. Unit processes included dissolved air flotation (DAF), pre- and intermediate-ozonation with and without H_2_O_2_, intermediate chlorination, dual-media filtration, GAC, and UV/H_2_O_2_ [152]. The most effective trains involved 1. DAF followed by intermediate ozonation, dual-media filtration, and virgin GAC; 2. pre-ozonation followed by DAF, dual-media filtration, and virgin GAC; and 3. DAF (with either pre- or intermediate oxidation) followed by dual-media filtration and UV/H_2_O_2_, where 13 to 15 of the indicator compounds were removed at greater than 90%. Pre-ozonation followed by DAF showed reduced CEC removal compared to DAF, followed by intermediate ozonation, demonstrating the importance of removing NOM before this process. TCEP and cotinine were generally observed to be the most recalcitrant indicator compounds, with removals of less than 35% and 80%, respectively, in most of the treatment trains.

In summary, conventional treatment processes provide limited removals. Studies with advanced treatment processes report removals of greater than 80% for virgin GAC, GAC BAFs, ozonation with a dosage at greater than 0.6 mg O_3_/mg TOC, ozonation with H_2_O_2_, UV oxidation without H_2_O_2_ at an intensity greater than 400 mJ/cm^2^, or UV with H_2_O_2_ at an intensity less than 400 mJ/cm^2^. Treatment trains that achieved the greatest removals included DAF in combination with pre- or intermediate ozonation, followed by dual-media filtration and virgin GAC, with most of the compounds removed at greater than 90%. However, fire retardant TCEP (<20%), metabolite cotinine (<40%), pesticides atrazine (generally <80%), DEET (generally <80%), metolachlor (<80%), and X-ray contrast media iopromide (<80%) are still some of the more recalcitrant compounds. Therefore, these CECs are key as the indicator compounds.

### 4.4. Potential Risks to Human Health

As some CECs are recalcitrant through the treatment processes in WTPs, their possible occurrence in drinking water systems is of great concern because of the potential risks to human health. To assess the potential human health concern, Schriks et al. (2010) compared the maximum CEC concentrations observed in a freshwater environment (i.e., surface water, groundwater, or drinking water) to the guideline values obtained from either statutory guidelines or derived from toxicological data relevant to human health reported in the literature [34]. Although all maximum CEC concentrations in the freshwater were less than the guideline values, relatively high potential human health concerns were noted for carbamazepine, 1,4-dioxane, and perfluorooctanesulfonic acid as their freshwater concentrations were at least 20% of the allowed guideline values. The impact of long-term chronic exposure and the synergistic effects of low concentrations of CEC mixtures on human health and potentially sensitive sub-populations remain currently unknown. Murray et al. (2010) calculated a consumption rate posing health risk (CRPHR) for each investigated CEC based on the occurrence in freshwater and the acceptable daily intake (ADI) that considers the uncertainty between effects on animal and human subject [33]. With the CRPHR, 17α-ethinylestradiol, carbamazepine, and 17β-estradiol were found to pose the most potential risk to human health. Similar estimation methods were applied in a number of studies [170,171], though the exposure response to humans was not directly evaluated; rather, quantitative potential risk was calculated [172].

The U.S. EPA established drinking water health advisories for both PFOA and POFS in 2016 [36,37]. Human epidemiology studies have reported an association between PFOA exposure and a series of adverse effects (e.g., high cholesterol, thyroid disorders, pregnancy-induced hypertension, preeclampsia, and testicular and kidney cancer) [36], while exposure to PFOS has been reported to be associated with high cholesterol, thyroid disease, immune suppression, reduced fertility, and fecundity [37]. With the potential adverse effects on human health, the EPA has issued a lifetime drinking water health advisory of 0.07 µg/L for both PFOA and PFOS.

## 5. Properties of CEC Classes

Studying the structure of CECs by class is useful in addressing treatment efficacy. A number of CECs are frequently detected in the water cycle. To better understand persistence, degradability, removals, and also to help reduce the number of compounds with similar properties, the physical and chemical properties of groups of CECs routinely found in systems were reviewed. Classes include those that are frequently studied in WWTP, surface water, and drinking water, such as analgesics, antibiotics, antidepressants, antiepileptic, lipid regulators, CEC metabolites, pesticides, and, psycho-stimulants, those that are persistent in surface water and source water, such as β-blockers and steroids, and those that are recalcitrant during treatment processes, such as fire retardants and X-ray contrast agents.

### 5.1. Analgesics

The great occurrence of analgesics is reported in wastewater, surface water, as well as in drinking water (Figures S1, S15 and S23). Compounds with large K_ow_ values (>10^3^) may be considered relatively hydrophobic, with greater soil/sediment adsorption coefficients and lower solubilities in contrast to compounds with low K_ow_ values (<10) that are very hydrophilic [173,174]. Solubility affects the fate and transport of organic chemicals in the environment. Highly soluble compounds are easily and quickly distributed in the hydrologic cycle and tend to be more readily biodegradable by microorganisms in wastewater treatment plants and surface water. Among the frequently studied analgesics, ibuprofen, ketoprofen, naproxen, and diclofenac have similar structural features (aromatic rings and carboxylic acid). They exhibit similar K_ow_ and pKa values (Table S8) [35,175]. With large K_ow_ values and limited solubilities, biological treatment may not be the most effective treatment for these analgesics. Unlike the above analgesics, acetaminophen has a relatively low K_ow_ value (10^0.46^), suggesting greater biodegradability.

Compounds with larger K_oc_ are more likely to be removed by adsorption. Acetaminophen has relatively greater K_oc_ (170–1300 mL∙g C^−1^) than ibuprofen (18–155 mL∙g C^−1^) [176,177], suggesting that GAC may be effective in removing acetaminophen. Furthermore, acetaminophen has an aromatic ring with a phenolic moiety, suggesting that O_3_ is relatively effective [151,159]. Ibuprofen, with a carboxylic group on its aromatic ring, is an electron-withdrawing functional group that reduces the reactivity of the aromatic ring with ozone [151,178]. The ozone rate constant (k_O3_) for ibuprofen is 9.6 M^−1^ s^−1^, indicating a relatively slow reaction with ozone. In contrast, acetaminophen with a greater rate constant (2.70 × 10^5^ M^−1^ s^−1^) would achieve improved removal through ozonation. With their varying degree of reactivity with ozone and different log K_ow_, acetaminophen and ibuprofen are selected to represent the group of analgesics (Table S8).

### 5.2. Antibiotics

Sufamethoxazole and trimethoprim are the most frequently reported antibiotics in wastewater, surface water, and drinking water (Figures S1, S15 and S23), while erythromycin is observed to be persistent in surface water (Figure S17). From K_ow_ values, erythromycin (10^3.06^) tends to be more hydrophobic, while sulfamethoxazole (10^0.89^) and trimethoprim (10^0.91^) are relatively hydrophilic (Table S9). With low solubility at 1.44 mg/L, erythromycin may be more difficult to biodegrade than the other two antibiotics. Erythromycin, with K_oc_ of 570 mL∙g C^−1^, may be more efficiently removed by GAC than sulfamethoxazole (K_oc_ = 72 mL∙g C^−1^) and trimethoprim (K_oc_ = 75 mL∙g C^−1^) [179]. In addition, the ozonation rate constants for sulfamethoxazole and trimethoprim (Table S9) demonstrate the viability of ozonation. Structures with electron donors are amenable to ozonation [178]. Highly reactive compounds include activated aromatic structures (amine functionalities) [151,166]. The three selected antibiotics have either a primary, secondary, or tertiary amine, suggesting possible locations for ozone to react.

### 5.3. Antidepressants

Diazepam and fluoxetine are the most reported antidepressants, especially in wastewater samples (Figures S5, S18 and S24), while meprobamate is frequently observed in sampled source water and finished drinking water samples (Figure S23). With a low K_ow_ value (~5) as well as excellent solubility (4700 mg/L), meprobamate may possess good biodegradability, while with low solubilities (~55 mg/L), diazepam and fluoxetine may not be easily biodegraded (Table S10) [35,51]. Moreover, the amide structure (electron-drawing moiety) of diazepam may cause low removal efficiency through ozonation, which is also reflected by the low ozone rate constant (k_O3_ = 0.75 ± 0.15 M^−1^ s^−1^) [180]. The secondary amine of fluoxetine and the aliphatic structure of meprobamate suggest their potential reaction with ozonation. With potentially high biodegradability and fast reaction with ozonation, meprobamate is eliminated; diazepam and fluoxetine are selected as the indicator compounds to represent antidepressants.

### 5.4. Antiepileptics

Carbamazepine is the most frequently observed antiepileptic. Carbamazepine has aromatic rings and amide (Table S11). Amide is not as reactive with ozone because of its electron-drawing nature, causing lower removal efficiencies [151]. However, carbamazepine has a relatively high rate constant (k_O3_ = 3 × 10^5^ M^−1^ s^−1^), demonstrating that it is a reactive compound with ozone. This high reactivity of carbamazepine can be attributed to the reactivity of ozone with the (electron donor) double bond that connects the two phenyl moieties [180]. The relatively large K_ow_ and lower aqueous solubility of carbamazepine suggest potential poor biodegradability. Other than ozonation, GAC may be a possible removal process for carbamazepine, because it has a K_oc_ of 510 mL∙g C^−1^ [179].

### 5.5. β-Blockers

The most frequently detected β-blockers are atenolol, metoprolol, and propranolol (Figures S9, S19 and S26). These β-blockers have similar structures (with an aromatic ring, carboxylic acid, ether, and amines); they also have similar properties (Table S12). Amine functionalities are structural components of β-blockers that render the compounds highly reactive with respect to ozone [181]. Atenolol has k_O3_ of 1.7 × 10^3^ M^−1^ s^−1^, suggesting a moderate reaction rate with ozonation. The rate constant for propranolol with ozonation is 1.0 × 10^5^ M^−1^ s^−1^, two orders of magnitude greater than that for atenolol, suggesting an increased rate of reaction with ozone. However, the log K_ow_ ranges from 0.16 to 3.48 (Table S12), with varying aqueous solubility as well, indicating potentially unique behavior. Moreover, atenolol has a reported K_oc_ ranging from 148 to 1700 mL∙g C^−1^ [177], indicating potentially significant removal through GAC. With their varying degrees of reactivity with ozone and different log K_ow_, atenolol and propranolol are selected to represent the group of β-blockers (Table S12).

### 5.6. Lipid Regulators

Clofibric acid and gemfibrozil are two of the most frequently reported lipid regulators in studies (Figures S10, S19 and S27). The K_ow_ varies with clofibric acid (10^2.88^) and gemfibrozil (10^4.77^), respectively [51,175], indicating hydrophobicity (Table S13). Compared to clofibric acid, gemfibrozil has a relatively lower solubility (19 mg/L), suggesting poor biodegradability. Therefore, gemfibrozil is selected as an indicator compound. Gemfibrozil, with a K_oc_ of 430 mL∙g C^−1^ [179], may be easily removed through GAC. Gemfibrozil has structural features, including an aromatic ring, a carboxyl group, and ether (Table S13). The carboxyl group is an electron-withdrawing functional group, indicative of a potentially lower efficiency with ozonation [178]. The rate constant for gemfibrozil is 2.0 × 10^3^ M^−1^ s^−1^ (Table S13), moderately reactive with ozone [151].

### 5.7. Pesticides

Atrazine, metolachlor, and DEET were the most frequently detected pesticides (Figures S12 and S29); moreover, they are observed to be recalcitrant in treatment processes as well. Atrazine has a triazine ring but does not have aromatic moieties (electron donors), indicating a slower reaction with ozone, as indicated by the rate constant (k_O3_ = 6.0–7.9 M^−1^ s^−1^) (Table S14). Furthermore, amide moieties are not reactive with ozone [178]. Both metolachlor and DEET have amide on their aromatic rings, suggesting that they may be difficult to break down with ozone. Atrazine has a large K_ow_ and low aqueous solubility, which demonstrates its hydrophobicity and relatively low biodegradability. On the other hand, though metolachlor and DEET have relatively greater aqueous solubilities than atrazine, their K_ow_ values are still very large, suggesting poor biodegradability. Moreover, atrazine has a K_oc_ ranging from 23 to 101 mL∙g C^−1^, lower than the K_oc_ reported for DEET (300 mL∙g C^−1^) [179,182,183], suggesting lower removal through GAC. With different properties, atrazine, metolachlor, and DEET are selected as the indicator compounds.

### 5.8. Steroids

Cholesterol, coprostanol, 17β-estradiol, and estrone are the most frequently detected steroids in the aquatic environment (Figures S14, S22 and S32). Because cholesterol and coprostanol have similar structures (alcohol and aliphatic rings), and 17β-estradiol and estrone share similar structures (phenol and aliphatic rings), cholesterol and 17β-estradiol are selected to be representative of groups of steroids. The properties of cholesterol and 17β-estradiol reveal that both have aliphatic moieties, expected to react with molecular ozone, with large rate constants [184] (Table S15). Both steroid hormones and natural steroids have high K_ow_ values, indicating hydrophobicity. The aqueous solubilities of these two steroids are also relatively low, suggesting resistance to biodegradability. These properties demonstrate resistance to biological treatment and persistence in water systems. Compared to other compounds studied in this work, 17β-estradiol has a relatively high K_oc_ (3700–10,000 mL∙g C^−1^) [185,186,187], suggesting potentially significant removal through GAC.

### 5.9. Other Classes

Fire retardant TCEP (removal < 20%), nicotine metabolite cotinine (removal < 40%), and X-ray contrast agent iopromide (removal < 80%) are recalcitrant through treatment processes, while the occurrence of psycho-stimulant caffeine and surfactants PFOA and PFOS is frequently reported (Figures S1, S15 and S23). Therefore, they are selected as the indicator compounds. TCEP, caffeine, cotinine, and iopromide have small K_ow_ values (Table S16), which suggests that they are hydrophilic compounds, and yet they are frequently detected in water systems, which may result from their broad usage. GAC may not be an effective treatment process for these six compounds, as K_oc_ values for TCEP, caffeine, PFOA, PFOS, and cotinine are 67, 22, 29.51, 125.89, and 130 mL∙g C^−1^, respectively, and iopromide has a reported K_oc_ value of as low as 0.005 mL∙g C^−1^ [150,179,185]. TCEP has an aliphatic structure with chlorine functional groups, which is polar. TCEP has been shown to be resistant to oxidation using chlorine or ozone [151]. Caffeine has a k_O3_ of 6.4 × 10^2^ M^−1^ s^−1^ (Table S16), indicating a relatively slower reaction with ozone. Caffeine has a purine base (xanthine ring), alkyl groups, and a C=C double bond. The C=C double bond and alkyl are electron donors, which are amenable to ozonation [178]. PFOA and PFOS have carbon–fluorine (C-F) bonds in their structure. With the most electronegative element fluorine, C-F is a strong bond, which makes PFOA and PFOS two stable CECs and resistant to oxidation and biodegradation [36]. Cotinine has ketone and a pyridine ring. The k_O3_ of iopromide is relatively small, less than 0.8 M^−1^ s^−1^. Therefore, the reaction of ozone with iopromide is very slow. Iopromide exhibits three nitrogen atoms as amides, which have very low reactivity to ozone [180].

## 6. Conclusions

Based on the criteria of usage, occurrence, persistence in aquatic environments, resistance to treatment, and physicochemical properties, 22 CECs belonging to 13 classes were identified as priority indicator compounds, including:

1. Analgesics: acetaminophen and ibuprofen;
2. Antibiotics: erythromycin, sulfamethoxazole, and trimethoprim;
3. Antidepressants: diazepam and fluoxetine;
4. Antiepileptics: carbamazepine;
5. β-blockers: atenolol and propranolol;
6. Blood lipid regulators: gemfibrozil;
7. Fire retardants: TCEP;
8. Nicotine metabolites: cotinine;
9. Pesticides: atrazine, metolachlor, and DEET;
10. Steroids: 17β-estradiol and cholesterol;
11. Surfactants: PFOA and PFOS;
12. Psycho-stimulant: caffeine;
13. X-ray contrast agent: iopromide.

These indicator compounds represent the large number of CECs widely used, are observed at great frequency in aqueous systems, are resistant to treatment, are persistent in the environment, and represent a suite of classes of compounds. Although CECs and therefore indicator compounds have been observed under dilute concentrations (in ng/L to μg/L) in the water cycle, their effects in the environment cannot be neglected.

The indicator compounds were applied in bench and pilot plant studies to examine the treatability of CECs through conventional and advanced water treatment processes. In our pilot plant study [152], this list was used to understand the optimal combination of existing unit treatment processes in WTP for the removal of CECs through studies of 12 treatment trains. Acetaminophen, ibuprofen, sulfamethoxazole, trimethoprim, carbamazepine, gemfibrozil, caffeine, and 17β-estradiol achieved greater than 75% removal in all treatment trains. The most recalcitrant CECs included TCEP and cotinine, with removals of less than 35% and 80%, respectively, in most of the trains. The 90% removals were achieved for a number of compounds using trains that included advanced treatment. Specifically, DAF (with either pre- or intermediate oxidation) followed by dual-media filtration and UV/H_2_O_2_, removed 80% of the compounds at greater than 90%. However, 90% removal for all compounds in a single study was only achieved with trains involving DAF in combination with pre- or intermediate ozonation followed by dual-media filtration and virgin GAC. To address the effectiveness of using a more sustainable treatment process that is less energy-intensive and therefore more economical, Zhang et al. (2017) applied bench-scale BAFs as a function of media type, ozonation, and EBCTs [165]. Ibuprofen, trimethoprim, and 17β-estradiol were removed on average at greater than 80%, while iopromide was the most recalcitrant compound, with removals of 59% under optimal conditions (i.e., EBCT: 18 min, media: GAC; and pre-ozonation: 3 or 4 mg/L). Applying indicator compounds representative of the vast array of CECs observed throughout the water cycle reduces the number of compounds that need to be analyzed and tracked in addressing the efficacy of treatment processes as well as studying transport and fate in the environment.

## Figures and Tables

**Table 1:** Removal efficacy of CECs in WWTPs.

(a) Unit Processes					
Class	Compound	Clarification [69,75,111,112]	Activated Sludge [69,111,114,118,119,120]	MBBR [81,113,121]	Filtration [69]
Analgesic	Acetaminophen	1–8%	92–99%	-	99%
	Diclofenac	0%	0–5%	-	-
	Ibuprofen	0–32%	71–99%	94%	-
	Naproxen	9%	56–99%	70–80%	-
Antibiotic	Sulfamethoxazole	17–34%	0–69%	0%	89%
	Trimethoprim	0%	0–5%	36%	-
Antidepressant	Diazepam	36%	-	-	-
	Fluoxetine	-	23–62%	-	-
Antiepileptic	Carbamazepine	0%	0–10%	0–11%	0%
Antihypertension	Diltiazem	0%	13%	-	-
Antiseptic	Triclosan	28%	55–99%	-	-
β-blocker	Atenolol	9%	14%	40%	-
	Metoprolol		7%	-	-
	Propranolol	0%	1%	-	-
Lipid Regulator	Gemfibrozil	-	23–76%	-	-
CEC Metabolite	Cotinine	-	54%	-	-
Pesticide	Atrazine	-	-	8%	-
Psycho-stimulant	Caffeine	0%	95–99%	-	99%
**(b) Whole trains in pilot- and full-scale WWTPs**					
**Class**		**Compound**		**Removal** [75,78,115,116,122]	
Analgesic		Acetaminophen		98–99%	
		Diclofenac		18%	
		Ibuprofen		70–95%	
		Naproxen		43–88%	
Antibiotic		Sulfamethoxazole		63%	
		Trimethoprim		58%	
Antidepressant		Diazepam		41–55%	
		Fluoxetine		72%	
Antiepileptic		Carbamazepine		0–42%	
Antihypertension		Diltiazem		22%	
Antiseptic		Triclosan		69–88%	
β-blocker		Atenolol		41–50%	
		Metoprolol		61%	
		Propranolol		0%	
Lipid Regulator		Gemfibrozil		52–88%	
CEC Metabolite		Cotinine		78%	
Pesticide		Atrazine		68%	
Psycho-stimulant		Caffeine		44–86%	

**Table 2:** CEC removal in conventional treatment processes.

Compounds	Coagulation [148,149,150,151,152]	Filtration [148,152]	Chlorination [148,152,153,154]
			Contact Time: 60 min
**Analgesics**			
Acetaminophen	-	0%	59–62%
Diclofenac	<10%	-	-
Hydrocodone	24%	-	-
Ibuprofen	<10%	0–12%	3–22%
Naproxen	<5%		23%
**Antibiotic**			
Erythromycin	-	0–7%	8%
Sulfamethazine	-	12%	0%
Sulfamethoxazole	-	0–21%	0%
Trimethoprim	-	0–43%	-
**Antidepressant**			
Fluoxetine	<15%	-	-
**Antiepileptic**			
Carbamazepine	<5%	0–7%	0–14%
**Antiseptic**			
Triclosan	-	-	36%
**Beta-Blocker**			
Atenolol	-	0–22%	19%
Metoprolol	-	-	0%
**Fire Retardant**			
TCEP	0%	0%	0%
**Lipid Regulator**			
Atorvastatin	<10%	-	-
Clofibric Acid	-	-	5%
Gemfibrozil	<5%	2–14%	22%
**Metabolite**			
Cotinine	-	0%	0%
**Pesticide**			
Atrazine	0–10%	0%	0%
DEET	0%	0–14%	0%
**Plasticizer**			
BPA	<5%		11%
**Steroid**			
Estradiol	2%	0%	58%
Estriol	-	-	53%
Estrone	5%	-	36%
Ethynylestradiol	0%	-	41%
**Stimulants**			
Caffeine	0%	0–44%	9–59%
**Surfactant**			
PFOA	<12.5%	0%	10%
PFOS	<12.5%	0%	0%
**X-Ray Contrast Media**			
Iopromide	-	0–27%	0%

**Table 3:** CEC removals in advanced treatment processes.

Compounds	GAC [148,152,155,158] Virgin	GAC [104,155] w/o Reg.	GAC BAF [158]	DM or Sand BAF [156,157]	Ozone w/o H_2_O_2_ [152,159]			Ozone w/H_2_O_2_ [49]		UV w/o H_2_O_2_ [51,148]		UV w/H_2_O_2_ [51]
	EBCT (min)				O_3_/TOC *			H_2_O_2_/TOC *		40 mJ/cm^2^	439 mJ/cm^2^	372 mJ/cm^2^–5 mg/L
	6–7.6	3–15	9–18	5–15.8	0.2–0.3	0.6	1.0	0.2	0.7			
**Analgesics**												
Acetaminophen	90%	25–99%	-	59–79%	>99%	-	-	88%	-	20–50%	>80%	>80%
Diclofenac	-	69%	98–99%	21–28%	20–95%	95–99%	>99%	-	>99%	50–80%	>80%	>80%
Hydrocodone	-	-	-	95%	-	-	-	-	-	<20%	>80%	>80%
Ibuprofen	90%	16%	-	70–95%	10–72%	30–99%	90–95%	80%	83%	<20%	20–50%	>80%
Naproxen	-	6%	90–98%	50–90%	20–99%	>99%	>99%	-	>99%	<20%	>80%	>80%
**Antibiotic**												
Erythromycin	90%	8%	85–98%	15–27%	91%	93%	-	-	-	<20%	50–80%	50–80%
Sulfamethoxazole	90%	84%	96–98%	2.4–4.1%	25–99%	>95%	>99%	99%	98%	50–80%	>80%	>80%
Trimethoprim	90%	64%	96–98%	83–92%	50–99%	93–99%	>99%	95%	>99%	<20%	20–50%	>80%
**Antidepressant**												
Diazepam	-	-	-	-	20–30%	65–75%	90–99%	-	>90%	<20%	<20%	50–80%
Fluoxetine	-	-	-	-	25–60%	90–99%	>99%	-	>99%	<20%	>80%	>80%
Meprobamate	90%	13%	-	-	10–25%	35–55%	60–80%	-	80%	<20%	<20%	20–50%
**Antiepileptic**												
Carbamazepine	90%	16%	90–88%	0.5–1.6%	50–99%	91–99%	>99%	87–99%	>99%	<20%	20–50%	>80%
Dilantin	90%	8%	-	-	0–55%	50–85%	90–95%	-	-	<20%	50–80%	>80%
**Antiseptic**												
Triclosan	-	0%	-	90%	45–95%	>95%	>95%	-	>99%	50–80%	>80%	>80%
**Beta-Blocker**												
Atenolol	90%	62%	40–70%	-	20–50%	65–99%	>99%	85%	99%	-	-	-
Metoprolol	-	40%	50–80%	-	-	-	-	-	-	-	-	-
**Fire Retardant**												
TCEP	90%	0%	-	-	0–10%	0–10%	10%	17–21%	13%	<20%	<20%	<20%
**Lipid Regulator**												
Clofibric Acid	-	-	-	35–52%	-	-	-	-	-	-	-	-
Gemfibrozil	90%	8%	80–97%	70–94%	30–91%	82–99%	>99%		>99%	<20%	20–50%	>80%
**Metabolite**												
Cotinine	90%	-	-	23–39%	0%	32%	-	13%	-	-	-	-
**Pesticide**												
Atrazine	90%	3%	-	0.2–3%	5–25%	0–50%	60–99%	75%	69%	<20%	50–80%	50–80%
DEET	70–90%	43–63%	-	15–70%	20–69%	31–75%	85–95%	99%	90%	<20%	<20%	50–80%
Metolachlor	90%	-	-	6.6–8.7%	-	-	-	-	-	<20%	<20%	50–80%
**Plasticizer**												
BPA	-	-	-	-	-	-	-	-	>78%	-	-	-
**Steroid**												
Estradiol	90%	-	-	-	>99%	98%	-	97%	>83%	<20%	>80%	>80%
Estriol	-	0%	-	-	-	-	-	-	-	<20%	>80%	>80%
Estrone	-	-	-	-	65–99%	>99%	>99%	-	>98%	<20%	>80%	>80%
Ethynylestradiol	-	-	-	12–22%	-	-	-	-	-	<20%	>80%	>80%
Progesterone	-	-	-	-	-	-	-	-	-	<20%	20–50%	>80%
Testosterone	-	74%	-	-	-	-	-	-	-	<20%	20–50%	>80%
**Stimulants**												
Caffeine	90%	16%	10–50%	67–80%	20–55%	39–99%	>99%	99%	>74%	<20%	<20%	50–80%
**Surfactants**												
PFOA	64–90%	-	-	-	-	-	-	-	-	<20%	-	-
PFOS	81–90%	-	-	-	-	-	-	-	-	<20%	-	-
**X-ray Contrast Media**												
Iopromide	90%	8%	-	3–13%	5–39%	20–40%	60–80%	29–49%	-	<20%	50–80%	50–80%

* Value indicates mass ratio; Reg.—regeneration; DM—dual media.

## Data Availability

Not applicable.

## Supplementary Materials

The following are available online at https://www.mdpi.com/1660-4601/18/3/1288/s1.

## Nomenclature

ADI	acceptable daily intake
BAF	biologically active filter
C-F bond	carbon–fluorine bond
CECs	contaminants of emerging concern
CRPHR	consumption rate posing health risk
DAF	dissolved air flotation
DDE	dichlorodiphenyl-dichloroethylene
DEET	*N*,*N*-diethyl-meta-toluamide
EBCT	empty bed contact time
EDC	endocrine-disrupting chemicals
GAC	granular activated carbon
ICM	iodinated contrast media agents
k_O3_	O_3_ rate constant
MBBR	moving bed biofilm reactor
NOM	natural organic matter
OH•	hydroxyl radical
PFAS	per- and polyfluoroalkyl substances
PFOA	perfluorooctanoic acid
PFOS	perfluorooctane sulfonate
TCEP	tri(2-chloroethyl) phosphate
TCPP	tris(1-chloro-2-propyl) phosphate
TOC	total organic carbon
USGS	U.S. Geological Survey
WTP	water treatment plants
WWTP	wastewater treatment plant
