# Supplementary material for: Indicator Compounds Representative of Contaminants of Emerging Concern (CECs) Found in the Water Cycle in the United States
Source: Int J Environ Res Public Health. 2021 Feb 1;18(3):1288. doi: 10.3390/ijerph18031288 (PMC7908579; doi:10.3390/ijerph18031288)
Supplement: Supplementary file 1 [file ijerph-18-01288-s001.pdf]

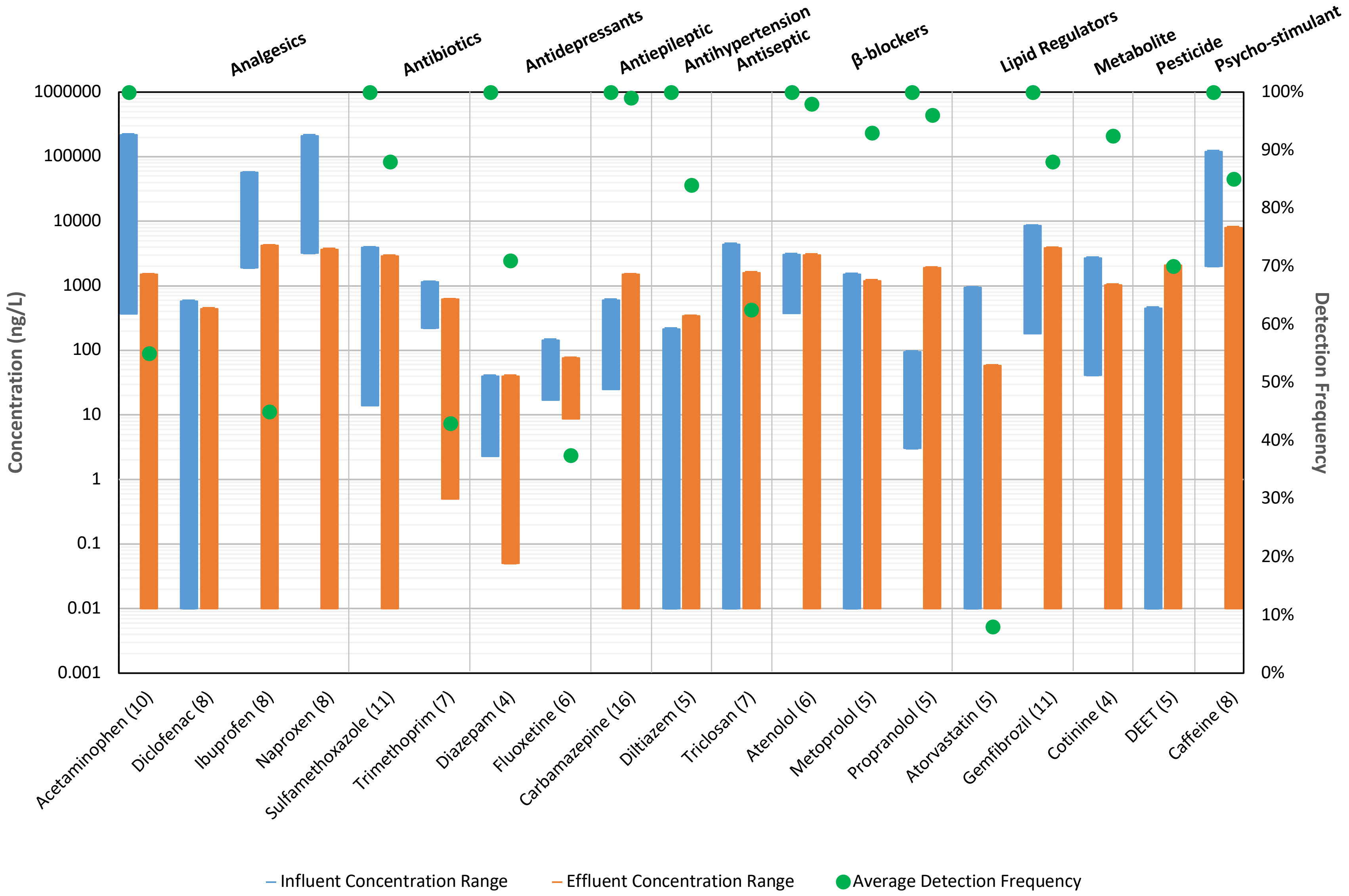

Figure S1. Concentration range of the most studied CECs in WWTP influents (blue bar) and effluents (orange bar) and their average detection frequency (green dots). Number of studies on the compounds is indicated in the parentheses (data from Table S5).

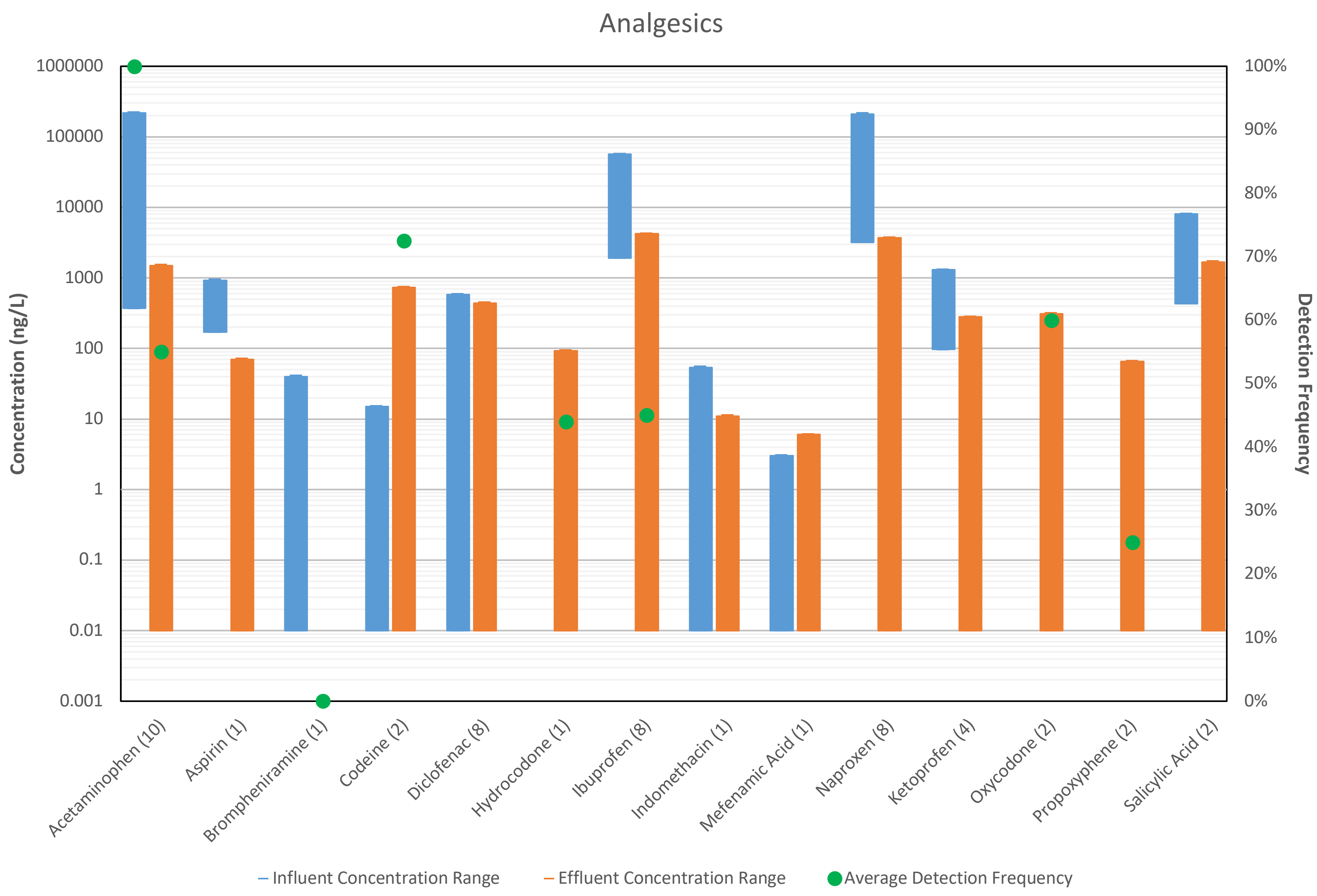

Figure S2. Concentration range of analgesics detected in WWTP influents (blue bar) and effluents (orange bar) and their average detection frequency (green dots). Number of studies on the compounds is indicated in the parentheses (data from Table S5).

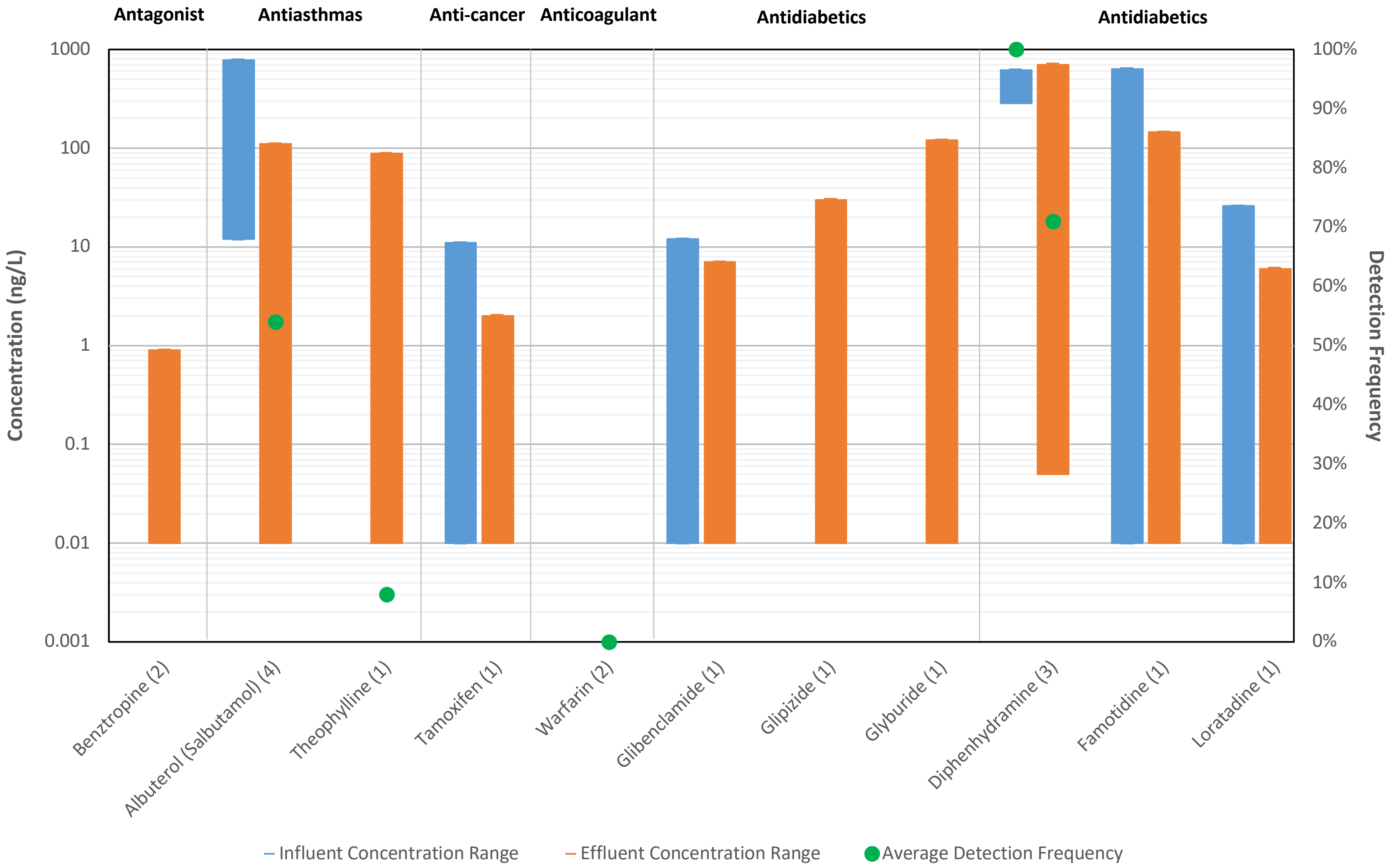

Figure S3. Concentration range of antagonist, antiathmas, anti-cancer, anticoagulant, antidiabetics, and antidiabetics detected in WWTP influents (blue bar) and effluents (orange bar) and their average detection frequency (green dots). Number of studies on the compounds is indicated in the parentheses (data from Table S5).

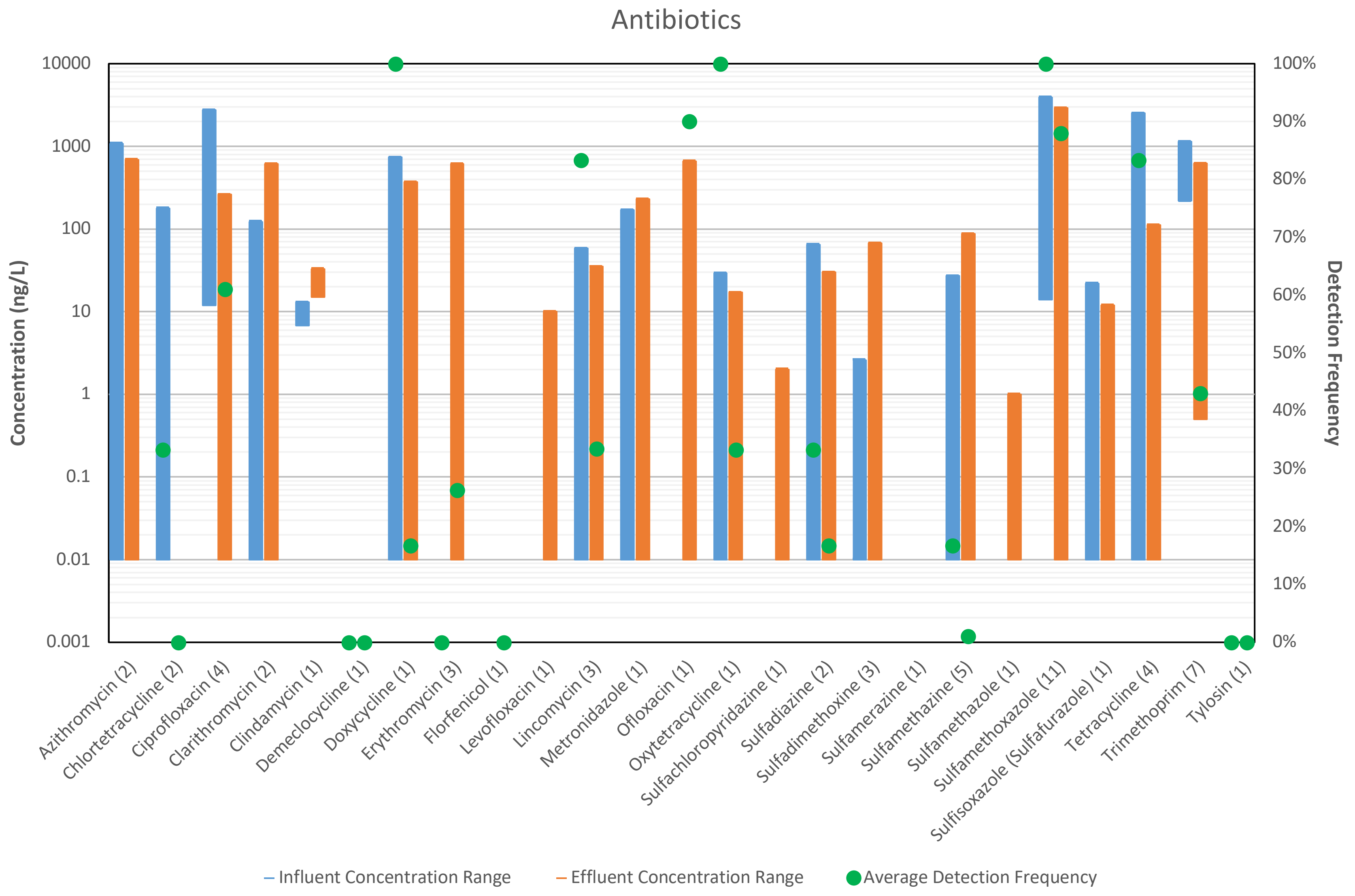

Figure S4. Concentration range of antibiotics detected in WWTP influents (blue bar) and effluents (orange bar) and their average detection frequency (green dots). Number of studies on the compounds is indicated in the parentheses (data from Table S5).

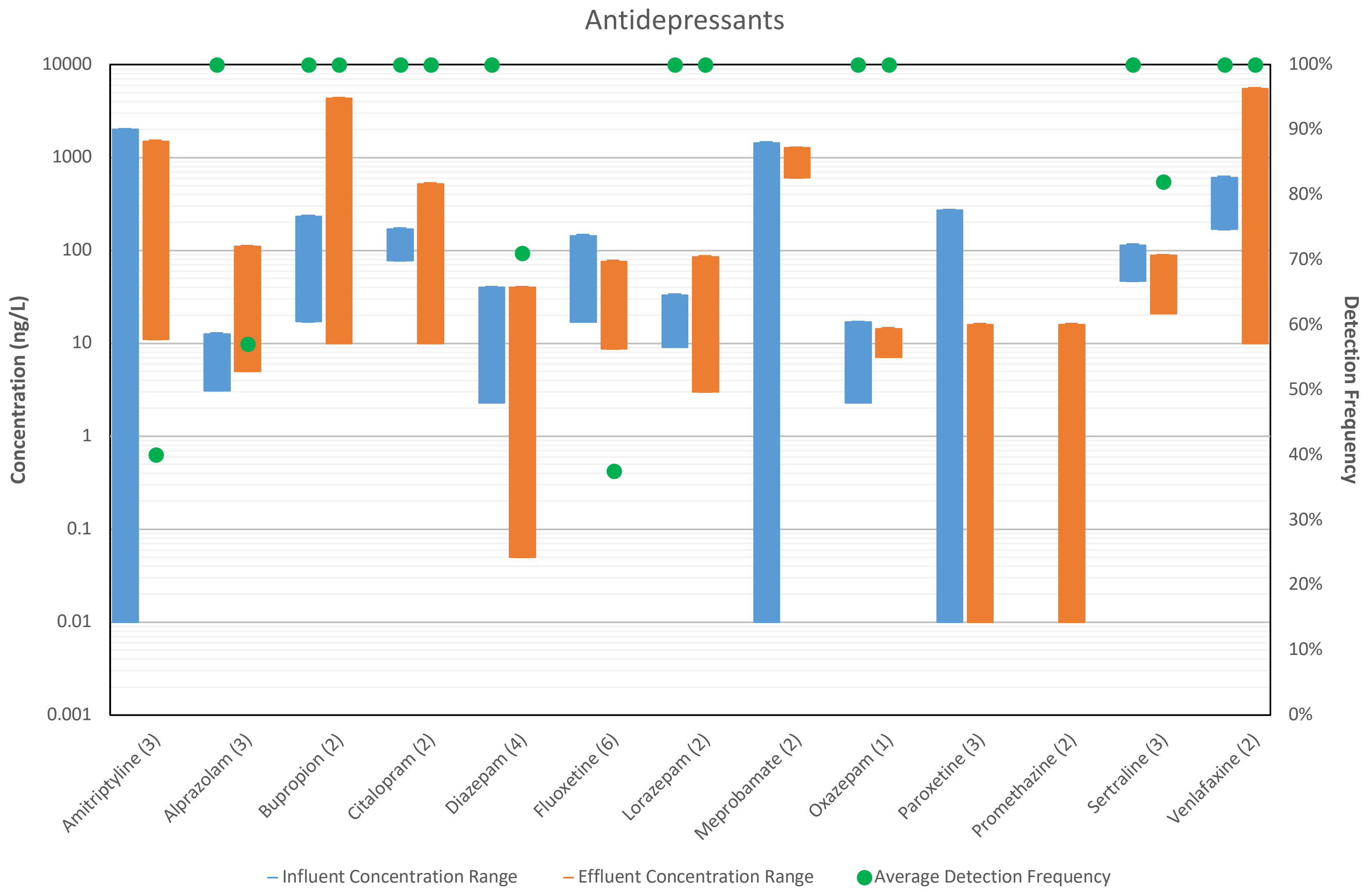

Figure S5. Concentration range of antidepressants detected in WWTP influents (blue bar) and effluents (orange bar) and their average detection frequency (green dots). Number of studies on the compounds is indicated in the parentheses (data from Table S5).

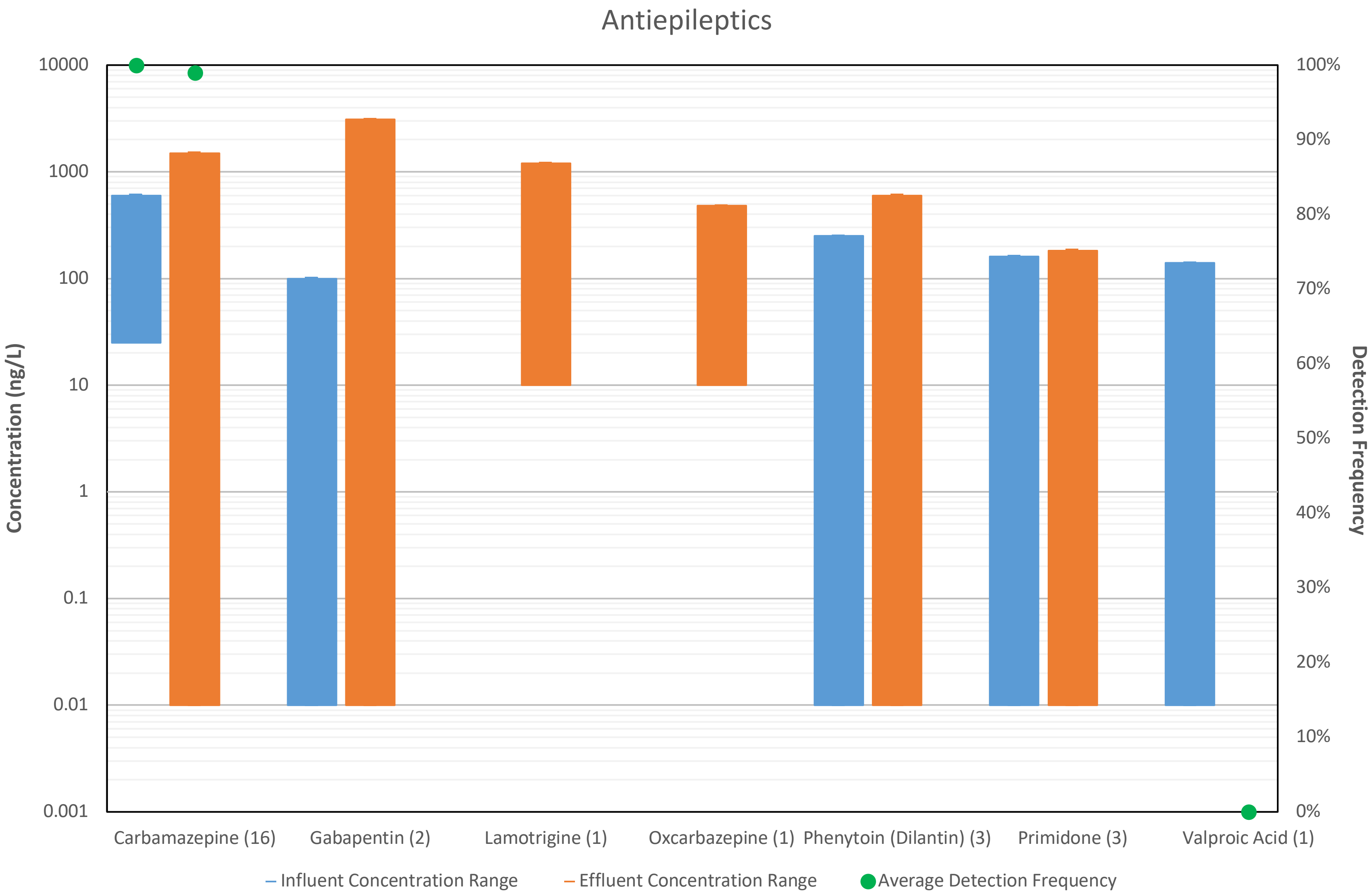

Figure S6. Concentration range of antiepileptics detected in WWTP influents (blue bar) and effluents (orange bar) and their average detection frequency (green dots). Number of studies on the compounds is indicated in the parentheses (data from Table S5).

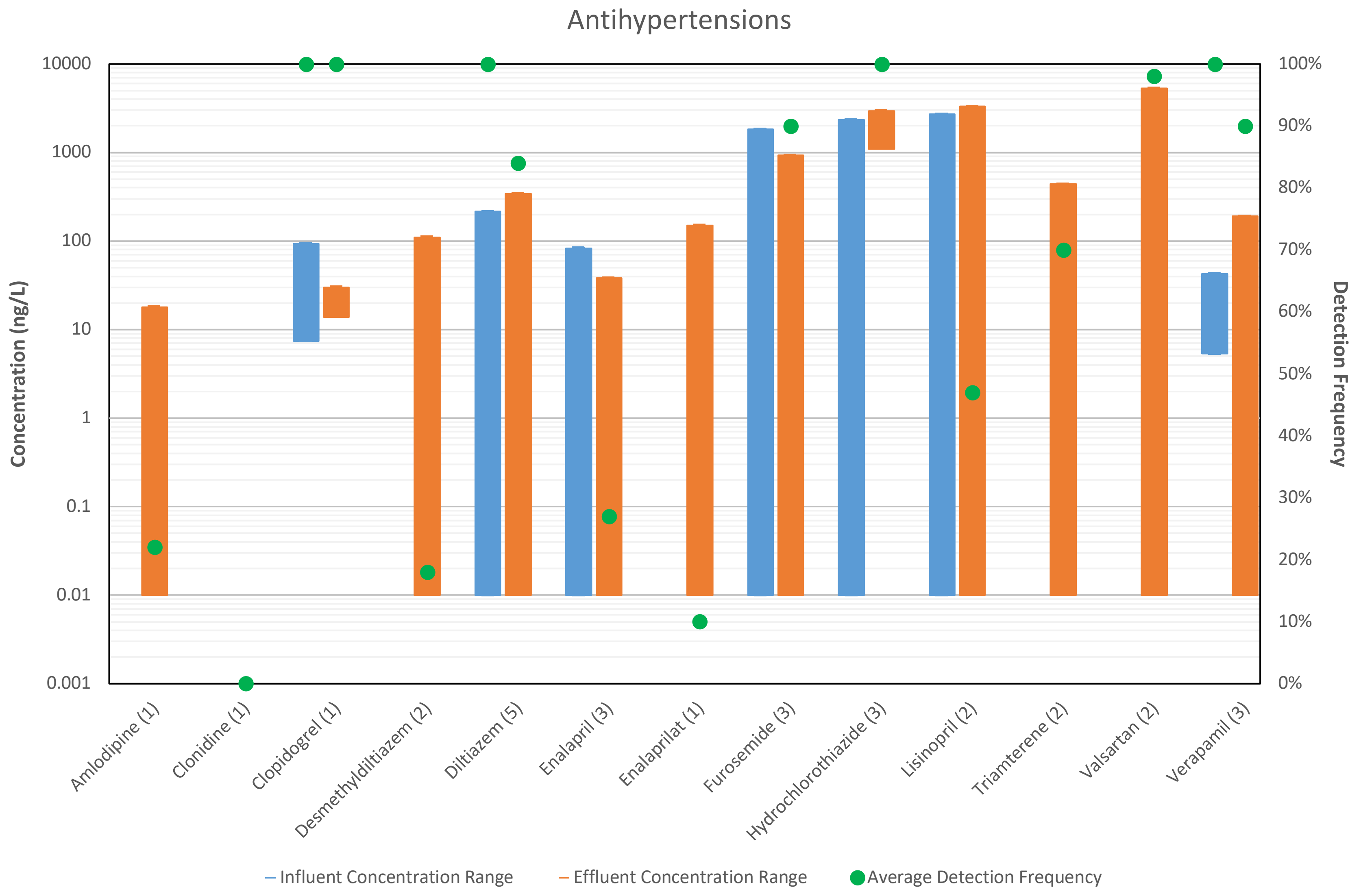

Figure S7. Concentration range of antihypertensions detected in WWTP influents (blue bar) and effluents (orange bar) and their average detection frequency (green dots). Number of studies on the compounds is indicated in the parentheses (data from Table S5).

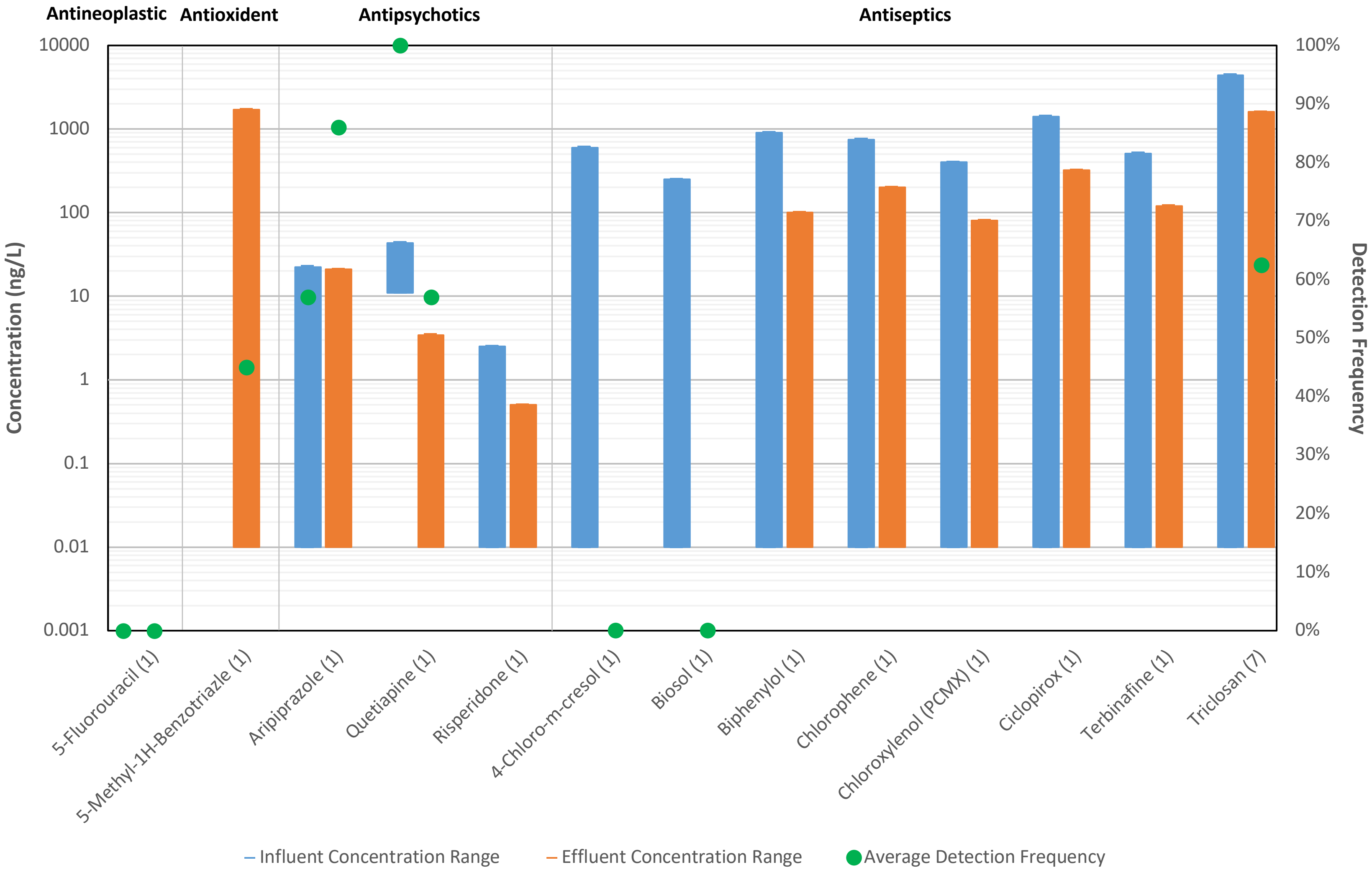

Figure S8. Concentration range of antineoplastic, antioxidant, antipsychotics, and antiseptics detected in WWTP influents (blue bar) and effluents (orange bar) and their average detection frequency (green dots). Number of studies on the compounds is indicated in the parentheses (data from Table S5).

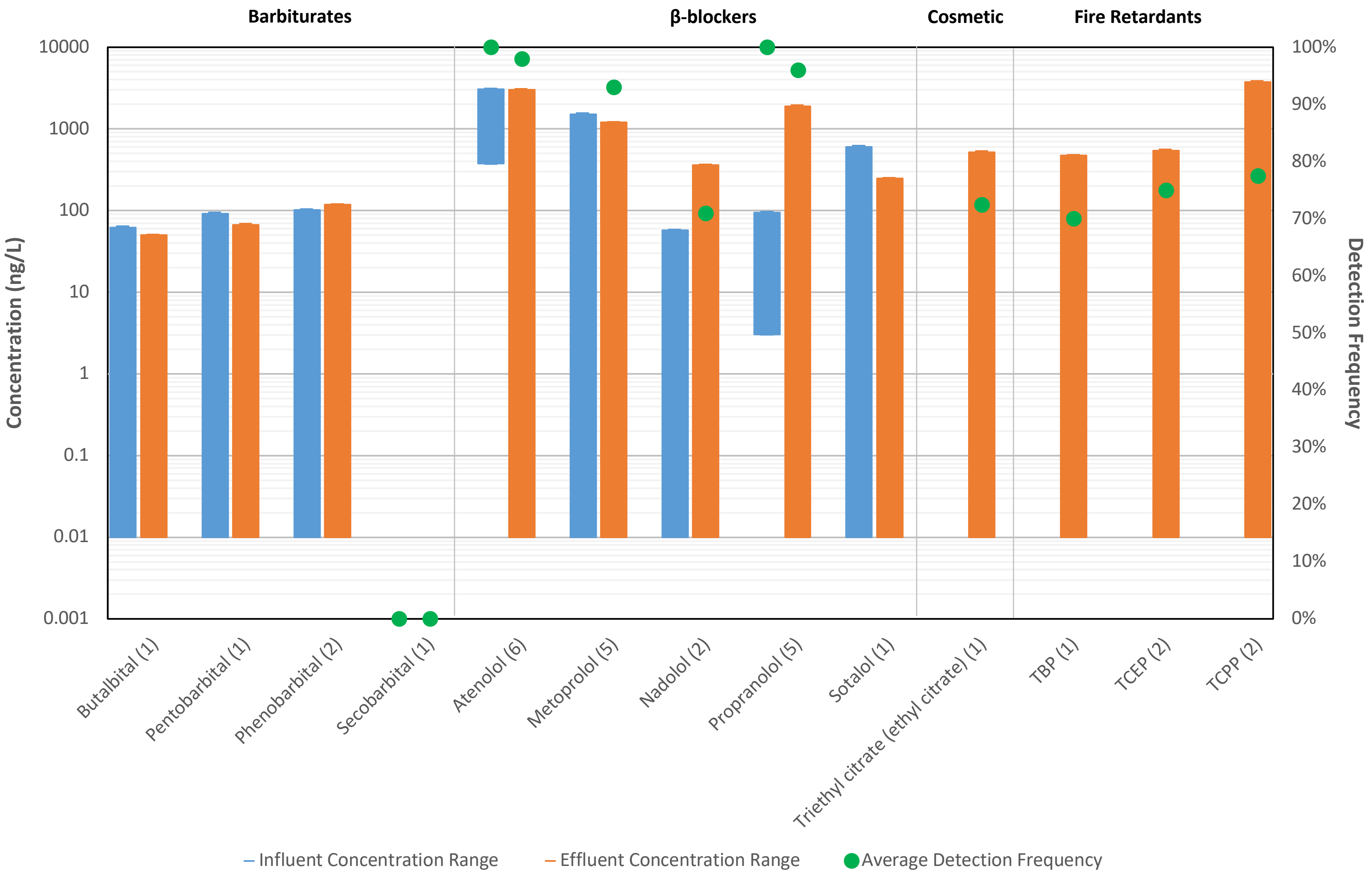

Figure S9. Concentration range of barbiturates, β-blockers, cosmetic, and fire retardants detected in WWTP influents (blue bar) and effluents (orange bar) and their average detection frequency (green dots). Number of studies on the compounds is indicated in the parentheses (data from Table S5).

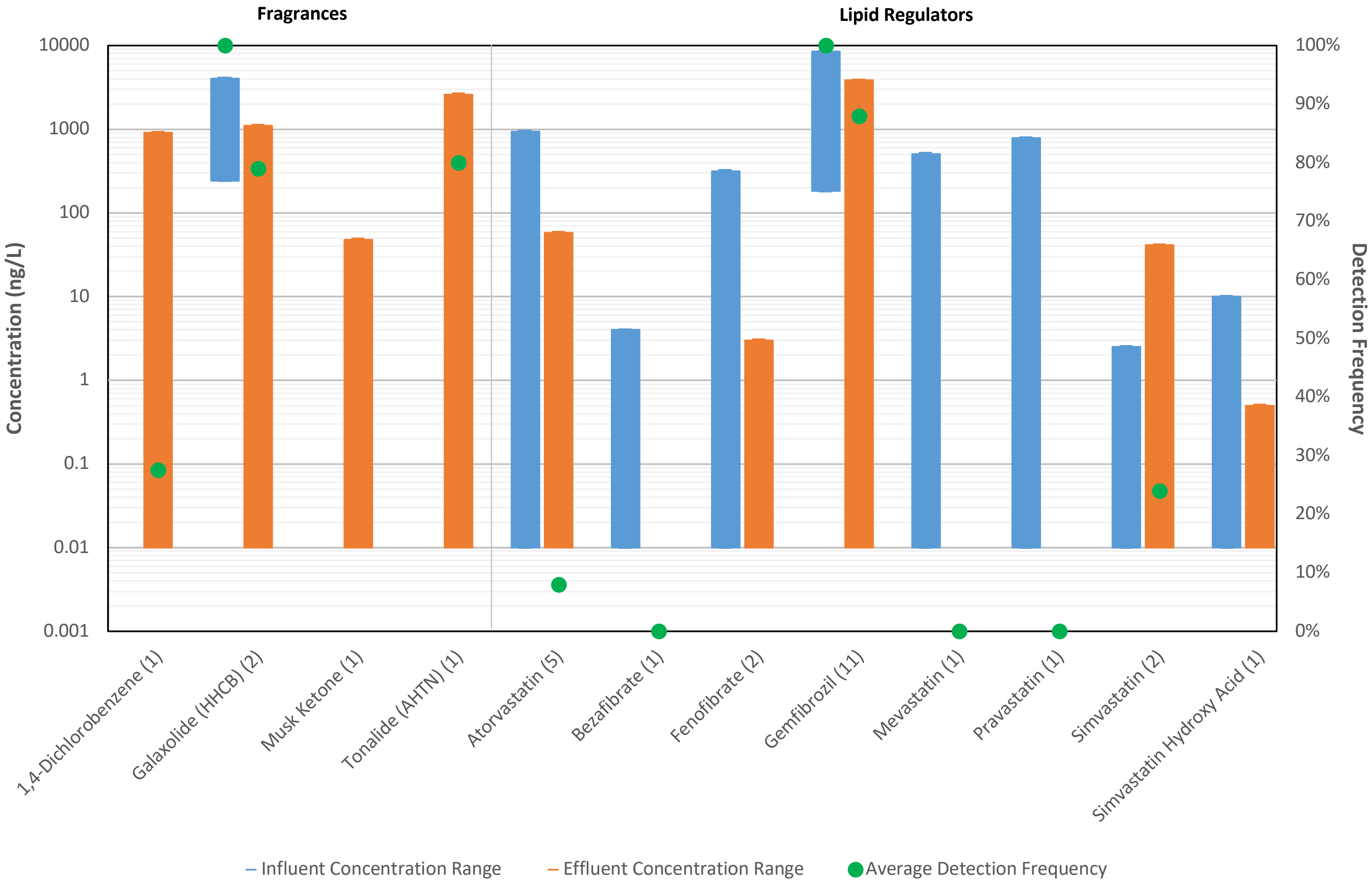

Figure S10. Concentration range of fragrances and lipid regulators detected in WWTP influents (blue bar) and effluents (orange bar) and their average detection frequency (green dots). Number of studies on the compounds is indicated in the parentheses (data from Table S5).

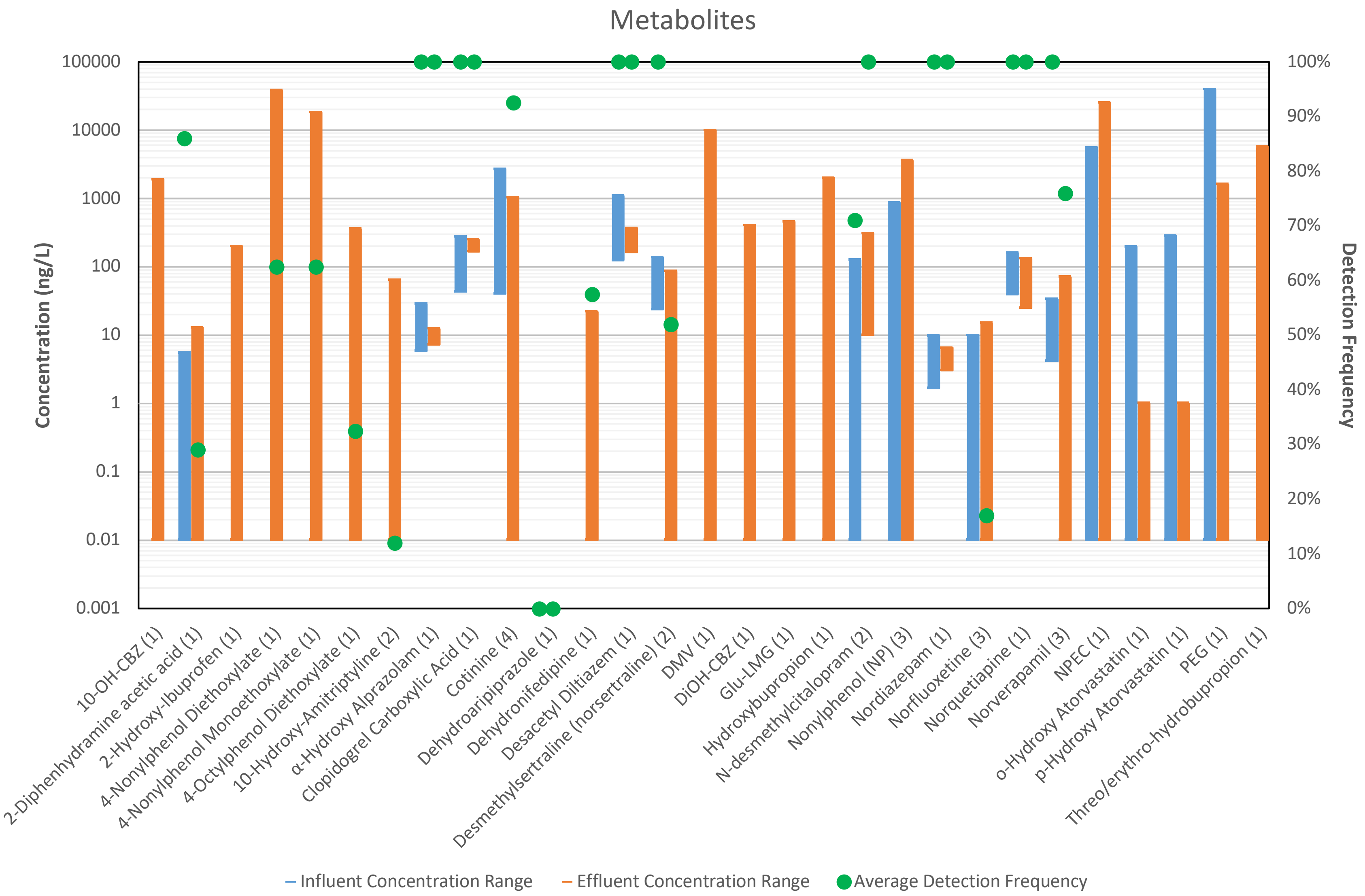

Figure S11. Concentration range of metabolites detected in WWTP influents (blue bar) and effluents (orange bar) and their average detection frequency (green dots). Number of studies on the compounds is indicated in the parentheses (data from Table S5).

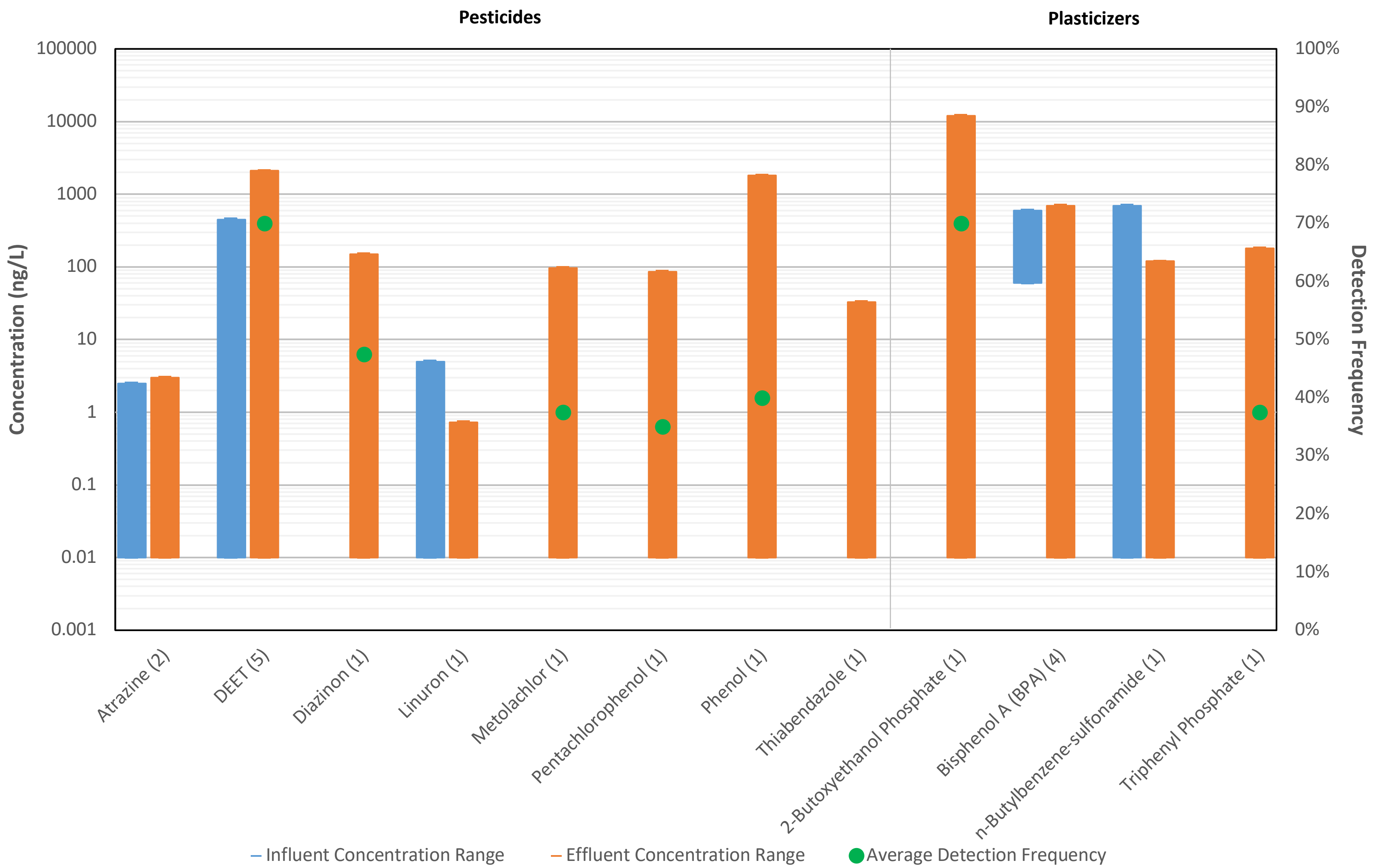

Figure S12. Concentration range of pesticides and plasticizers detected in WWTP influents (blue bar) and effluents (orange bar) and their average detection frequency (green dots). Number of studies on the compounds is indicated in the parentheses (data from Table S5).

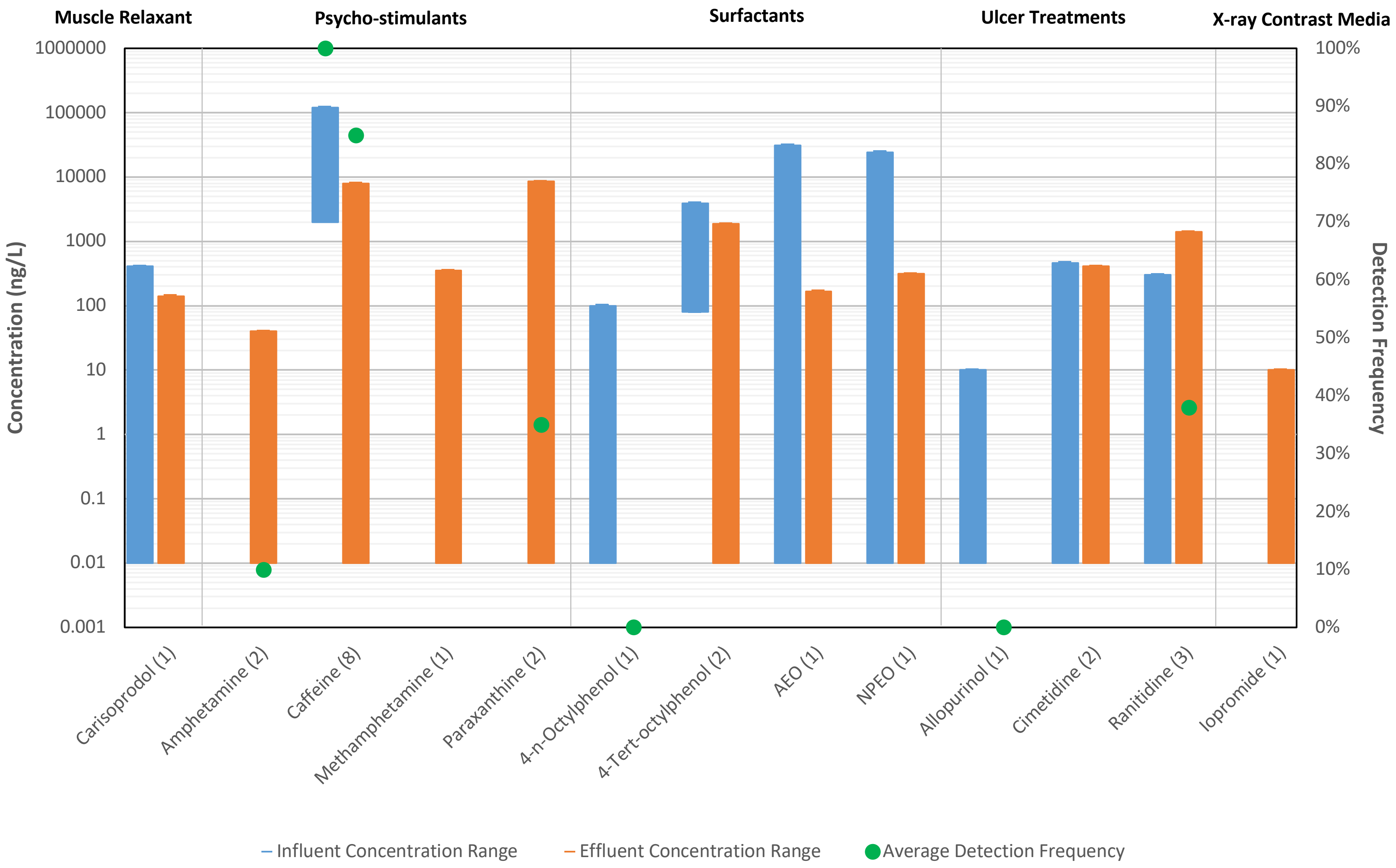

Figure S13. Concentration range of muscle relaxant, psycho-stimulants, surfactants, ulcer treatment, and x-ray contrast detected in WWTP influents (blue bar) and effluents (orange bar) and their average detection frequency (green dots). Number of studies on the compounds is indicated in the parentheses (data from Table S5).

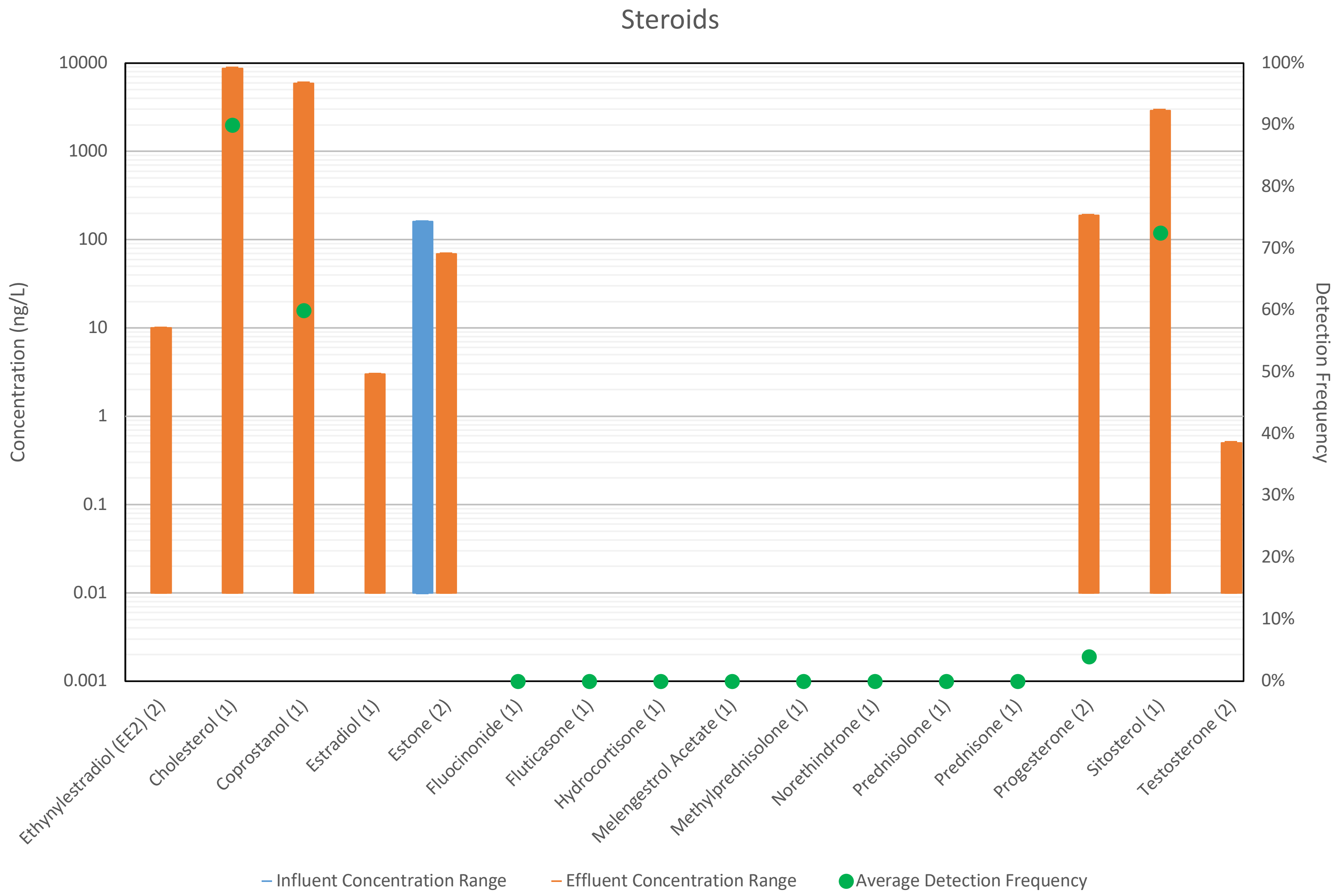

Figure S14. Concentration range of steroids detected in WWTP influents (blue bar) and effluents (orange bar) and their average detection frequency (green dots). Number of studies on the compounds is indicated in the parentheses (data from Table S5).

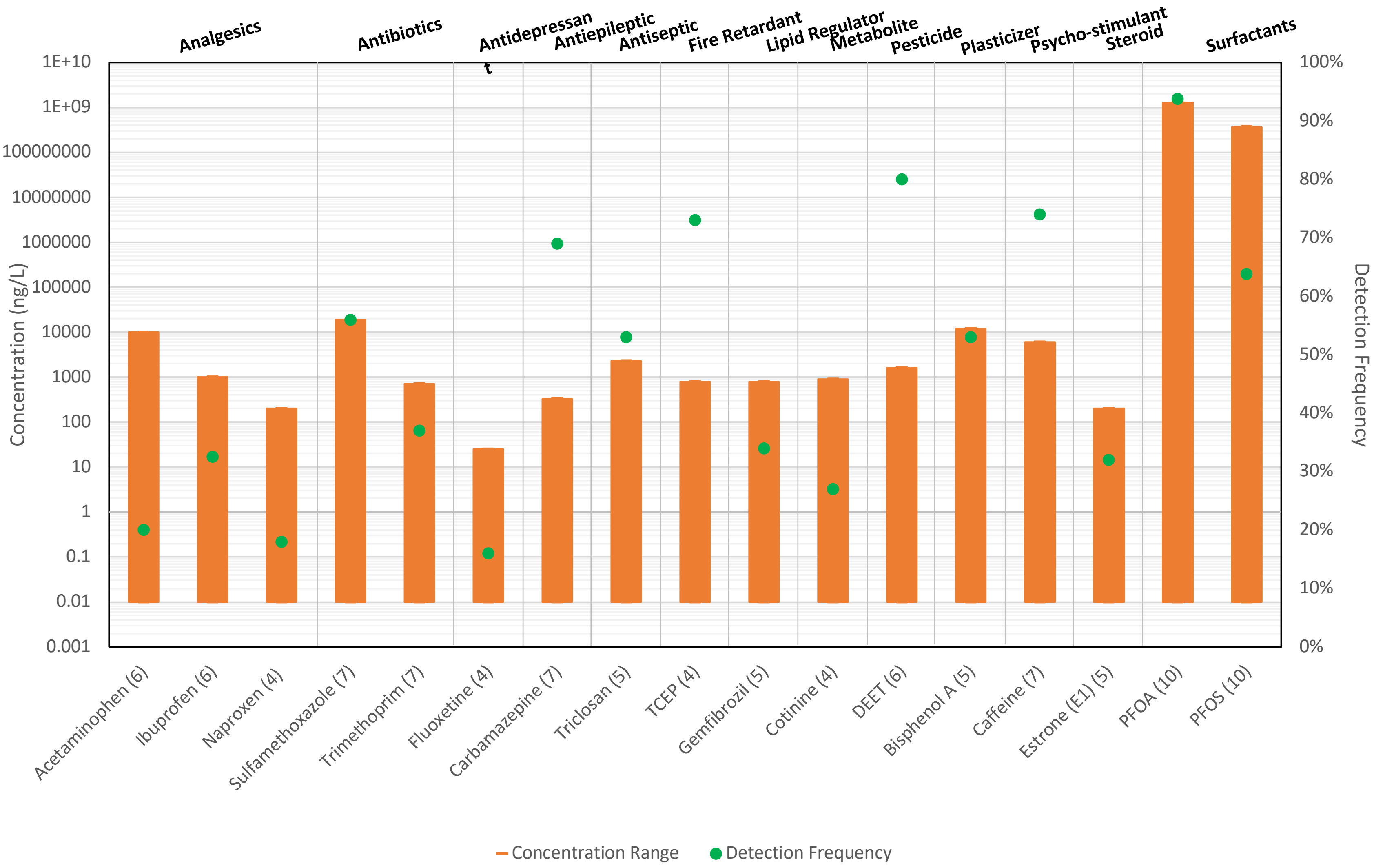

Figure S15. Concentration range of the most studied CECs in surface water (orange bar) and their average detection frequency (green dots). Number of studies on the compounds is indicated in the parentheses (data from Table S6).

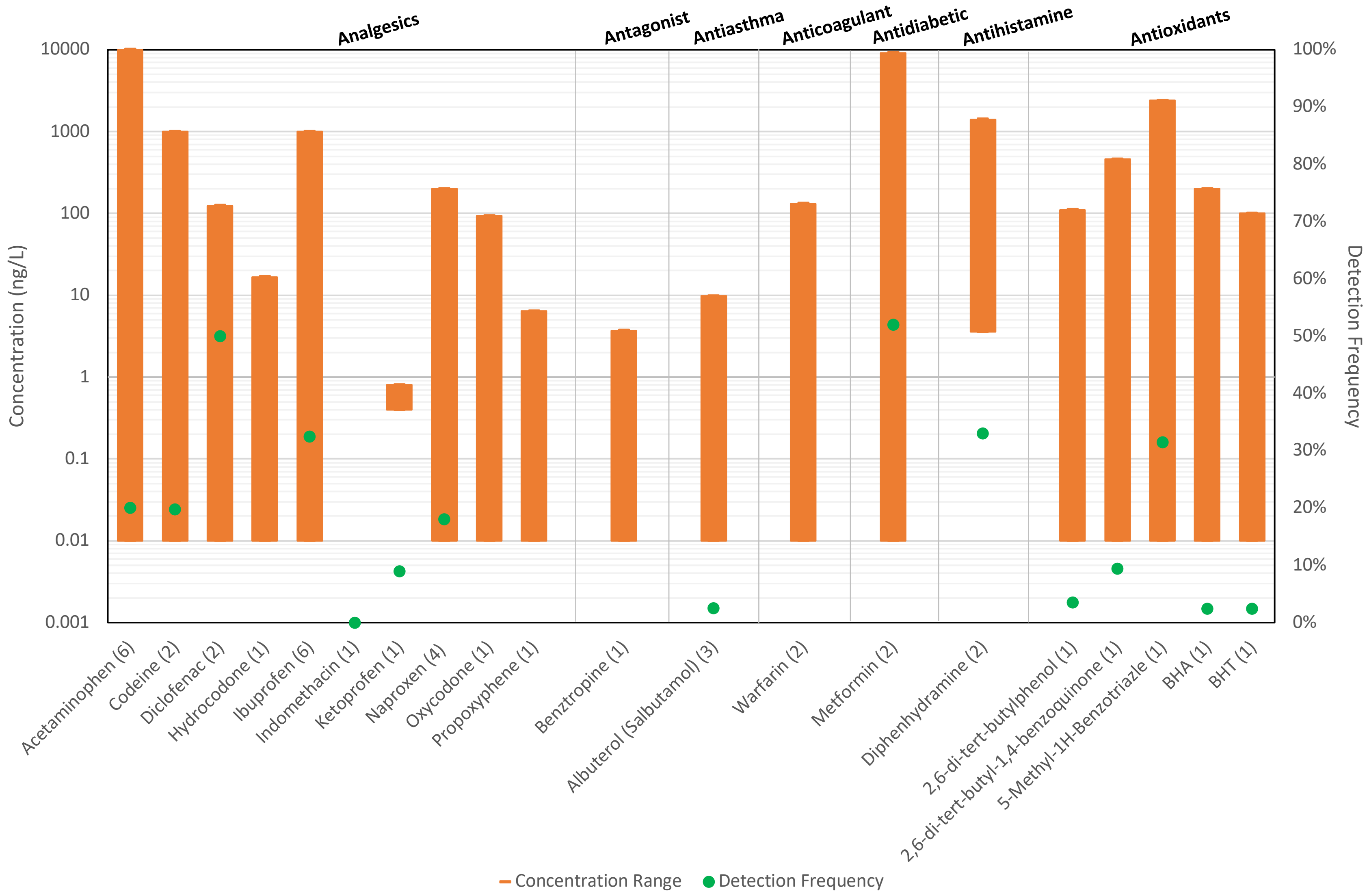

Figure S16. Concentration range of analgesics, antagonist, antiasthma, anticoagulant, antidiabetic, antihistamine, and antioxidants detected in surface water (orange bar) and their average detection frequency (green dots). Number of studies on the compounds is indicated in the parentheses (data from Table S6).

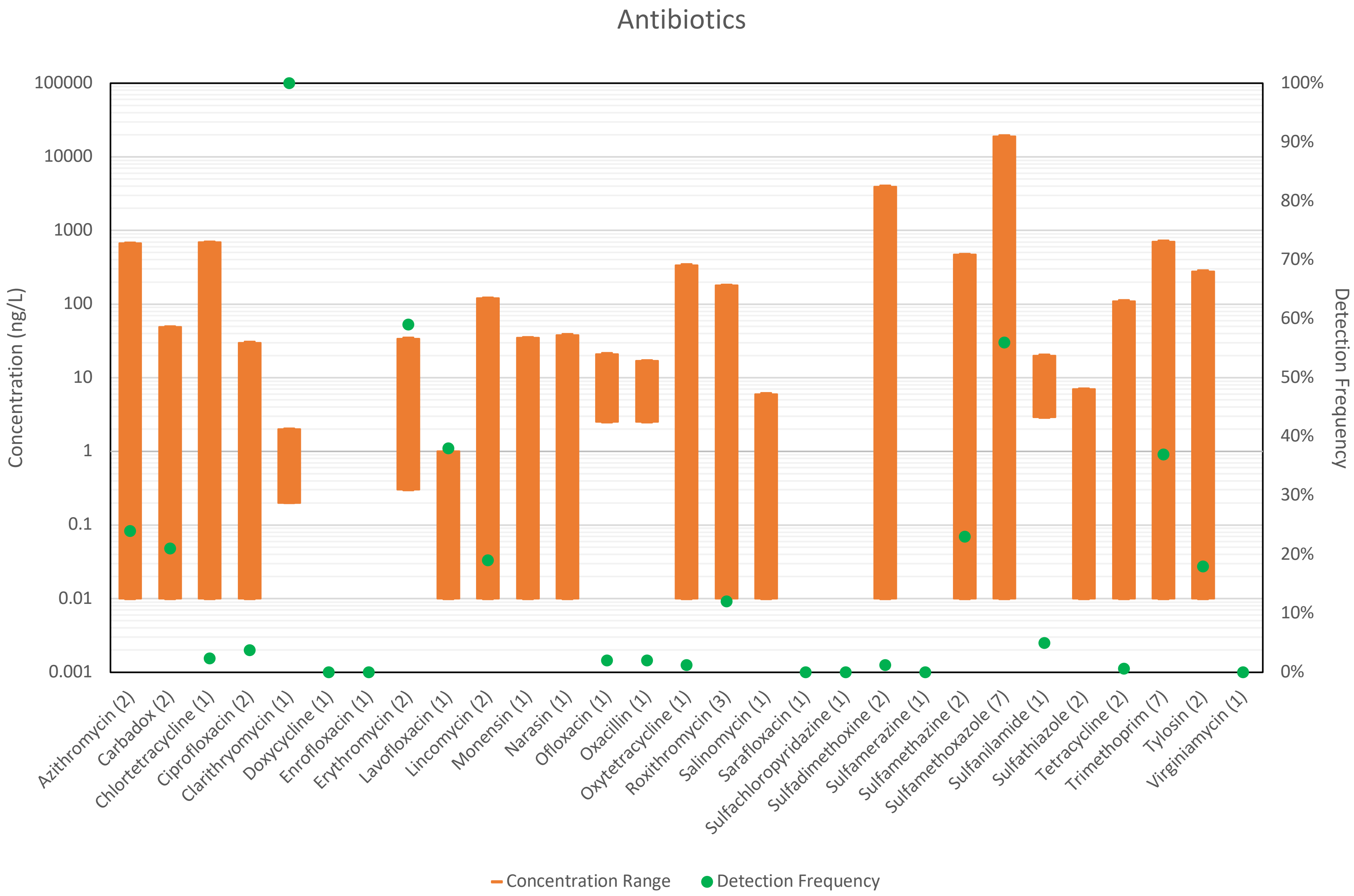

Figure S17. Concentration range of antibiotics detected in surface water (orange bar) and their average detection frequency (green dots). Number of studies on the compounds is indicated in the parentheses (data from Table S6).

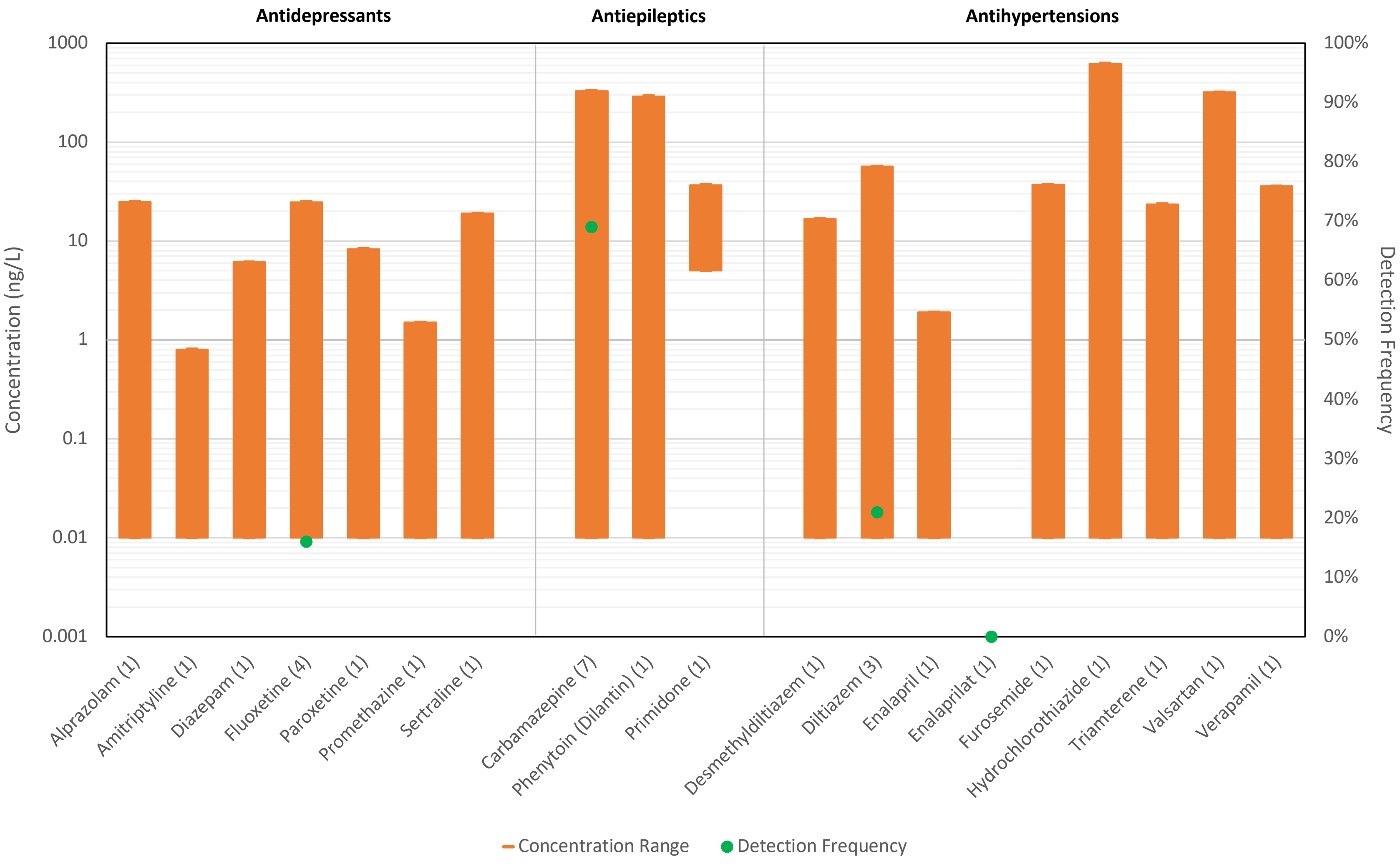

Figure S18. Concentration range of antidepressants, antiepileptics, and antihypertensions detected in surface water (orange bar) and their average detection frequency (green dots). Number of studies on the compounds is indicated in the parentheses (data from Table S6).

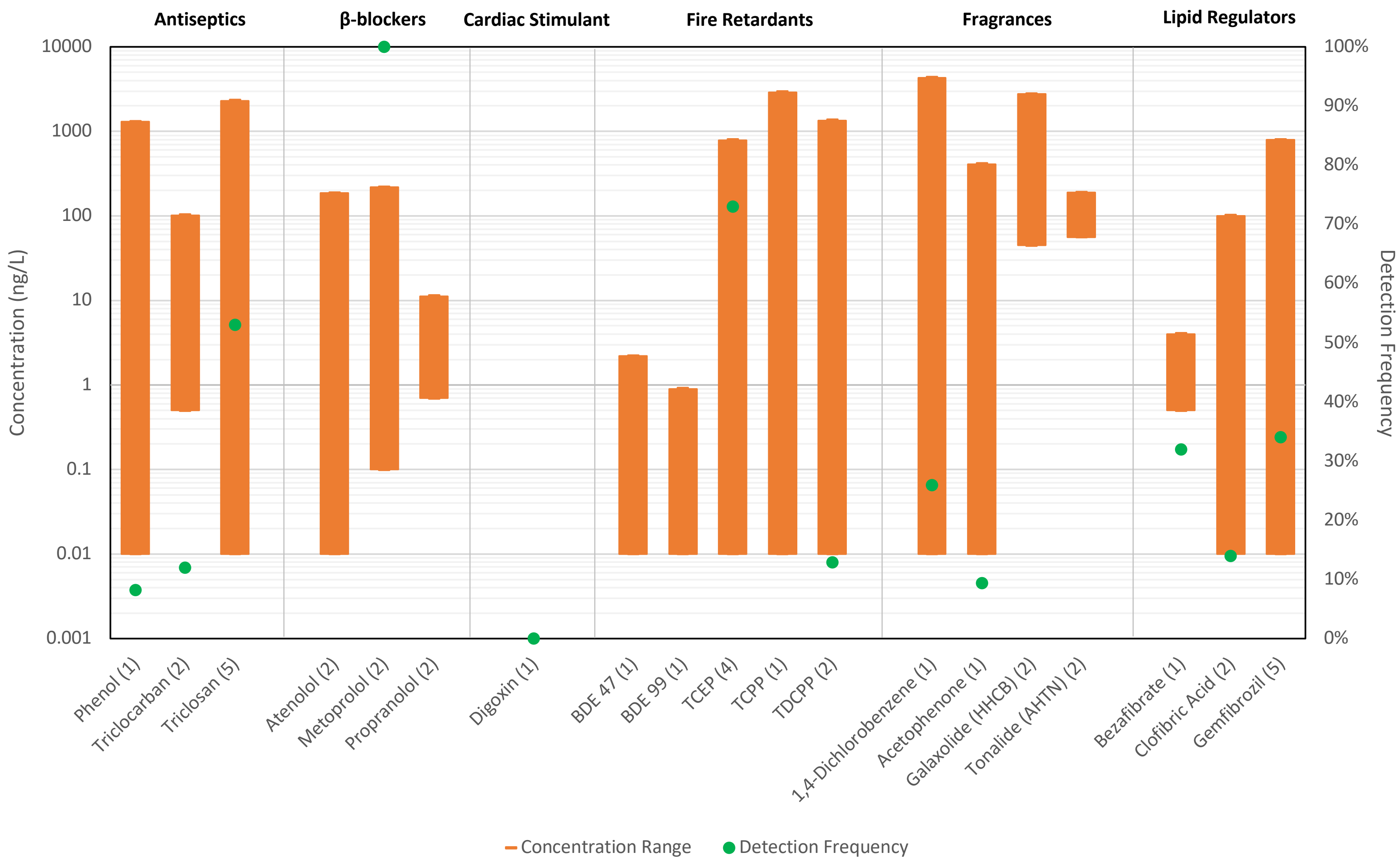

Figure S19. Concentration range of antiseptics, β-blockers, cardiac stimulant, fire retardants, fragrances, and lipid regulators detected in surface water (orange bar) and their average detection frequency (green dots). Number of studies on the compounds is indicated in the parentheses (data from Table S6).

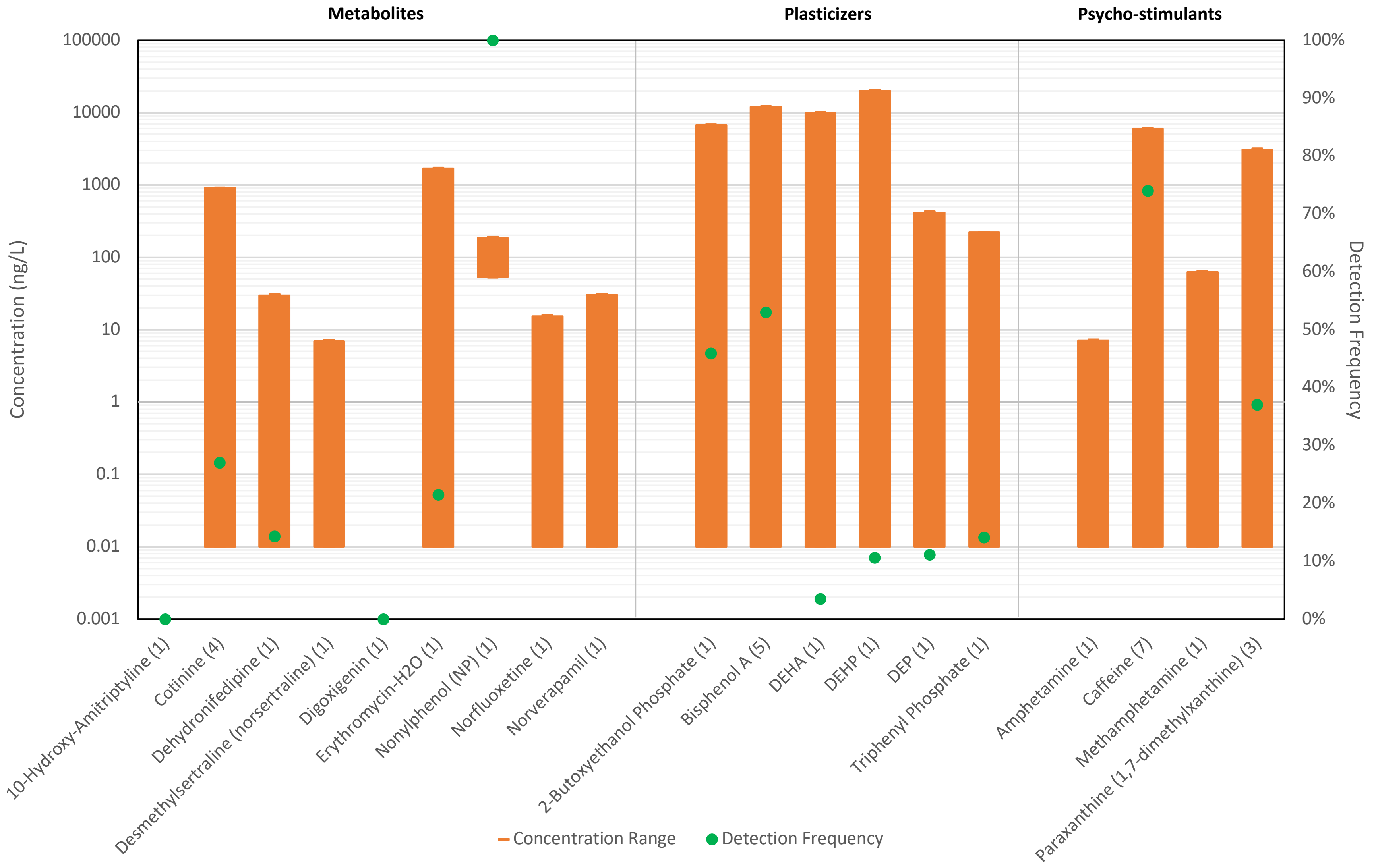

Figure S20. Concentration range of metabolites, plasticizers, and psycho-stimulants detected in surface water (orange bar) and their average detection frequency (green dots). Number of studies on the compounds is indicated in the parentheses (data from Table S6).

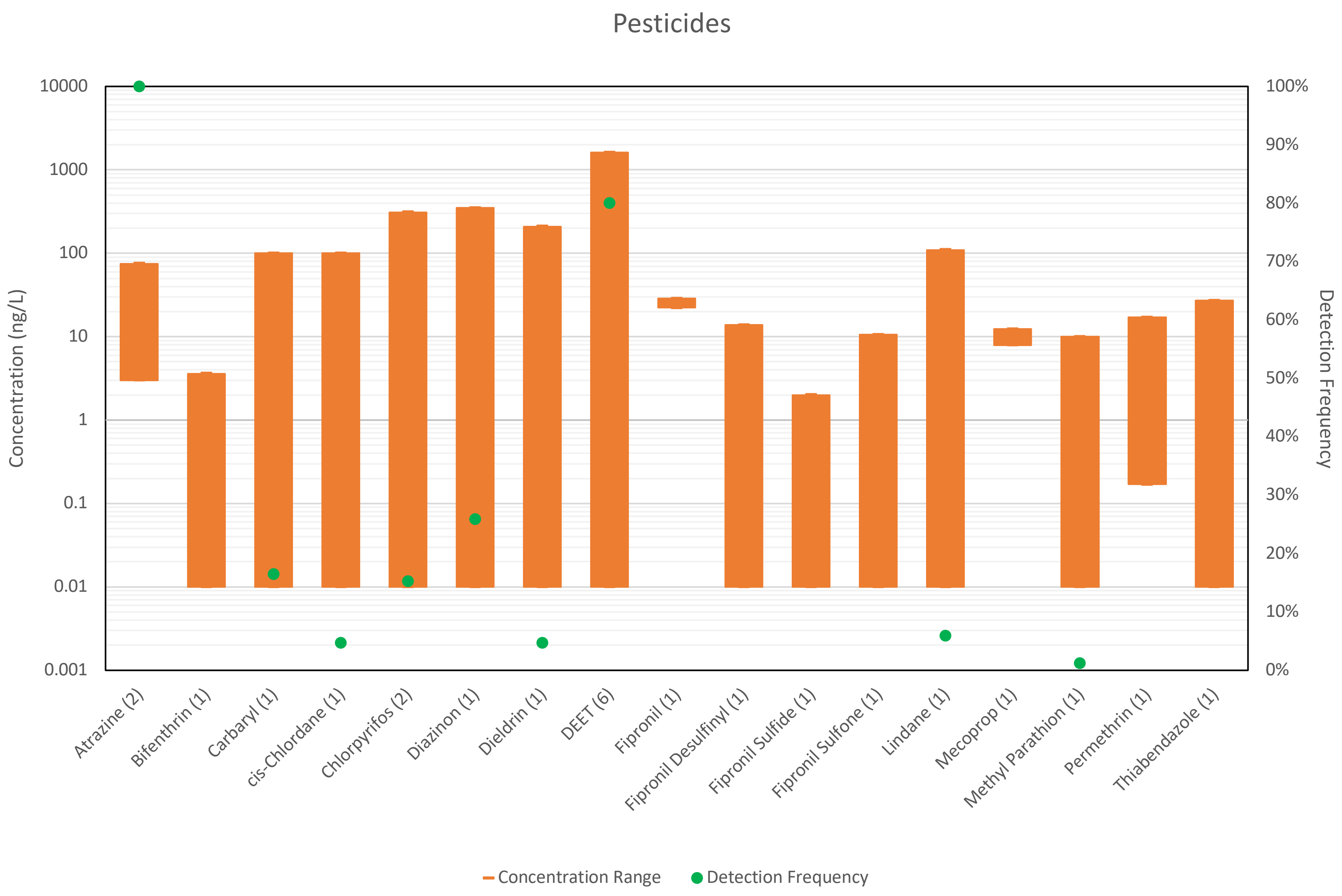

Figure S21. Concentration range of pesticides detected in surface water (orange bar) and their average detection frequency (green dots). Number of studies on the compounds is indicated in the parentheses (data from Table S6).

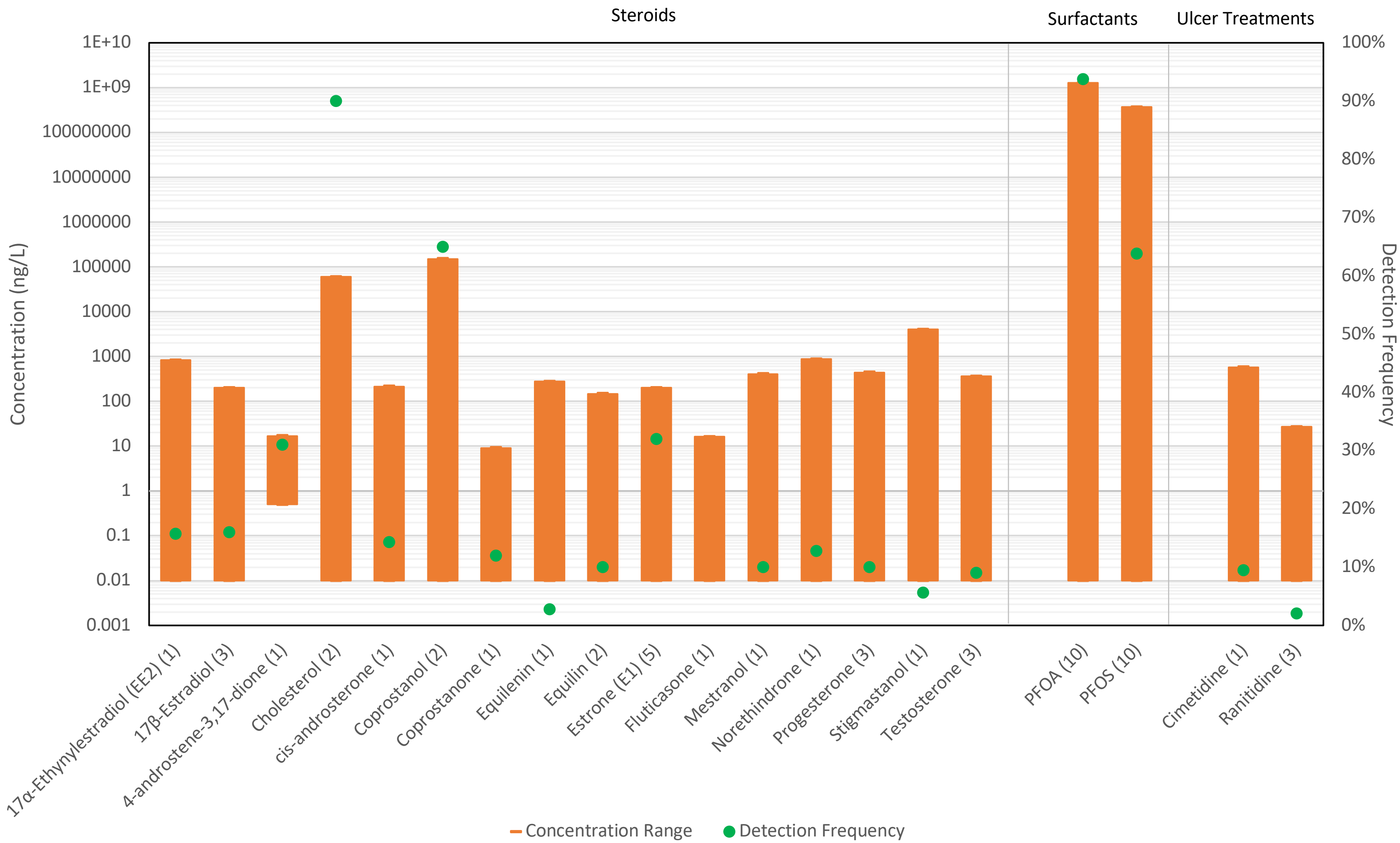

Figure S22. Concentration range of steroids and ulcer treatments detected in surface water (orange bar) and their average detection frequency (green dots). Number of studies on the compounds is indicated in the parentheses (data from Table S6).

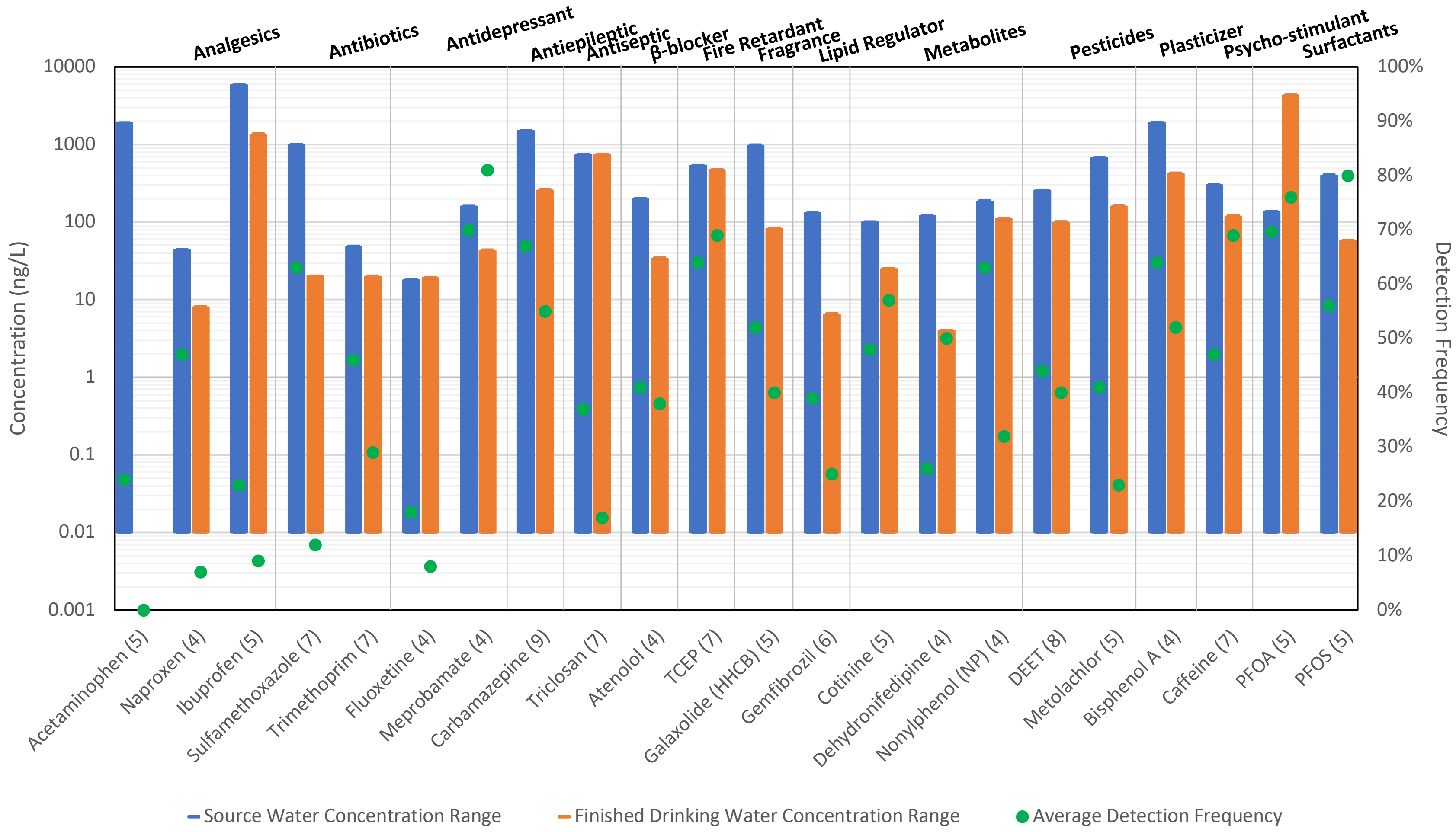

Figure S23. Concentration range of the most studied CECs in WTP source water (blue bar) and finished drinking water (orange bar) and their average detection frequency (green dots). Number of studies on the compounds is indicated in the parentheses (data from Table S7).

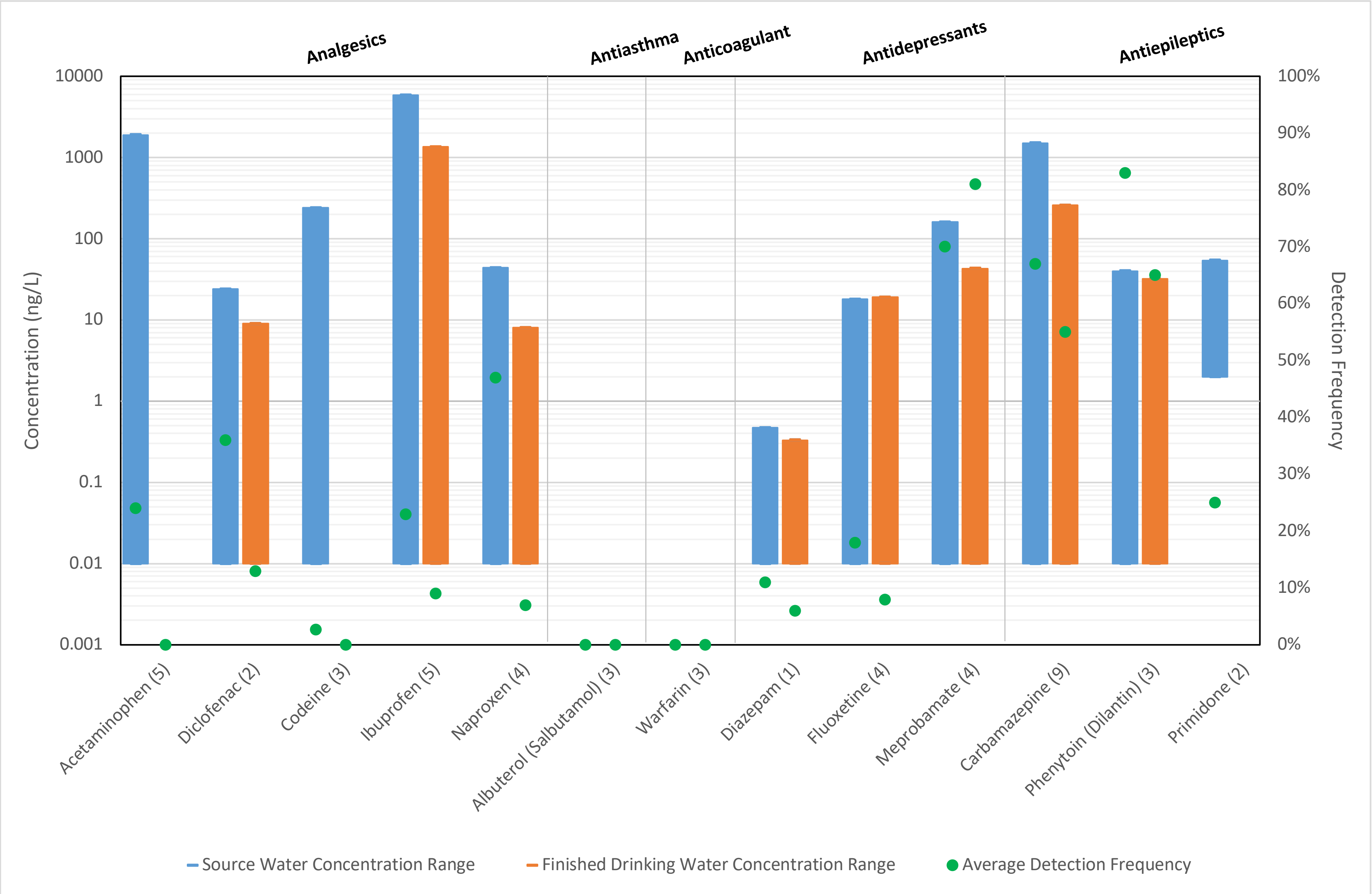

Figure S24. Concentration range of analgesics, antiasthma, anticoagulant, antidepressants, and antiepileptics detected in WTP source water (blue bar) and finished drinking water (orange bar) and their average detection frequency (green dots). Number of studies on the compounds is indicated in the parentheses (data from Table S7).

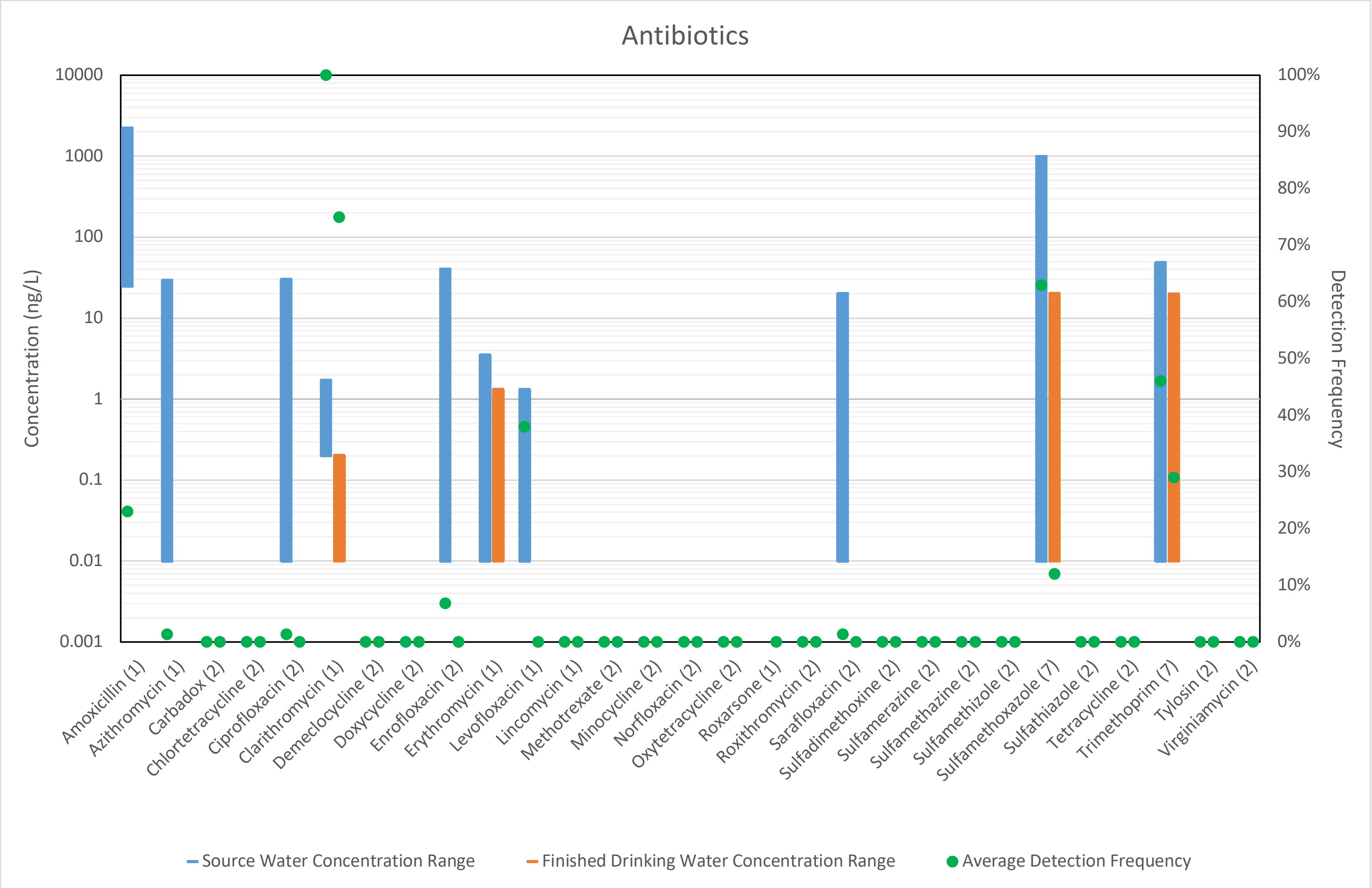

Figure S25. Concentration range of antibiotics detected in WTP source water (blue bar) and finished drinking water (orange bar) and their average detection frequency (green dots). Number of studies on the compounds is indicated in the parentheses (data from Table S7).

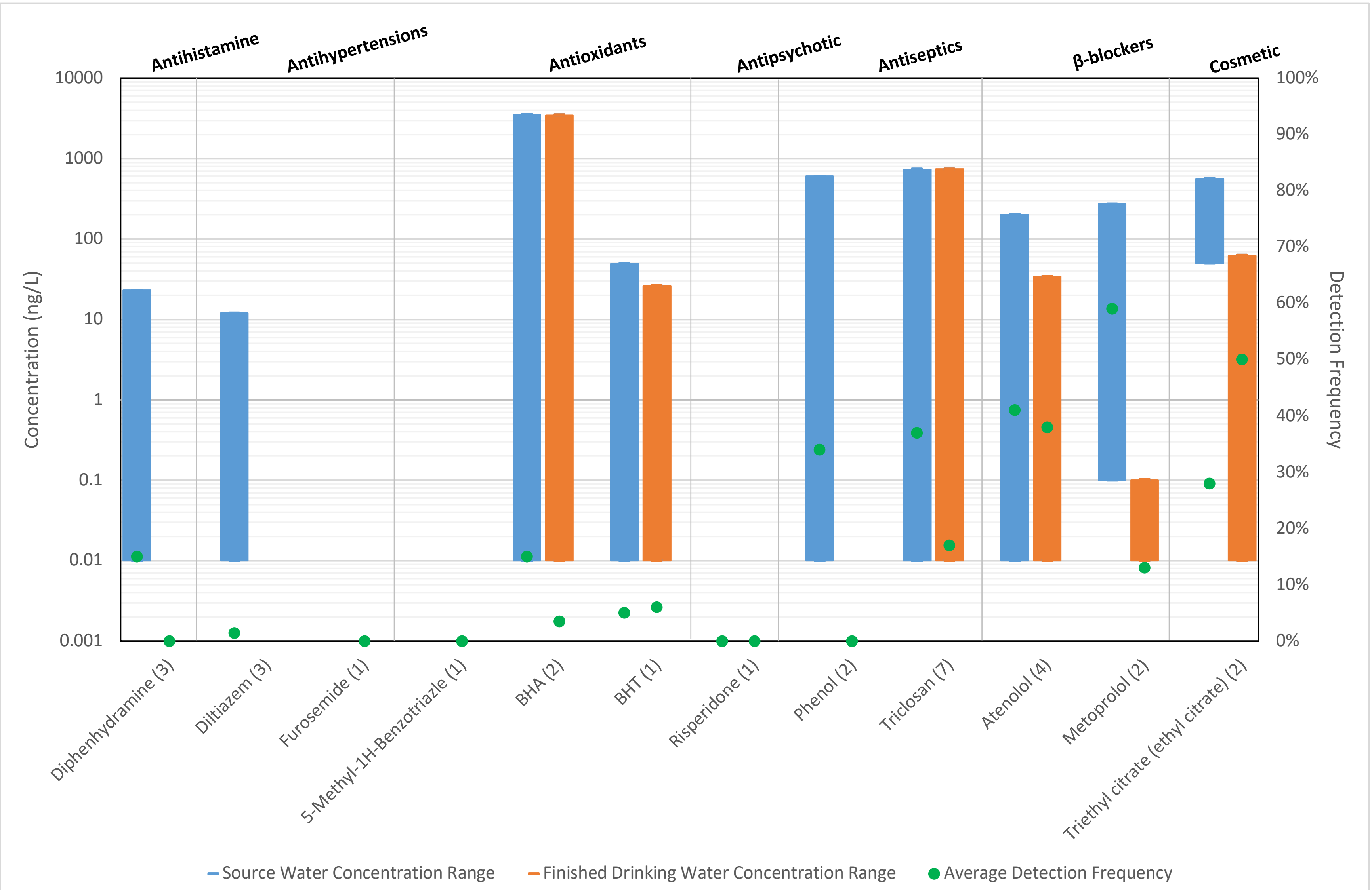

Figure S26. Concentration range of anthihistamine, antihypertensions, antioxidants, antipsychotic, antiseptics, β-blockers, and cosmetic detected in WTP source water (blue bar) and finished drinking water (orange bar) and their average detection frequency (green dots). Number of studies on the compounds is indicated in the parentheses (data from Table S7).

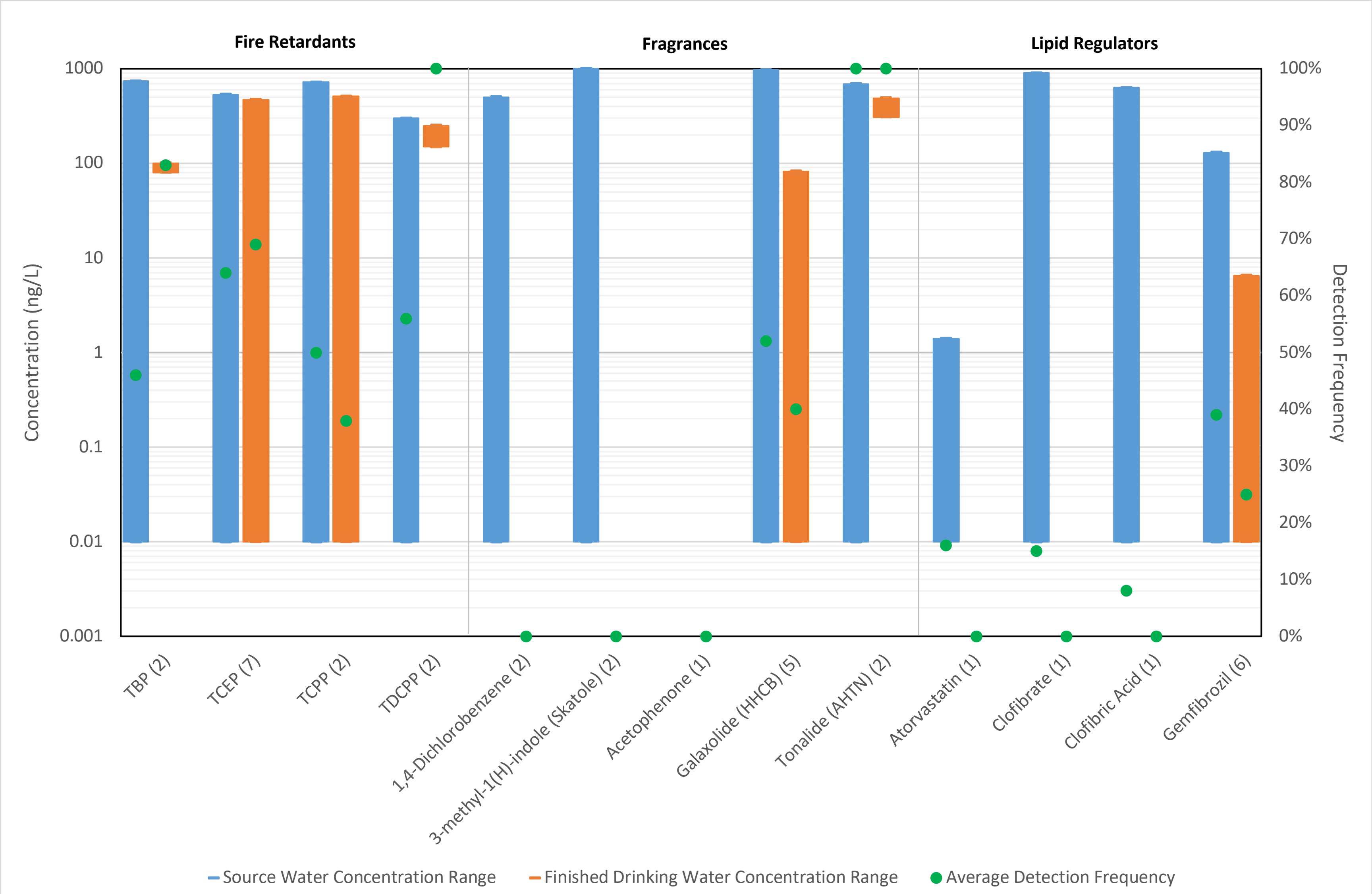

Figure S27. Concentration range of fire retardants, fragrances, and lipid regulators detected in WTP source water (blue bar) and finished drinking water (orange bar) and their average detection frequency (green dots). Number of studies on the compounds is indicated in the parentheses (data from Table S7).

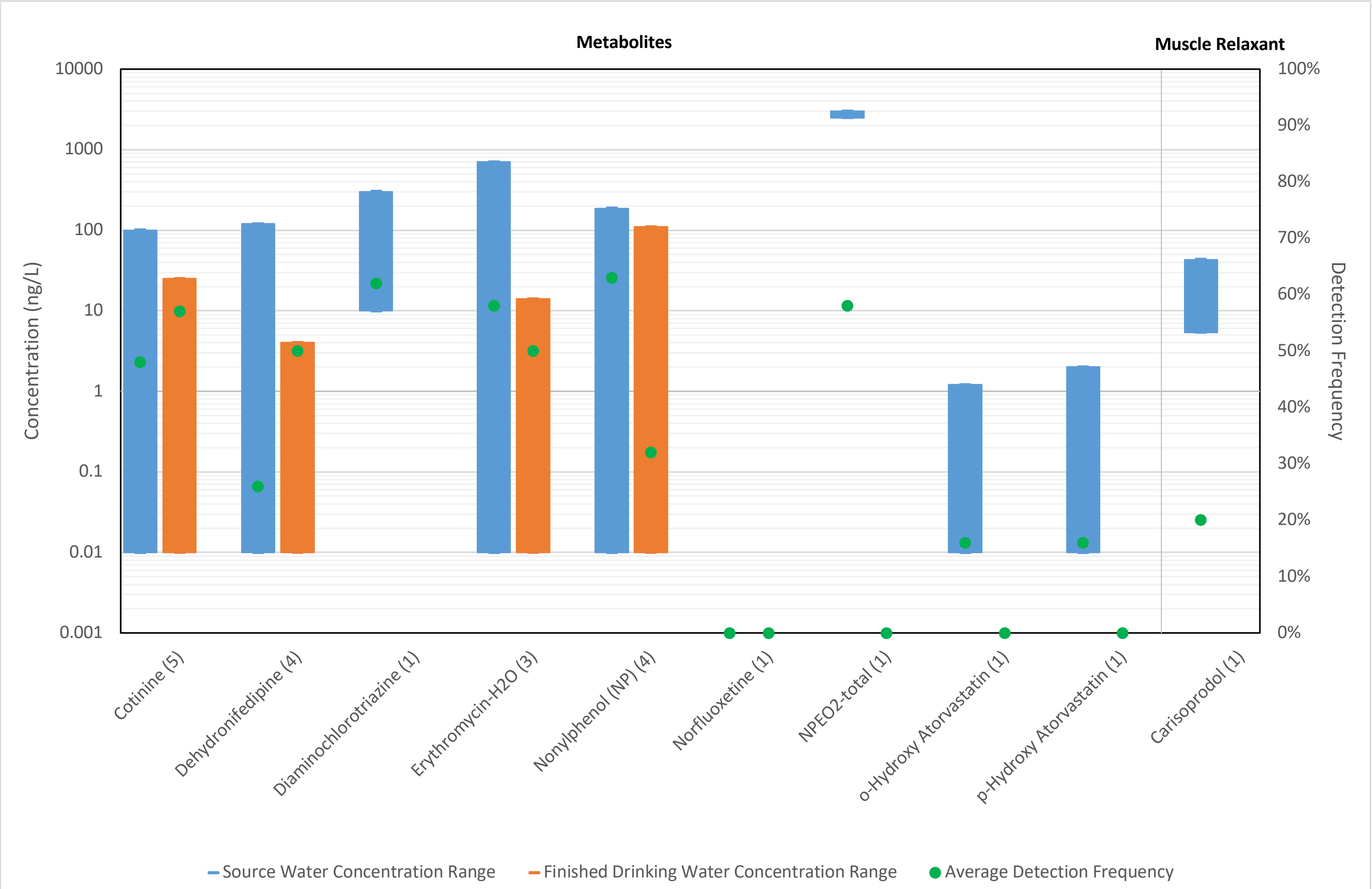

Figure S28. Concentration range of metabolites and muscle relaxant detected in WTP source water (blue bar) and finished drinking water (orange bar) and their average detection frequency (green dots). Number of studies on the compounds is indicated in the parentheses (data from Table S7).

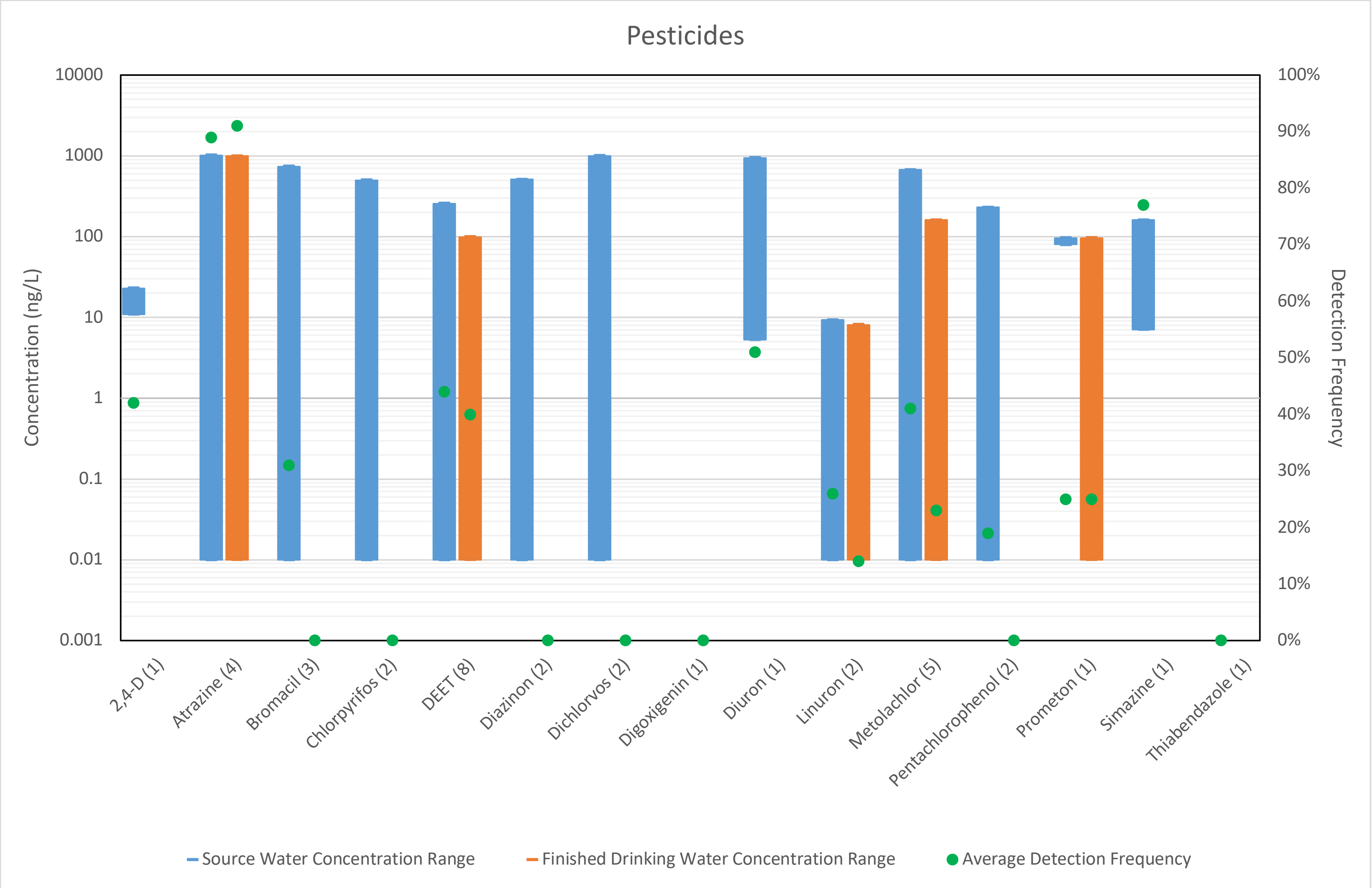

Figure S29. Concentration range of pesticides detected in WTP source water (blue bar) and finished drinking water (orange bar) and their average detection frequency (green dots). Number of studies on the compounds is indicated in the parentheses (data from Table S7).

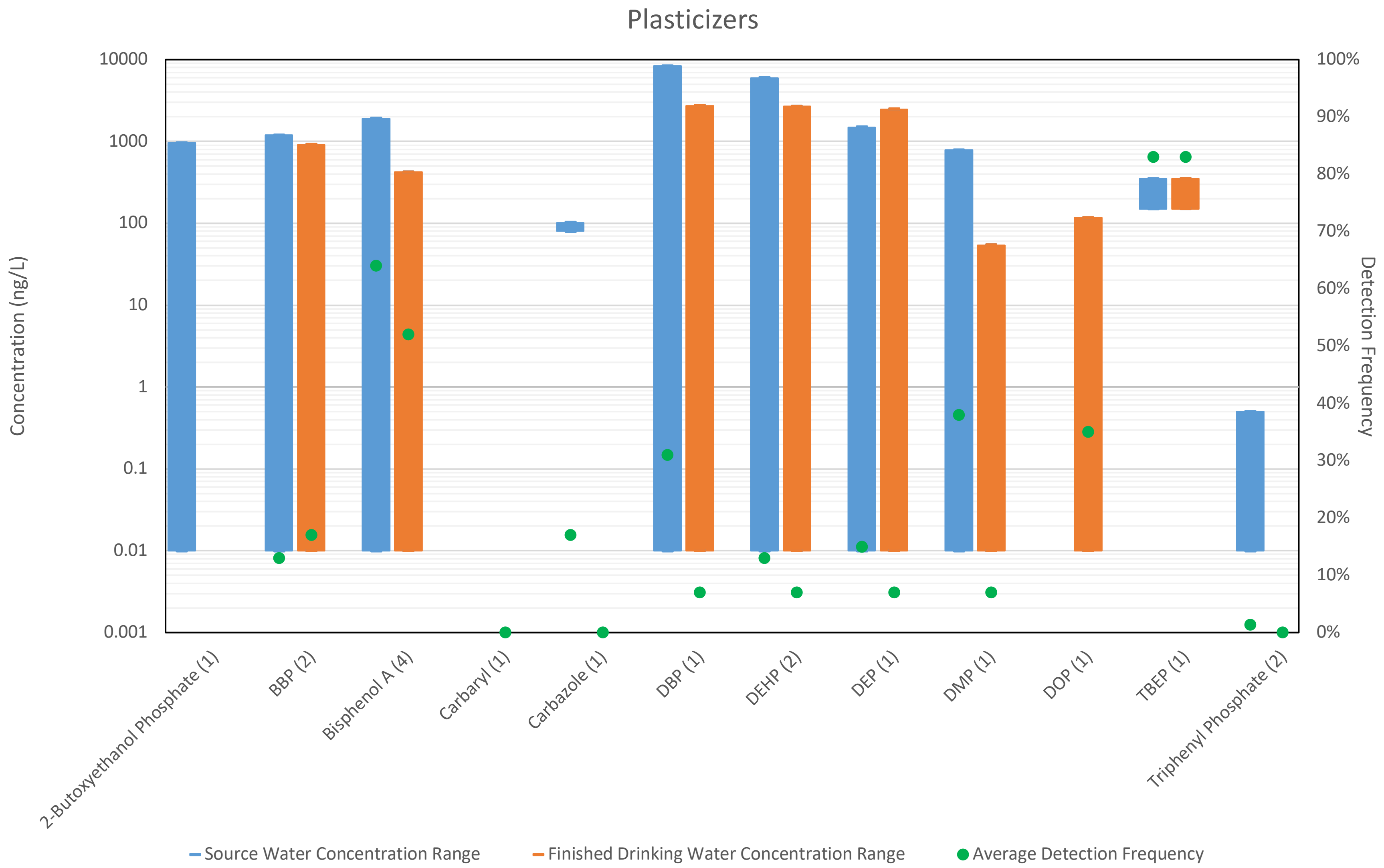

Figure S30. Concentration range of plasticizers detected in WTP source water (blue bar) and finished drinking water (orange bar) and their average detection frequency (green dots). Number of studies on the compounds is indicated in the parentheses (data from Table S7).

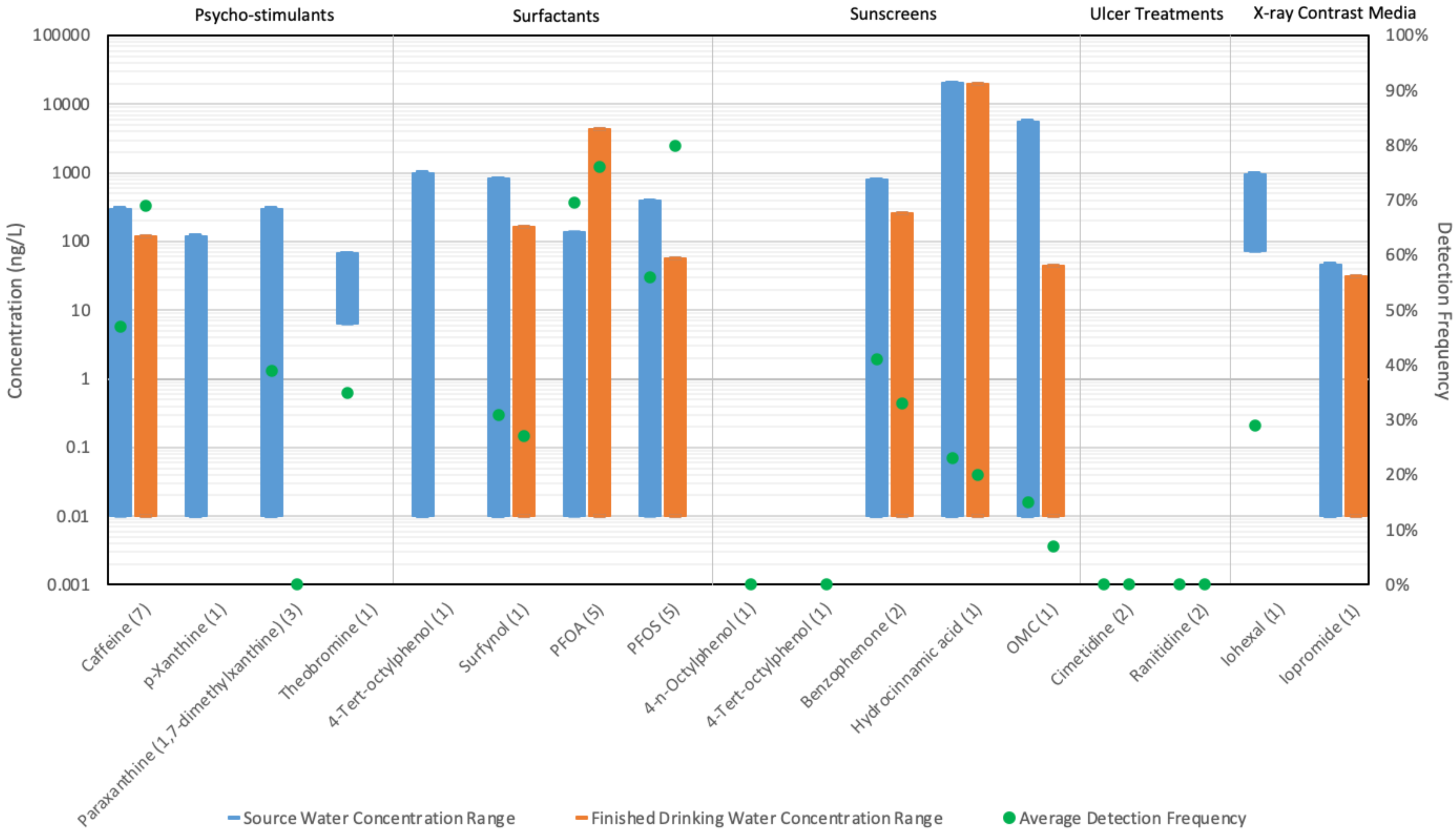

Figure S31. Concentration range of psycho stimulants, surfactants, sunscreens, ulcer treatments, and x-ray contrast media detected in WTP source water (blue bar) and finished drinking water (orange bar) and their average detection frequency (green dots). Number of studies on the compounds is indicated in the parentheses (data from Table S7).

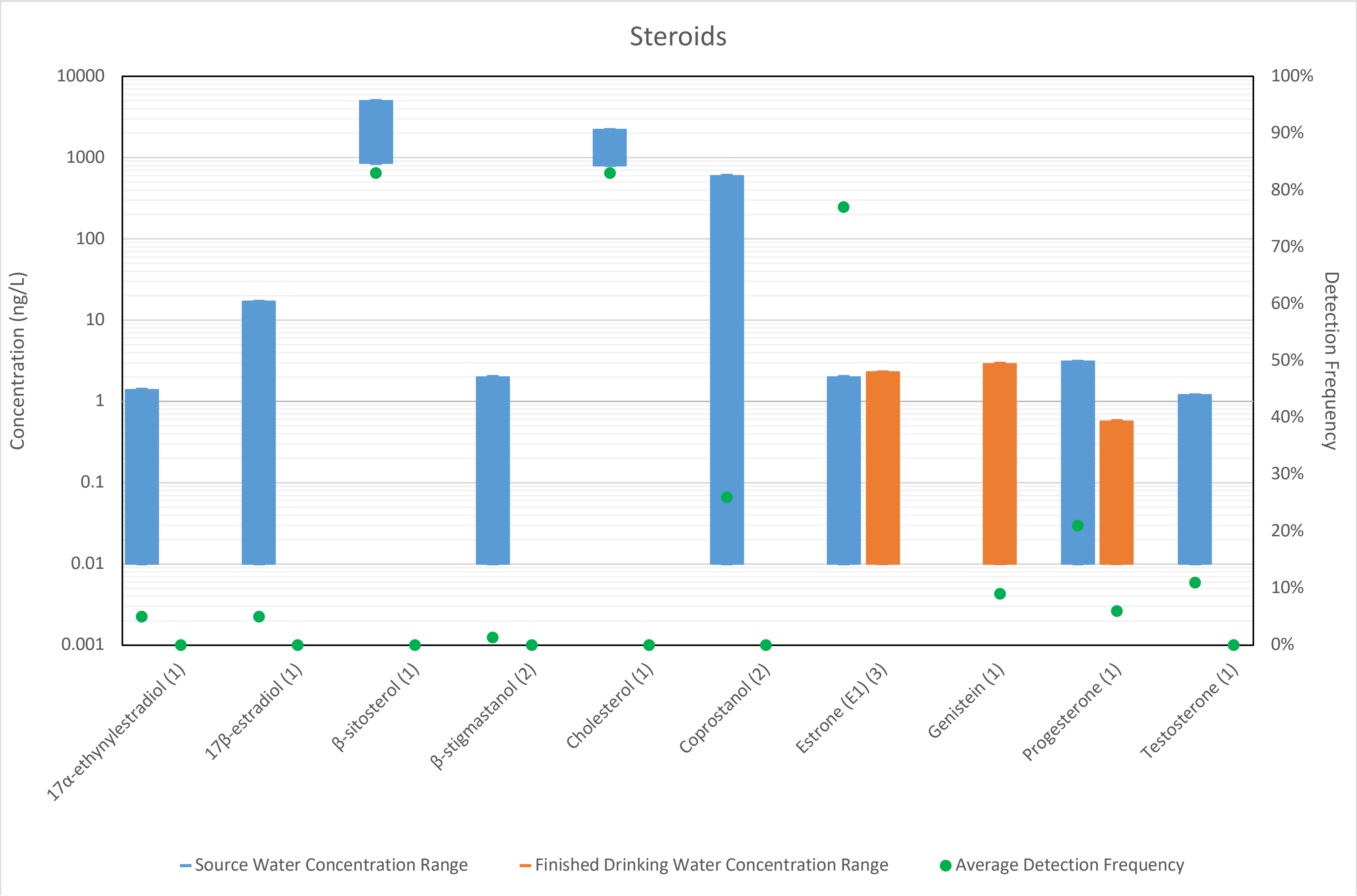

Figure S32. Concentration range of steroids detected in WTP source water (blue bar) and finished drinking water (orange bar) and their average detection frequency (green dots). Number of studies on the compounds is indicated in the parentheses (data from Table S7).

Table S1. Top Therapeutic Classes by Dispensed Prescriptions in the U.S. (IMS Institute for Healthcare Informatics, 2012)

Top Therapeutic Classes by U.S. Dispensed Prescriptions
Total Rx in Millions

RANK		2007	2008	2009	2010	2011
	**Total US Prescription Market**	**3,825**	**3,866**	**3,949**	**3,993**	**4,024**
1	Antidepressants	237	241	247	254	264
2	Lipid Regulators	233	242	254	260	260
3	Narcotic Analgesics	231	239	241	244	238
4	Antidiabetes	165	166	169	172	173
5	Ace Inhibitors (Plain & Combo)	159	163	166	168	164
6	Beta-Blockers (Plain & Combo)	162	164	163	162	161
7	Respiratory Agents	147	147	152	153	153
8	Anti-Ulcerants	134	139	146	147	150
9	Diuretics	137	135	132	131	128
10	Anti-Epileptics	102	110	116	122	128
11	Tranquillizers	98	101	104	108	111
12	Thyroid Preparations	103	104	105	107	110
13	Calcium Antagonists (Plain &Combo)	87	90	93	96	98
14	Antirheumatic Non-Steroid	90	91	92	93	97
15	Hormonal Contraceptives	94	94	93	91	90
16	Angiotensin II	83	86	85	84	86
17	Broad Spectrum Penicillins	77	74	77	76	77
18	Macrolides & Similar Type Antibiotics	63	66	69	67	69
19	Hypnotics & Sedatives	58	60	63	63	63
20	Vitamins and Minerals	60	59	58	58	60

Table S2. Top Therapeutic Classes by U.S. Sales (IMS Institute for Healthcare Informatics, 2012)

Top Therapeutic Classes by U.S. Sales
US Dollar in Billions

RANK		2007	2008	2009	2010	2011
	**Total US Prescription Market**	**280.5**	**285.7**	**300.7**	**308.6**	**319.9**
1	Oncologics	18.1	19.7	21.5	22.3	23.2
2	Respiratory Agents	15.1	16.0	18.1	19.3	21.0
3	Lipid Regulators	19.4	18.1	18.6	18.8	20.1
4	Antidiabetics	12.2	13.6	15.8	17.7	19.6
5	Antipsychotics	12.8	14.3	14.7	16.2	18.2
6	Autoimmune Diseases	7.6	8.6	9.7	10.6	12.0
7	Antidepressant	11.7	11.7	11.5	11.6	11.0
8	HIV Antivirals	6.2	7.1	8.2	9.3	10.3
9	Anti-Ulcerants	14.6	14.2	14.1	11.9	10.1
10	Narcotic Analgesics	6.7	7.3	8.0	8.4	8.3
11	ADHD	4.0	4.7	5.8	6.7	7.9
12	Platelet Aggregation Inhibitors	5.0	5.7	6.5	7.1	7.8
13	Angiotensin II	6.5	7.6	8.6	8.7	7.6
14	Multiple Sclerosis	3.4	4.1	5.0	5.8	7.1
15	Vaccines (Pure, Comb, Other)	5.9	5.0	4.7	5.7	6.3
16	Anti-Epileptics	10.0	11.1	6.9	5.6	5.9
17	Hormonal Contraceptives	4.1	4.5	4.7	4.8	5.2
18	Erythropoietins	8.4	6.9	6.3	6.1	5.1
19	Immunostimulating Agents	4.1	4.1	4.1	4.2	4.5
20	Antivirals, Excl. Anti-HIV	3.6	3.9	4.8	3.2	3.7

Table S3. Most commonly used active ingredients in conventional pesticides for the agricultural market sector, 1987 to 2007 estimates (ranked by range in millions of pounds of active ingredient) (adapted from Kiely et al., 2004; Grube et al., 2011)

Active Ingredient	Type	2007		2005		2003		2001		1999		1997		1987	
		Rank	Range	Rank	Range	Rank	Range	Rank	Range	Rank	Range	Rank	Range	Rank	Range
Glyphosate	H	1	180-185	1	155-160	1	128-133	1	85-90	2	67-73	5	34-38	17	6-8
Atrazine	H	2	73-78	2	70-75	2	75-80	2	74-80	1	74-80	1	75-82	1	71-76
Metam Sodium	Fum	3	50-55	3	39-44	3	45-50	3	57-62	3	60-64	3	53-58	15	5-8
Metolachlor-s	H	4	30-35	5	27-32	6	28-33	9	20-24	12	16-19	NA	NA	NA	NA
Acetochlor	H	5	28-33	6	26-31	5	30-35	4	30-35	4	30-35	7	31-36	NA	NA
Dichloropropene	Fum	6	27-32	4	30-35	7	20-24	8	20-25	11	17-20	6	32-37	4	30-35
2,4-D	H	7	25-29	7	24-28	4	30-35	5	28-33	6	28-33	8	29-33	5	29-33
Methyl Bromide	Fum	8	11-15	8	12-16	8	13-17	7	20-25	5	28-33	4	38-45	NA	NA
Chloropicrin	Fum	9	9-11	10	9-12	9	9-12	18	5-9	NA	NA	NA	NA	NA	NA
Pendimethalin	H	10	7-9	9	9-12	10	9-12	11	15-19	10	18-22	9	4-28	10	10-13
Ethephon	PGR	11	7-9	11	8-10	15	6-7	21	5-8	24	5-6	NA	NA	NA	NA
Chlorothalonil	F	12	7-9	13	7-9	14	7-9	13	8-11	13	8-11	15	7-10	19	5-7
Metam Potassium	Fum	13	7-9	20	4-6	20	4-6	NA	1-2	NA	NA	NA	NA	NA	NA
Chlorpyrifos	I	14	7-9	15	6-8	13	7-9	15	7-10	16	8-10	14	9-13	14	6-9
Copper Hydroxide	F	15	6-8	12	8-10	12	7-9	14	8-10	15	8-10	13	10-13	19	5-7
Simazine	H	16	5-7	17	5-7	17	6-7	23	5-7	NA	NA	NA	NA	NA	NA
Trifluralin	H	17	5-7	14	7-9	11	8-10	12	12-16	9	18-23	10	1-25	6	25-30
Propanil	H	18	4-6	18	4-6	18	5-7	17	6-9	18	7-10	22	6-8	13	7-10
Mancozeb	F	19	4-6	16	6-8	16	6-7	20	6-8	21	6-8	17	7-10	21	4-6
Aldicarb	I	20	3-4	21	3-5	25	4-6	NA	3-5	NA	NA	NA	NA	NA	NA
Acephate	I	21	2-4	24	2-4	NA	1-3	NA	1-3	NA	NA	NA	NA	NA	NA
Diuron	H	22	2-4	19	4-6	21	2-6	NA	3-6	NA	NA	NA	NA	NA	NA
MCPA	H	23	2-4	NA	2-4	24	4-6	NA	3-5	NA	NA	NA	NA	NA	NA
Paraquat	H	24	2-4	25	2-4	NA	3-4	NA	3-5	NA	NA	NA	NA	NA	NA
Dimethenamid	H	25	2-4	NA	2-4	23	4-6	19	6-8	20	6-8	20	6-9	NA	NA

H – Herbicide; I – Insecticide; F – Fungicide; Fum – Fumigant; PGR – Plant Growth Regulator; NA – an estimated is not available; 2,4-D – 2,4-Dichlorophenoxyacetic acid; MCPA – 4-chloro-2-methylphenoxy acetic acid.

Table S4. Total excretion of the CECs reported in literature

Class	Compound	Total Excretion (%)[1],[2],[3]
Analgesic	Acetaminophen	83
	Diclofenac	16-51
	Ibuprofen	5-100
Antibiotic	Sulfamethoxazole	20-98
	Trimethoprim	40-69
Antidepressant	Diazepam	85
	Fluoxetine	71
Antiepileptic	Carbamazepine	21-63
β-Blocker	Atenolol	69-96
	Metoprolol	11-93
	Propranolol	100
Lipid Regulator	Gemfibrozil	5-50

[1] Lienert et al., 2007; [2] Luo et al., 2014; [3] ter Laak, et al., 2010.

Table S5. Occurrence of CECs that have been reported in WWTP influent and effluent in the United States

Groups	Compounds	WWTP Influent (ng/L)			Freq.	WWTP Effluent (ng/L)			Freq.	Ref.
		Minimum	Mean	Maximum	%	Minimum	Mean	Maximum	%	
Analgesics	Acetaminophen (Paracetamol)		960							[2]
			960				ND			[3]
								1060	50	[5]
						ND		260		[6]
			>2,000				130			[11]
							79	1,500	14	[12]
						<50				[15]
			61683		100		98		100	[16]
		370	41700	218000		ND	85.5	210		[17]
			39500				ND			[19]
	Aspirin	170	440	930		ND	29.4	70		[17]
	Brompheniramine		40				ND			[11]
	Codeine							730	72.5	[5]
			15				3			[19]
	Diclofenac		116				<0.5			[2]
			110				90			[3]
							165			[8]
		ND		14		8.3		177		[9]
						<10		440		[13]
								<10		[15]
		86	277	580		ND	12	120		[17]
			36				35			[19]
	Hydrocodone						22	92	44	[12]
	Ibuprofen		1900				250			[3]
						5		425		[4]
						ND		88		[6]
							13			[8]
							460	4200	46	[12]
						<10				[15]
		8600	22300	56500		13	55.8	92		[17]
			12053				ND			[19]
	Indomethacin		54				11			[19]
	Mefenamic Acid		3				6			[19]
	Naproxen		22500				<1.0			[2]
			3200				380			[3]

Table S5. Occurrence of CECs that have been reported in WWTP influent and effluent in the United States (continued).

Groups	Compounds	WWTP Influent (ng/L)			Freq.	WWTP Effluent (ng/L)			Freq.	Ref.
		Minimum	Mean	Maximum	%	Minimum	Mean	Maximum	%	
Analgesics	Naproxen					26		172		[4]
						81		106		[7]
							72			[8]
						<20		3680		[13]
		9300	35700	210000		ND	30.3	150		[17]
			13418				5			[19]
	Ketoprofen		1200				280			[3]
						ND		29		[4]
		150	563	1300		ND	29	65		[17]
			97				ND			[19]
	Oxycodone					53		150		[6]
							53	310	60	[12]
	Propoxyphene					ND		65		[6]
							17	34	25	[12]
	Salicylic Acid	434		8,036		ND		25		[9]
						40		1680		[13]
Antagonists	Benztropine					ND		0.9		[6]
							ND	ND	0	[12]
Antiasthmas	Albuterol (Salbutamol)					ND		60		[6]
			780				110			[11]
							14	35	54	[12]
			12				9			[19]
	Theophylline						<88	<88	8	[12]
Antibiotics	Azithromycin						690			[10]
			1083				ND			[19]
	Chlortetracycline	ND		16		ND		ND		[9]
			178		33.3		ND		0	[16]
	Ciprofloxacin	12		377		8.8		110		[9]
							67	260	61	[12]
						<10		16		[15]
			2747				67			[19]
	Clarithromycin	ND		106		ND		611		[9]
			122				103			[19]
	Clindamycin	6.8		13		15		33		[9]
	Demeclocycline		ND		0		ND		0	[16]

Table S5. Occurrence of CECs that have been reported in WWTP influent and effluent in the United States (continued)

Groups	Compounds	WWTP Influent (ng/L)			Freq.	WWTP Effluent (ng/L)			Freq.	Ref.
		Minimum	Mean	Maximum	%	Minimum	Mean	Maximum	%	
Antibiotics	Doxycycline		738		100		370		33.3	[16]
								ND	0	[5]
	Erythromycin							610	52.5	[5]
						<10	2	15		[15]
			ND		0		ND		0	[16]
	Florfenicol						ND	ND	0	[12]
	Levofloxacin					<10		10		[15]
	Lincomycin						ND	ND	0	[12]
						<10				[15]
			58		83.3		35		66.7	[16]
	Metronidazole		169				229			[19]
	Ofloxacin						160	660	90	[12]
	Oxytetracycline		29		100		17		33.3	[16]
	Sulfachloropyridazine						2			[10]
	Sulfadiazine		37		33.3		28		16.7	[16]
			65				30			[19]
	Sulfadimethoxine	ND		2.6		ND		1.9		[9]
							67			[10]
							ND	ND	0	[12]
	Sulfamerazine		ND		0		ND		0	[16]
	Sulfamethazine	ND		27		ND		ND		[9]
							5			[10]
							12	87	2	[12]
			26		16.7		ND		0	[16]
			ND				26			[19]
	Sulfamethazole						1			[10]
	Sulfamethoxazole		2060				5			[2]
								763	72.5	[5]
						98		2200		[6]
							443			[8]
		14		261		79		472		[9]
							141			[10]
							330	2900	90	[12]
		846	1786	3905	100	ND	304	1328	89	[14]
						35	80	140		[15]
			1566		100		178		100	[16]

Table S5. Occurrence of CECs that have been reported in WWTP influent and effluent in the United States (continued)

Groups	Compounds	WWTP Influent (ng/L)			Freq.	WWTP Effluent (ng/L)			Freq.	Ref.
		Minimum	Mean	Maximum	%	Minimum	Mean	Maximum	%	
Antibiotics	Sulfamethoxazole		1216				570			[19]
	Sulfisoxazole (Sulfafurazole)	ND		22		ND		12		[9]
	Tetracycline	ND		39		ND		31		[9]
						<10				[9]
			310		83.3		ND		0	[16]
			2512				111			[19]
	Trimethoprim		1140				<0.5			[2]
								414	60	[5]
						39		140		[6]
							620			[8]
							90	370	86	[12]
						<10				[15]
			220				18			[19]
	Tylosin		ND		0		ND		0	[16]
Anti-cancer	Tamoxifen		11				2			[19]
Anticoagulants	Warfarin					ND		50		[6]
							ND	ND	0	[12]
Antidepressants	Amitriptyline					25		86		[6]
			>2,000				1,490			[11]
							11	110	40	[12]
	Alprazolam					10		18		[6]
							10	31	30	[12]
		3.09	6.24	12.6	100	5.01	6.2	8.21	100	[20]
	Bupropion					<10		4300		[18]
		17.1	110	231	100	35.9	118	310	100	[20]
	Citalopram					<10		520		[18]
		77.7	133	170	100	205	280	414	100	[20]
	Diazepam		<2.5				3.7			[2]
							3			[8]
			40				40			[11]
		2.29	3.38	4.57	100	<0.05	1.62	3.5	71	[20]
	Fluoxetine		17				25			[2]
							44			[8]
							8.7	31	37.5	[12]
						<10		76		[18]

Table S5. Occurrence of CECs that have been reported in WWTP influent and effluent in the United States (continued)

Groups	Compounds	WWTP Influent (ng/L)			Freq.	WWTP Effluent (ng/L)			Freq.	Ref.
		Minimum	Mean	Maximum	%	Minimum	Mean	Maximum	%	
Antidepressants	Fluoxetine		144				27			[19]
						40		73		[6]
	Lorazepam		7				3			[19]
		9.14	20.3	33	100	37.3	64.2	85.5	100	[20]
	Meprobamate		1440				1270			[2]
							603			[8]
	Oxazepam	2.3	8.43	16.9	100	7.09	9.87	14.4	100	[20]
	Paroxetine					2.5		13		[6]
							ND	ND	0	[12]
			270				16			[19]
	Promethazine					0.4		16		[6]
							ND	ND	0	[12]
	Sertraline					57		87		[6]
							21	71	64	[12]
		46.7	80.8	114	100	27.9	62.8	88.3	100	[20]
	Venlafaxine					<10		5500		[18]
		169	415	609	100	389	480	553	100	[20]
Antidiabetics	Glibenclamide		12				7			[19]
	Glipizide					ND		30		[6]
	Glyburide					ND		120		[6]
Antiepileptics	Carbamazepine		232				187			[2]
						ND		59		[4]
								270	82.5	[5]
						70		800		[6]
							180			[8]
		25		51		34		111		[9]
							120			[10]
			600				140			[11]
							97	240	96	[12]
		66	137	348	100	66	156	270	100	[14]
						<10		12		[15]
			110		100		155		100	[16]
		34	115	350		ND	21.3	62		[17]
						<10		1500		[18]
			386				223			[19]
		61	145	347	100	150	310	731	100	[20]

Table S5. Occurrence of CECs that have been reported in WWTP influent and effluent in the United States (continued)

Groups	Compounds	WWTP Influent (ng/L)			Freq.	WWTP Effluent (ng/L)			Freq.	Ref.
		Minimum	Mean	Maximum	%	Minimum	Mean	Maximum	%	
Antiepileptics	Gabapentin		100				ND			[3]
						<10		3100		[18]
	Lamotrigine					<10		1200		[18]
	Oxcarbazepine					<10		480		[18]
	Phenytoin (Dilantin)		402				287			[2]
			450				250			[3]
							600			[8]
	Primidone						183			[8]
			130				100			[11]
						25	46	120		[15]
	Valproic Acid		140				ND			[3]
Antihistamine	Diphenhydramine							387	55	[5]
							589			[10]
		286	462	615	100	<0.05	194	704	86	[20]
	Famotidine		635				145			[19]
	Loratadine		26				6			[19]
Antihypertensions	Amlodipine						6.9	18	22	[12]
	Clonidine						ND	ND	0	[12]
	Clopidogrel	7.4	35.5	92.8	100	13.9	21.8	30.1	100	[20]
	Desmethyldiltiazem					65		110		[6]
							24	100	18	[12]
	Diltiazem							146	67.5	[5]
						28		200		[6]
		ND		69		ND		107		[9]
							85	340	84	[12]
		15.3	105	215	100	179	194	218	100	[20]
	Enalapril		35				0.85			[2]
							4.6	38	27	[12]
			83				ND			[19]
	Enalaprilat						14	150	10	[12]
	Furosemide					180		930		[6]
							280	810	90	[12]
			1830				497			[19]
	Hydrochlorothiazide					1300		2950		[6]
							1100	2800	100	[12]
			2325				1453			[19]

Table S5. Occurrence of CECs that have been reported in WWTP influent and effluent in the United States (continued)

Groups	Compounds	WWTP Influent (ng/L)			Freq.	WWTP Effluent (ng/L)			Freq.	Ref.
		Minimum	Mean	Maximum	%	Minimum	Mean	Maximum	%	
Antihypertensions	Lisinopril						180	3300	47	[12]
			2693				ND			[19]
	Triamterene					130		440		[6]
							37	170	70	[12]
	Valsartan					60		1500		[6]
							1600	5300	98	[12]
	Verapamil					14		190		[6]
							26	97	80	[12]
		5.34	18.5	42.8	100	28.8	49.2	63.2	100	[20]
Antineoplastics	5-Fluorouracil		ND				ND			[3]
Antioxidents	5-Methyl-1H-Benzotriazle							1700	45	[5]
Antipsychotics	Aripiprazole	ND	5.58	22.4	57	ND	10.3	20.9	86	[20]
	Quetiapine	11	24.4	43.6	100	ND	0.98	3.43	57	[20]
	Risperidone		<2.5				<0.5			[2]
Antiseptics	4-Chloro-m-cresol		600				ND			[3]
	Biosol		250				ND			[3]
	Biphenylol		900				100			[3]
	Chlorophene		750				200			[3]
	Chloroxylenol (PCMX)		400				80			[3]
	Ciclopirox		1,410				321			[11]
	Terbinafine		510				120			[11]
	Triclosan		1280				<2.0			[2]
								1600	62.5	[5]
						10		21		[7]
							203			[8]
						<10				[10]
		180	2300	4400		ND	48.4	160		[17]
			800				250			[3]
Barbiturates	Butalbital		62				50			[19]
	Pentobarbital		92				67			[19]
	Phenobarbital		70				ND			[3]
			101				118			[19]
	Secobarbital		ND				ND			[3]
β-blockers	Atenolol		3060				879			[2]

Table S5. Occurrence of CECs that have been reported in WWTP influent and effluent in the United States (continued)

Groups	Compounds	WWTP Influent (ng/L)			Freq.	WWTP Effluent (ng/L)			Freq.	Ref.
		Minimum	Mean	Maximum	%	Minimum	Mean	Maximum	%	
β-blockers	Atenolol					120		960		[6]
							1267			[8]
							940	3,000	96	[12]
			2256				208			[19]
		377	1220	2000	100	382	594	754	100	[20]
	Metoprolol					ND	74	1,200	88	[1]
						150		650		[6]
			770				130			[11]
							410	660	98	[12]
			1518				908			[19]
	Nadolol					ND	52	360	71	[1]
			57				ND			[19]
	Propranolol					26	117	1,900	100	[1]
						32		77		[6]
							33	260	88	[12]
			95				62			[19]
		3.04	24.5	53.8	100	29.8	82.2	225	100	[20]
	Sotalol		603				246			[19]
Cosmetics	Triethyl citrate (ethyl citrate)							520	72.5	[5]
Fire Retardants	TBP							470	70	[5]
	TCEP							480	75	[5]
							543			[8]
	TCPP							480	77.5	[5]
							3750			[8]
Fragrances	1,4-Dichlorobenzene							910	27.5	[5]
	Galaxolide (HHCB)							530	57.5	[5]
		240	1376	4020	100	145	465	1104	100	[14]
	Musk Ketone						48			[8]
	Tonalide (AHTN)							2600	80	[5]
Lipid Regulators	Atorvastatin		201				<0.5			[2]
						ND		42		[6]
							58			[8]
						<38	<38	<38	8	[12]
			939				ND			[19]
	Bezafibrate		4				ND			[19]

Table S5. Occurrence of CECs that have been reported in WWTP influent and effluent in the United States (continued)

Groups	Compounds	WWTP Influent (ng/L)			Freq.	WWTP Effluent (ng/L)			Freq.	Ref.
		Minimum	Mean	Maximum	%	Minimum	Mean	Maximum	%	
Lipid Regulators	Fenofibrate		250				ND			[11]
			317				3			[19]
	Gemfibrozil		4770				9.0			[2]
			410				130			[3]
						ND		158		[4]
						47		1220		[6]
							890			[8]
		182		451		ND		42		[9]
							420	2300	76	[12]
						63		3830		[13]
		315	934	1757	100	6	41	207	100	[14]
		1090	467	8500		ND	151	650		[17]
			1220				42			[19]
	Mevastatin		507				ND			[19]
	Pravastatin		783				ND			[19]
	Simvastatin		<2.5				<0.5			[2]
							<41	<41	24	[12]
	Simvastatin Hydroxy Acid		10				<0.5			[2]
Metabolites	10-OH-CBZ					<10		1900		[18]
	2-Diphenhydramine acetic acid	<0.05	3.7	5.52	86	<0.05	4.5	12.9	29	[20]
	2-Hydroxy-Ibuprofen					ND		200		[6]
	4-Nonylphenol Diethoxylate							38000	62.5	[5]
	4-Nonylphenol Monoethoxylate							18000	62.5	[5]
	4-Octylphenol Diethoxylate							360	32.5	[5]
	10-Hydroxy-Amitriptyline					ND		64		[6]
						<5	<5	<5	12	[12]
	α-Hydroxy Alprazolam	5.86	21.4	28.5	100	7.25	9.2	12.4	100	[20]
	Clopidogrel Carboxylic Acid	43.5	160	278	100	166	194	249	100	[20]

Table S5. Occurrence of CECs that have been reported in WWTP influent and effluent in the United States (continued)

Groups	Compounds	WWTP Influent (ng/L)			Freq.	WWTP Effluent (ng/L)			Freq.	Ref.
		Minimum	Mean	Maximum	%	Minimum	Mean	Maximum	%	
Metabolites	Cotinine							1030	92.5	[5]
		41		1,581		ND		ND		[9]
							147			[10]
		130		2,700						[21]
	Dehydroaripiprazole	ND	ND	ND	0	ND	ND	ND	0	[20]
	Dehydronifedipine							22	57.5	[5]
	Desacetyl Diltiazem	125	483	1090	100	163	294	368	100	[20]
	Desmethylsertraline (Norsertraline)						9.9	24	18	[12]
		23.7	71.1	137	100	ND	54.4	78.6	86	[20]
	DMV					<10		10000		[18]
	DiOH-CBZ					<10		400		[18]
	Glu-LMG					<100		455		[18]
	Hydroxybupropion					<10		2000		[18]
	N-desmethylcitalopram					<10		200		[18]
		ND	55.4	126	71	35.9	118	310	100	[20]
	Nonylphenol (NP)		130				103			[11]
						<50		3610		[13]
		220	510	870		ND	9	50		[17]
	Nordiazepam	1.68	5.3	9.73	100	3.05	4.53	6.56	100	[20]
	Norfluoxetine		9.9				3.9			[2]
						1.2		1.2		[6]
							7.7	15	17	[12]
	Norquetiapine	39.5	71	159	100	25	74.3	131	100	[20]
	Norverapamil					42		71		[6]
							5.8	20	52	[12]
		4.2	14.8	33.5	100	2.6	20.3	39.9	100	[20]
	NPEC		5465				25159			[19]
	o-Hydroxy Atorvastatin		196				<1.0			[2]
	p-Hydroxy Atorvastatin		280				<1.0			[2]
	PEG		39237				1606			[9]
	Threo/erythro-hydrobupropion					<10		5700		[18]

Table S5. Occurrence of CECs that have been reported in WWTP influent and effluent in the United States (continued)

Groups	Compounds	WWTP Influent (ng/L)			Freq.	WWTP Effluent (ng/L)			Freq.	Ref.
		Minimum	Mean	Maximum	%	Minimum	Mean	Maximum	%	
Muscle Relaxants	Carisoprodol		410				141			[11]
Pesticides	Atrazine		<2.5				0.81			[2]
							3			[8]
	DEET							2100	70	[5]
							106			[8]
							774			[10]
			450				120			[11]
						<10	18	30		[15]
	Diazinon							150	47.5	[5]
	Linuron		<5.0				0.73			[2]
	Metolachlor							97	37.5	[5]
	Pentachlorophenol							86	35	[5]
	Phenol							1800	40	[5]
	Thiabendazole						33			[10]
Plasticizers	2-Butoxyethanol Phosphate							12000	70	[5]
	Bisphenol A (BPA)		320				<2.0			[2]
							43			[8]
						<10		697		[13]
		60	296	600		ND	12.6	81		[17]
	n-Butylbenzene-sulfonamide		694				120			[11]
	Triphenyl Phosphate							180	37.5	[5]
Psycho-stimulants	Amphetamine					0.2		0.2		[6]
							3.5	40	10	[12]
	Caffeine							7990	70	[5]
							43			[8]
		2,449		4,866		3.9		23.1		[9]
							535			[10]
			>2,000				60			[11]
						<10	17	50		[15]
			41204		100		76		100	[16]
		11500		120000						[21]
	Methamphetamine						350			[10]
	Paraxanthine (1,7-dimethylxanthine)							8550	35	[5]

Table S5. Occurrence of CECs that have been reported in WWTP influent and effluent in the United States (continued)

Groups	Compounds	WWTP Influent (ng/L)			Freq.	WWTP Effluent (ng/L)			Freq.	Ref.
		Minimum	Mean	Maximum	%	Minimum	Mean	Maximum	%	
Psycho-stimulants	Paraxanthine (1,7-dimethylxanthine)						112			[10]
Steroids	Ethynylestradiol (EE2)					<10				[15]
								<10		[8]
	Cholesterol							8700	90	[5]
	Coprostanol							5900	60	[5]
	Estradiol							3		[8]
	Estone							69		[8]
		ND	97	160		ND	3	30		[17]
	Fluocinonide						ND	ND	0	[12]
	Fluticasone						ND	ND	0	[12]
	Hydrocortisone						ND	ND	0	[12]
	Melengestrol Acetate						ND	ND	0	[12]
	Methylprednisolone						ND	ND	0	[12]
	Norethindrone						ND	ND	0	[12]
	Prednisolone						ND	ND	0	[12]
	Prednisone						ND	ND	0	[12]
	Progesterone							<0.5		[8]
						ND	<188	<188	4	[12]
	Sitosterol							2900	72.5	[5]
	Testosterone							<0.5		[8]
							ND	ND	0	[12]
Surfactants	4-*n*-Octylphenol		100				ND			[11]
	4-*Tert*-octylphenol					10		1860		[13]
		80	1340	3900		ND	48.4	100		[17]
	AEO		31213				167			[19]
	NPEO		24,363				314			[19]
	PFOA					14.8		65.2	100	[22]
		2		184		6.7		183		[23]
	PFOS					ND		47.7	95	[22]
		2.5		16		1.8		28		[23]
Ulcer Treatments	Allopurinol		10				ND			[11]
	Cimetidine					ND		410		[6]
			463				118			[19]
	Ranitidine					ND		550		[6]
							120	1400	38	[12]

Table S5. Occurrence of CECs that have been reported in WWTP influent and effluent in the United States (continued)

Groups	Compounds	WWTP Influent (ng/L)			Freq.	WWTP Effluent (ng/L)			Freq.	Ref.
		Minimum	Mean	Maximum	%	Minimum	Mean	Maximum	%	
Ulcer Treatments	Ranitidine		301				30			[19]
X-ray Contrast Media	Iopromide						<10			[8]

ND – not detected; Det – detected (concentration not specified); DEET – N,N-Diethyl-meta-toluamide; TCEP – tris-(2-chloroethyl)-phosphate; TCPP – tris-(2-chloroisopropyl)-phosphate; TBP – tributyl phosphate; DiOH-CBZ – 10,11-dihydro-10,11-dihydroxy-carbamazepine; 10-OH-CBZ – 10,11-dihydro-10-hydroxy-carbamazepine; Gluc-LMG – 2-N-glucuronide-lamotrigine; DMV – *O*-desmethyl-vorenlafaxine; PFOA – perfluorooctanoic acid; PFOS – perfluorooctanesulfonic acid.

AHAN – Acetyl-hexamethyl-tetrahydro-naphthalene; DBP – Dibutyl phthalate; DEHP – Di-(2-ethylhexyl) phthalate; NPEO – Nonylphenol Ethoxylate; AEO – Alcohol Ethoxylate; NPEC – Nonylphenol Polyethoxy Carboxylate; PEG – Polyethylene Glycol
[1] Huggett et al., 2003; [2] Vanderford and Snyder, 2006; [3] Yu et al., 2006; [4] Gross et al., 2004; [5] Glassmeyer et al., 2005; [6] Batt et al., 2008; [7] Boyd et al., 2003; [8] Gerrity et al., 2011; [9] Spongberg and Witter, 2008; [10] Bartelt-Hunt et al., 2009; [11] Bisceglia et al., 2010; [12] Kostich et al., 2014; [13] Bulloch et al., 2015; [14] Kwon and Rodriguez, 2014; [15] Yang et al., 2011; [16] Gao et al., 2012; [17] Yu et al., 2013; [18] Writer et al., 2013; [19] Lara-Martín et al., 2014; [20] Subedi and Kannan, 2015; [21] Chiaia et al., 2008; [22] Elmoznino et al., 2018; [23] Loganathan, et al., 2007.

Table S6. Occurrence of CECs that have been reported in surface water in the United States

Groups	Compounds	Surface Water (ng/L)			Detection Frequency	Ref.
		Minimum	Mean	Maximum	%	
Analgesics	Acetaminophen (Paracetamol)	ND		10000	23.8%	[3]
		ND		63		[6]
		ND		19	13%	[9]
		<2.5		73	24%	[10]
				25.8		[12]
				111.3		[14]
	Codeine	ND		1000	10.6%	[3]
		<3.6		15	29%	[10]
	Diclofenac	ND		24	50%	[9]
		18		124		[12], [13]
	Hydrocodone			16.7		[14]
	Ibuprofen	ND		1000	9.5%	[3]
		2		42	50%	[4]
			8			[8]
		ND		133	38%	[9]
		<25		40.5		[12], [13]
				43.6		[14]
	Indomethacin	ND		ND	0%	[4]
	Ketoprofen	0.4		0.8	9%	[4]
	Naproxen	<5		200		[1]
		1		14	18%	[4]
		ND		9	13%	[9]
		<1		31	24%	[10]
	Oxycodone			93.1		[14]
	Propoxyphene			6.4		[14]
Antagonists	Benztropine			3.7		[14]
Antiasthmas	Albuterol (Salbutamol)	ND		ND	0%	[3]
		<1.4		5.9	5%	[10]
				9.8		[14]
Antibiotics	Azithromycin	ND		670		[6]
		<3.7		22	24%	[10]
	Carbadox	<3.4		49	41%	[10]
		ND		ND	0%	[3]
	Chlortetracycline	ND		690	2.4%	[3]
	Ciprofloxacin	ND		30	2.6%	[3]

Table S6. Occurrence of CECs that have been reported in surface water in the United States (continued)

Groups	Compounds	Surface Water (ng/L)			Detection Frequency	Ref.
		Minimum	Mean	Maximum	%	
Antibiotics	Ciprofloxacin	8		8	5%	[4]
	Clarithryomycin	0.2		2	100%	[9]
	Doxycycline	ND		ND	0%	[3]
	Enrofloxacin	ND		ND	0%	[3]
	Erythromycin	3		34	18%	[4]
		0.3		3	100%	[9]
	Lavofloxacin	ND		1	38%	[9]
	Lincomycin	ND		120	1%	[3]
		0.1		1.8	36%	[4]
	Monensin	ND		35		[2]
	Narasin	ND		38		[2]
	Ofloxacin	<2.5		21	2%	[10]
	Oxacillin	<2.5		17	2%	[10]
	Oxytetracycline	ND		340	1.2%	[3]
	Roxithromycin	<4.3		39	21%	[10]
		0.3		1.7	9%	[4]
		ND		180	4.8%	[3]
	Salinomycin	ND		6		[2]
	Sarafloxacin	ND		ND	0%	[3]
	Sulfachloropyridazine	ND		ND	0%	[3]
	Sulfadimethoxine	ND		60	1.2%	[3]
		ND		3,956		[6]
	Sulfamerazine	ND		ND	0%	[3]
	Sulfamethazine	0.1		1.1	23%	[4]
		ND		472		[6]
	Sulfamethoxazole	ND		19000	12.5%	[3]
		0.2		2	41%	[4]
		ND		343		[6]
		ND		7	88%	[9]
		<4.1		77	83%	[10]
				932		[12]
				576.4		[14]
	Sulfanilamide	<2.9		20	5%	[10]
	Sulfathiazole	ND		ND	0%	[3]
		ND		7		[6]
	Tetracycline	ND		110	1.2%	[3]

Table S6. Occurrence of CECs that have been reported in surface water in the United States (continued)

Groups	Compounds	Surface Water (ng/L)			Detection Frequency	Ref.
		Minimum	Mean	Maximum	%	
Antibiotics	Tetracycline	ND		ND	0%	[4]
	Trimethoprim	ND		710	12.5%	[3]
		0.3		1	9%	[4]
			6.7			[8]
		2		11	100%	[9]
		<3.4		52	26%	[10]
				180		[12]
				60.9		[14]
	Tylosin	ND		280	13.5%	[3]
		2		40	23%	[4]
	Virginiamycin	ND		ND	0%	[3]
Anticoagulants	Warfarin	ND		ND	0%	[3]
				131.3		[14]
Antidepressants	Alprazolam			25		[14]
	Amitriptyline			0.8		[14]
	Diazepam			6.1		[12]
	Fluoxetine	ND		12	1.2%	[3]
		ND		1	38%	[9]
		<3.5		62	10%	[10]
				24.8		[14]
	Paroxetine			8.3		[14]
	Promethazine			1.5		[14]
	Sertraline			19		[14]
Antidiabetics	Metformin	ND		150	4.8%	[3]
		<0.5		9200	100%	[10]
Antiepileptics	Carbamazepine	0.1		2	86%	[4]
		14		116		[5]
		ND		296		[6]
		1		4	100%	[9]
		<2.7		38	21%	[10]
		203		330		[12], [13]
				249.3		[14]
	Phenytoin (Dilantin)			291		[12]
	Primidone	5		37		[5]
Antihistamines	Diphenhydramine	8		1,412		[6]
		<3.6		43	33%	[10]

Table S6. Occurrence of CECs that have been reported in surface water in the United States (continued)

Groups	Compounds	Surface Water (ng/L)			Detection Frequency	Ref.
		Minimum	Mean	Maximum	%	
Antihypertensions	Desmethyldiltiazem			16.8		[14]
	Diltiazem	ND		49	13.1%	[3]
		<3.5		21	29%	[10]
				56.8		[14]
	Enalapril			1.9		[14]
	Enalaprilat	ND		ND	0%	[3]
	Furosemide			37.2		[14]
	Hydrochlorothiazide			619.9		[14]
	Triamterene			23.6		[14]
	Valsartan			319.4		[14]
	Verapamil			35.8		[14]
Antioxidants	2,6-di-tert-butylphenol	ND		110	3.5%	[3]
	2,6-di-tert-butyl-1,4-benzoquinone	ND		460	9.4%	[3]
	5-Methyl-1H-Benzotriazle	ND		2400	31.5%	[3]
	BHA	ND		200	2.4%	[3]
	BHT	ND		100	2.4%	[3]
Antiseptics	Phenol	ND		1300	8.2%	[3]
	Triclocarban			102		[12]
		<0.5		9.9	12%	[10]
	Triclosan	ND		2300	57.6%	[3]
		ND		9.2	21%	[7]
		ND		106	63%	[9]
		<0.5		41	71%	[10]
		10.7		26.3		[12], [13]
β-blockers	Atenolol	ND		ND	0%	[9]
				186.4		[14]
	Metoprolol	0.1		0.3	100%	[9]
				217.7		[14]
	Propranolol		0.7			[8]
				11.2		[14]
Cardiac Stimulant	Digoxin	ND		ND	0%	[3]
Fire Retardants	BDE 47			2.2		[12]
	BDE 99			0.9		[12]
	TCEP	ND		540	57.6%	[3]

Table S6. Occurrence of CECs that have been reported in surface water in the United States (continued)

Groups	Compounds	Surface Water (ng/L)			Detection Frequency	Ref.
		Minimum	Mean	Maximum	%	
Fire Retardants	TCEP		19.3			[8]
		ND		53	88%	[9]
				785		[12]
	TCPP			2900		[12]
	TDCPP	ND		160	12.9%	[3]
				1345		[12]
Fragrances	1,4-Dichlorobenzene	ND		4300	25.9%	[3]
	Acetophenone	ND		410	9.4%	[3]
	Galaxolide (HHCB)	45		794		[11]
		2619		2753		[12], [13]
	Tonalide (AHTN)	56		112		[11]
				188		[12]
Lipid Regulators	Bezafibrate	0.5		4	32%	[4]
	Clofibric Acid	ND		100		[1]
		2		12	14%	[4]
	Gemfibrozil	ND		790	3.6%	[3]
		0.2		4	64%	[4]
		<1.6		43		[10]
		118		324		[13]
				112.5		[14]
Metabolites	10-Hydroxy-Amitriptyline			ND		[14]
	Cotinine	ND		900	38.1	[3]
		3		210		[6]
		ND		3	25%	[9]
		<3.5		21	19%	[10]
	Dehydronifedipine	ND		30	14.3%	[3]
	Desmethylsertraline (norsertraline)			7		[14]
	Digoxigenin	ND		ND	0%	[3]
	Erythromycin-H_2O	ND		1700	21.5%	[3]
	Nonylphenol (NP)	53		186	100%	[9]
	Norfluoxetine			15.4		[14]
	Norverapamil			30.4		[14]
Pesticides	Atrazine	3		75	100%	[9]
				17.1		[12]

Table S6. Occurrence of CECs that have been reported in surface water in the United States (continued)

Groups	Compounds	Surface Water (ng/L)			Detection Frequency	Ref.
		Minimum	Mean	Maximum	%	
Pesticides	Bifenthrin			3.6		[12]
	Carbaryl	ND		100	16.5%	[3]
	cis-Chlordane	ND		100	4.7%	[3]
	Chlorpyrifos	ND		310	15.3%	[3]
				4.9		[12]
	Diazinon	ND		350	25.9%	[3]
	Dieldrin	ND		210	4.7%	[3]
	DEET	ND		1100	74.1%	[3]
		7		1,617		[6]
		ND		68	66%	[7]
		2.3		3.3		[8]
		23		256	100%	[9]
				860		[12]
	Fipronil	22.2		28.7		[12], [13]
	Fipronil Desulfinyl			13.8		[12]
	Fipronil Sulfide			2		[12]
	Fipronil Sulfone			10.6		[12]
	Lindane	ND		110	5.9%	[3]
	Mecoprop	7.9		12.3		[8]
	Methyl Parathion	ND		10	1.2%	[3]
	Permethrin	<0.17		1.7		[12], [13]
	Thiabendazole	ND		27		[6]
Plasticizers	2-Butoxyethanol Phosphate	ND		6700	45.9%	[3]
	Bisphenol A	ND		12000	41.2%	[3]
		1		1967	50%	[4]
		ND		190	44%	[7]
		ND		22	75%	[9]
		<1		691		[12], [13]
	DEHA	ND		10000	3.5%	[3]
	DEHP	ND		20000	10.6%	[3]
	DEP	ND		420	11.1%	[3]
	Triphenyl Phosphate	ND		220	14.1%	[3]
Psycho-stimulants	Amphetamine			7.1		[14]
	Caffeine	ND		6000	61.9%	[3]
		86		687		[5]

Table S6. Occurrence of CECs that have been reported in surface water in the United States (continued)

Groups	Compounds	Surface Water (ng/L)			Detection Frequency	Ref.
		Minimum	Mean	Maximum	%	
Psycho-stimulants	Caffeine	16		980		[6]
		ND		68	85%	[7]
		5		6.2		[8]
		ND		16	50%	[9]
		21		230	98%	[10]
	Methamphetamine	ND		63		[6]
	Paraxanthine (1,7-dimethylxanthine)	ND		3100	28.6%	[3]
		2		180		[6]
		<11.6		57	45%	[10]
Steroids	17α-Ethynylestradiol (EE2)	ND		831	15.7%	[3]
	17β-Estradiol	ND		200	10.6%	[3]
		ND		1.8	21%	[7]
		<1.25		<5		[12], [13]
	4-androstene-3,17-dione	<0.5		17	31%	[10]
	Cholesterol	ND		60000	84.3%	[3]
		<150		2,896	96%	[7]
	cis-androsterone	ND		214	14.3%	[3]
	Coprostanol	ND		150000	85.7%	[3]
		ND		67	44%	[7]
	Coprostanone	ND		9.1	12%	[7]
	Equilenin	ND		278	2.8%	[3]
	Equilin	ND		147	1.4%	[3]
		ND		5.5	18%	[7]
	Estrone (E1)	ND		112	7.1%	[3]
		1		1	9%	[4]
		<10		200		[1]
		ND		5.2	81%	[7]
		<2.5		<5		[12], [13]
	Fluticasone			16.2		[14]
	Mestranol	ND		407	10%	[3]
	Norethindrone	ND		872	12.8%	[3]
	Progesterone	ND		199	4.3%	[3]
		<0.7		88	15%	[10]
				440.8		[14]

Table S6. Occurrence of CECs that have been reported in surface water in the United States (continued)

Groups	Compounds	Surface Water (ng/L)			Detection Frequency	Ref.
		Minimum	Mean	Maximum	%	
Steroids	Stigmastanol	ND		4000	5.6%	[3]
	Testosterone	ND		214	2.8%	[3]
		<1.1		38	15%	[10]
				361.4		[14]
Surfactants	PFOA	ND	23.3	65.5	95%	[15]
		0.9		1.8		[16]
		2.6×10^{6}		1.28×10^{9}		[17]
				210,000	88%	[18]
		ND		100	92%	[19]
		5.1		830		[20]
		10		173		[21]
		<0.05		173		[22]
		0.27		47	100%	[23]
		ND		11,000		[24]
	PFOS	ND	7.6	27.7	31.3%	[15]
		1.8		2.1		[16]
		2×10^{5}		3.68×10^{8}		[17]
				8,970,000	96%	[18]
		ND		43	33%	[19]
		<5		7.4		[20]
		0.8		1,090		[21]
		<0.23		2,930		[22]
		ND		23	95%	[23]
		<0.25		1,090		[24]
Ulcer Treatments	Cimetidine	ND		580	9.5%	[3]
	Ranitidine	ND		10	1.2%	[3]
		<0.9		27	3%	[10]
				21		[14]

BHA – Butylated hydroxyanisole; BHT – Butylated hydroxytoluene; DBP – Dibutyl phthalate; DEHA – Di-(2-ethylhexyl) adipate; DEHP – Di-(2-ethylhexyl) phthalate; DEP – Diethyl phthalateTCEP – tri(2-chloroethyl) phosphate; BDE 47 – Polybrominated diphenyl ethers-47; BDE 99 – Polybrominated diphenyl ethers 99; TCPP – tris-(2-chloroisopropyl)-phosphate; TDCPP – Tris(dichloroisopropyl) phosphate; PFOA – perfluorooctanoic acid; PFOS – perfluorooctanesulfonic acid.

[1] Boyd and Grimm, 2001; [2] Kim and Carlson, 2006; [3] Kolpin et al., 2002; [4] Tabe et al., 2009; [5] Guo and Krasner, 2009; [6] Bartelt-Hunt et al., 2009; [7] Singh et al., 2010; [8] Dougherty et al., 2010; [9] Padhye et al., 2014; [10] Blair et al., 2013; [11] Chase et al., 2012; [12] Sengupta et al., 2014; [13] Maruya et al., 2016; [14] Batt et al., 2016; [15] Bai and Son, 2021; [16] Goodrow et al., 2020; [17] Konwick et al., 2008; [18] Anderson et al., 2016; [19] Post et al., 2013; [20] Procopio et al., 2017; [21] Sinclair et al., 2006; [22] Vedagiri et al., 2018; [23] Zhang et al., 2016; [24] Crone et al., 2019.

Table S7. Occurrence of CECs that have been reported in source water and finished drinking water in the United States

Groups	Compounds	Source Water (ng/L)		Freq.	Finished Drinking Water (ng/L)		Freq.	Ref.
		Minimum	Maximum	%	Minimum	Maximum	%	
Analgesics	Acetaminophen (Paracetamol)	8	40	50%	ND	ND	0%	[1]
		ND	1890					[3]
		ND	160	8.1%				[5]
		ND	19.2	13%	ND	ND	0%	[8]
		<1	9.5		<1	<1		[10]
	Diclofenac	ND	1.2	21%	ND	ND	0%	[7]
		ND	24	50%	ND	9	25%	[8]
	Codeine	7	10		ND	ND	0%	[1]
		ND	214					[3]
		ND	<240	2.7%				[5]
	Ibuprofen				ND	ND	0%	[1]
		ND	5,850	8%	ND	1,350	13%	[2]
		ND	270					[5]
		ND	132.9	38%	ND	10	13%	[8]
		<1	24		<1	32		[10]
	Naproxen		44	70%				[4]
		ND	32	58%	ND	ND	0%	[7]
		ND	9.2	13%	ND	5	13%	[8]
		<1	11		<1	8		[10]
Antiasthmas	Albuterol (Salbutamol)				ND	ND	0%	[1]
		ND	ND					[3]
		ND	ND	0%				[5]
Antibiotics	Amoxicillin	25	2200	23%				[9]
	Azithromycin	ND	29	1.4%				[5]
	Carbadox				ND	ND	0%	[1]
		ND	ND	0%				[5]
	Chlortetracycline				ND	ND	0%	[1]
		ND	ND	0%				[5]
	Ciprofloxacin				ND	ND	0%	[1]
		ND	30	1.4%				[5]
	Clarithromycin	0.2	1.7	100%	ND	0.2	75%	[8]
	Demeclocycline				ND	ND	0%	[1]
		ND	ND	0%				[5]
	Doxycycline				ND	ND	0%	[1]
		ND	ND	0%				[5]

Table S7. Occurrence of CECs that have been reported in source water and finished drinking water in the United States (continued)

Groups	Compounds	Source Water (ng/L)		Freq.	Finished Drinking Water (ng/L)		Freq.	Ref.
		Minimum	Maximum	%	Minimum	Maximum	%	
Antibiotics	Enrofloxacin				ND	ND	0%	[1]
		ND	40	6.8%				[5]
	Erythromycin	<1	3.5		<1	1.5		[10]
	Levofloxacin	ND	1.3	38%	ND	ND	0%	[8]
	Lincomycin				ND	ND	0%	[1]
		ND	ND	0%				[5]
	Methotrexate				ND	ND	0%	[1]
		ND	ND	0%				[5]
	Minocycline				ND	ND	0%	[1]
		ND	ND	0%				[5]
	Norfloxacin				ND	ND	0%	[1]
		ND	ND	0%				[5]
	Oxytetracycline				ND	ND	0%	[1]
		ND	ND	0%				[5]
	Roxarsone				ND	ND	0%	[1]
	Roxithromycin				ND	ND	0%	[1]
		ND	ND	0%				[5]
	Sarafloxacin				ND	ND	0%	[1]
		ND	20	1.4%				[5]
	Sulfadimethoxine				ND	ND	0%	[1]
		ND	ND	0%				[5]
	Sulfamerazine				ND	ND	0%	[1]
		ND	ND	0%				[5]
	Sulfamethazine				ND	ND	0%	[1]
		ND	ND	0%				[5]
	Sulfamethizole				ND	ND	0%	[1]
		ND	ND	0%				[5]
	Sulfamethoxazole	4	10	17%	ND	ND	0%	[1]
		ND	170					[3]
			173	91%		3	13%	[4]
		ND	110	89%	ND	3	22%	[7]
		ND	7.4	88%	ND	13	13%	[8]
		17	990	28%				[9]
		<1	44		<1	20		[10]

Table S7. Occurrence of CECs that have been reported in source water and finished drinking water in the United States (continued)

Groups	Compounds	Source Water (ng/L)		Freq.	Finished Drinking Water (ng/L)		Freq.	Ref.
		Minimum	Maximum	%	Minimum	Maximum	%	
Antibiotics	Sulfathiazole				ND	ND	0%	[1]
		ND	ND	0%				[5]
	Tetracycline				ND	ND	0%	[1]
		ND	ND	0%				[5]
	Trimethoprim	0.5	6.5	83%	ND	ND	0%	[1]
		ND	18					[3]
			48	74%				[4]
		ND	20	6.8%				[5]
		ND	11	58%	ND	ND	0%	[7]
		2.2	10.9	5.8%	ND	19.8	88%	[8]
		<1	2.3		<1	1.3		[10]
	Tylosin				ND	ND	0%	[1]
		ND	ND	0%				[5]
	Virginiamycin				ND	ND	0%	[1]
		ND	ND	0%				[5]
Anticoagulants	Warfarin				ND	ND	0%	[1]
		ND	ND					[3]
		ND	ND	0%				[5]
Antidepressants	Diazepam	ND	0.47	11%	ND	0.33	6%	[7]
	Fluoxetine				ND	ND	0%	[1]
		ND	<18	1.4%				[5]
		ND	3	16%	ND	0.82	11%	[7]
		ND	0.9	38%	ND	19	13%	[8]
	Meprobamate		73	91%		43	83%	[4]
		ND	73	84%	ND	42	78%	[7]
		5.5	160	35%				[9]
		<1	16		<1	13		[10]
Antiepileptics	Carbamazepine	55	1500	100%	48	258	100%	[1]
		ND	420					[3]
			69	83%		18	61%	[4]
		ND	190	22%				[5]
		2	156					[6]
		ND	51	79%	ND	18	44%	[7]
		0.5	4.1	100%	ND	25	13%	[8]

Table S7. Occurrence of CECs that have been reported in source water and finished drinking water in the United States (continued)

Groups	Compounds	Source Water (ng/L)		Freq.	Finished Drinking Water (ng/L)		Freq.	Ref.
		Minimum	Maximum	%	Minimum	Maximum	%	
Antiepileptics	Carbamazepine	31	190	18%				[9]
		<1	39					[10]
	Phenytoin (Dilantin)		40	91%		32	74%	[4]
		ND	29	74%	ND	19	56%	[7]
		<1	5.3		<1	6.7		[10]
	Primidone	2	35					[6]
		20	54	25%				[9]
Antihistamine	Diphenhydramine	2	4	25%	ND	ND	0%	[1]
		ND	ND					[3]
	Diphenhydramine	ND	23	5.4%				[5]
Antihypertensions	Diltiazem				ND	ND	0%	[1]
		ND	ND					[3]
		ND	<12	1.4%				[5]
	Furosemide				ND	ND	0%	[1]
Antioxidants	5-Methyl-1H-Benzotriazle				ND	0ND	0%	[1]
	BHA				ND	ND	0%	[1]
		ND	3,520	15%	ND	3,450	7%	[2]
	BHT	ND	49	5%	ND	26	6%	[7]
Antipsychotics	Risperidone	ND	ND	0%	ND	ND	0%	[7]
Antiseptic	Phenol	210	600	67%	ND	ND	0%	[1]
		ND	<0.5	1.4%				[5]
	Triclosan	210	150	67%	ND	ND	0%	[1]
		ND	734	8%	ND	735	7%	[2]
			8.8	43%		1.2	9%	[4]
		ND	<1	8.1%				[5]
		ND	6.4	32%	ND	1.2	6%	[7]
		ND	105.8	63%	ND	60	63%	[8]
		<1	30		<1	43		[10]
β-blockers	Atenolol		48	74%		26	57%	[4]
		ND	36	63%	ND	18	44%	[7]
		ND	ND	0%	ND	34	13%	[8]
		6.1	200	27%				[9]
	Metoprolol	0.1	0.3	100%	ND	0.1	13%	[8]
		22	270	18%				[9]

Table S7. Occurrence of CECs that have been reported in source water and finished drinking water in the United States (continued)

Groups	Compounds	Source Water (ng/L)		Freq.	Finished Drinking Water (ng/L)		Freq.	Ref.
		Minimum	Maximum	%	Minimum	Maximum	%	
Cosmetics	Triethyl citrate (ethyl citrate)	50	80	50%	ND	62	50%	[1]
		500	560	5.4%				[5]
Fire Retardants	TBP	65	90	83%	80	100	83%	[1]
		ND	740	8.1%				[5]
	TCEP	70	160	100%	68	99	100%	[1]
			534	65%		470	48%	[4]
		ND	<0.5	20%				[5]
		ND	530	53%	ND	470	39%	[7]
		ND	51.7	88%	ND	20	88%	[8]
		7.9	66	57%				[9]
		<1	43		<10	19		[10]
	TCPP		721	57%		508	48%	[4]
		ND	720	42%	ND	510	28%	[7]
	TDCPP	80	300	100%	150	250	100%	[1]
		ND	<0.5	12%				[5]
Fragrances	1,4-Dichlorobenzene				ND	ND	0%	[1]
		ND	<500					[5]
	3-*methyl*-1(H)-indole (Skatole)				ND	ND	0%	[1]
		ND	<1,000					[5]
	Acetophenone				ND	ND	0%	[1]
	Galaxolide (HHCB)	45	120	92%	60	82	92%	[1]
			65	43%		34	17%	[4]
		ND	970					[5]
		ND	48	21%	ND	33	11%	[7]
		<25	30		<25	<25		[10]
	Tonalide (AHTN)	250	690	100%	310	490	100%	[1]
		ND	<500					[5]
Lipid Regulators	Atorvastatin	ND	1.4	16%	ND	ND	0%	[7]
	Clofibrate	ND	900	15%	ND	ND	0%	[2]
	Clofibric Acid	ND	630	8%	ND	ND	0%	[2]
	Gemfibrozil				ND	ND	0%	[1]
		ND	ND	0%				[5]
			38	78%		2	35%	[4]
		ND	24	58%	ND	2.1	39%	[7]

Table S7. Occurrence of CECs that have been reported in source water and finished drinking water in the United States (continued)

Groups	Compounds	Source Water (ng/L)		Freq.	Finished Drinking Water (ng/L)		Freq.	Ref.
		Minimum	Maximum	%	Minimum	Maximum	%	
Lipid Regulators	Gemfibrozil	13	130	20%				[9]
		<1	11		<1	6.5		[10]
Metabolites	Cotinine	13	35	100%	13	25	100%	[1]
		ND	ND					[3]
		ND	100					[5]
		ND	2.7	25%	ND	0.4	13%	[8]
		13	27	18%				[9]
	Erythromycin-H_2O	23	700	67%	ND	ND	0%	[1]
		ND	300	8.1%				[5]
		0.3	2.7	100%	1	14	100%	[8]
	Dehydronifedipine	1	60	50%	ND	4	50%	[1]
		ND	ND					[3]
		ND	19	4.1%				[5]
		7.7	120	25%				[9]
	Nonylphenol (NP)				ND	ND	0%	[1]
			141	48%		104	17%	[4]
		ND	130	42%	ND	110	11%	[7]
		53.4	185.6	100%	12	61	100%	[8]
	Diaminochlorotriazine	10	300	62%				[9]
	Norfluoxetine	ND	ND	0%	ND	ND	0%	[7]
	$NPEO_2$-total	2500	3000	58%	ND	ND	0%	[1]
	o-Hydroxy Atorvastatin	ND	1.2	16%	ND	ND	0%	[7]
	p-Hydroxy Atorvastatin	ND	2	16%	ND	ND	0%	[7]
Muscle Relaxants	Carisoprodol	5.4	43	20%				[9]
Pesticides	2,4-D	11	23	42%				[9]
	Atrazine		1,011	87%		990	91%	[4]
		ND	870	79%	ND	930	83%	[7]
		3.4	75.1	100%	1	15	100%	[8]
		<1	571		<1	<1		[10]
	Bromacil				ND	ND	0%	[1]
		ND	740					[5]
		6	290	31%				[9]
	Chlorpyrifos				ND	ND	0%	[1]
		ND	<500					[5]

Table S7. Occurrence of CECs that have been reported in source water and finished drinking water in the United States (continued)

Groups	Compounds	Source Water (ng/L)		Freq.	Finished Drinking Water (ng/L)		Freq.	Ref.
		Minimum	Maximum	%	Minimum	Maximum	%	
Pesticides	DEET	68	70	25%	ND	66	25%	[1]
		ND	131	8%	ND	ND	0%	[2]
			105	48%		99	43%	[4]
		ND	<500	14%				[5]
		ND	110	32%	ND	93	33%	[7]
		23.3	255.7	100%	0.5	24	100%	[8]
		2.2	67	80%				[9]
		3.9	28		<1	30		[10]
	Diazinon				ND	ND	0%	[1]
		ND	510					[5]
	Dichlorvos				ND	ND	0%	[1]
		ND	<1,000					[5]
	Digoxigenin				ND	ND	0%	[1]
	Diuron	5.3	940	51%				[9]
	Linuron					8.1	17%	[4]
	Linuron	ND	9.3	26%	ND	6.2	11%	[7]
	Metolachlor				ND	ND	0%	[1]
			119	48%		36	35%	[4]
		ND	670	39%				[5]
		ND	81	37%	ND	27	33%	[7]
		<10	174		<10	160		[10]
	Pentachlorophenol	150	230	33%	ND	ND	0%	[1]
		ND	<2	5.4%				[5]
	Prometon	80	96	25%	ND	96	25%	[1]
	Simazine	7.1	160	77%				[9]
	Thiabendazole	ND	ND					[3]
Plasticizers	2-Butoxyethanol Phosphate	ND	960					[5]
	BBP	ND	1,190	15%	ND	911	33%	[2]
		ND	54	11%	ND	ND	0%	[7]
	Bisphenol A	100	300	100%	110	420	100%	[1]
		ND	1,900					[5]
		ND	14	16%	ND	25	6%	[7]
		ND	21.9	75%	ND	44	50%	[8]
	Carbaryl				ND	ND	0%	[1]

Table S7. Occurrence of CECs that have been reported in source water and finished drinking water in the United States (continued)

Groups	Compounds	Source Water (ng/L)		Freq.	Finished Drinking Water (ng/L)		Freq.	Ref.
		Minimum	Maximum	%	Minimum	Maximum	%	
Plasticizers	Carbazole	80	102	17%	ND	ND	0%	[1]
	DBP	ND	8,340	31%	ND	2,730	7%	[2]
	DEHP	ND	5,940	15%	ND	2,680	13%	[2]
		ND	170	11%	ND	ND	0%	[7]
	DEP	ND	1490	15%	ND	2470	7%	[2]
	DMP	ND	784	38%	ND	54	7%	[2]
	DOP					117	35%	[4]
	TBEP	150	350	83%	150	350	83%	[1]
	Triphenyl Phosphate				ND	ND	0%	[1]
		ND	<0.5	1.4%				[5]
Psycho-stimulants	Caffeine	40	250	100%	48	119	100%	[1]
		ND	290					[3]
		ND	270	7.5%				[5]
		7	140					[6]
		ND	15.9	50%	ND	12	38%	[8]
		13	300	32%				[9]
		<10	87		<10	35		[10]
	p-Xanthine	ND	120					[3]
	Paraxanthine (1,7-dimethylxanthine)	90	160	75%	ND	ND	0%	[1]
		ND	300	23%				[5]
		8.9	23	18%				[9]
	Theobromine	6.4	67	35%				[9]
Steroids	17α-ethynylestradiol	ND	1.4	5%	ND	ND	0%	[7]
	17β-estradiol	ND	17	5%	ND	ND	0%	[7]
	β-sitosterol	850	5000	83%	ND	ND	0%	[1]
	β-stigmastanol				ND	ND	0%	[1]
		ND	<2	1.4%				[5]
	Cholesterol	800	2200	83%	ND	ND	0%	[1]
	Coprostanol	250	600	33%	ND	ND	0%	[1]
		ND	<2	18%				[5]
	Estrone (E1)		2	74%				[4]
		ND	0.9	79%	ND	ND	0%	[7]
		<1	1.4		<1	2.3		[10]

Table S7. Occurrence of CECs that have been reported in source water and finished drinking water in the United States (continued)

Groups	Compounds	Source Water (ng/L)		Freq.	Finished Drinking Water (ng/L)		Freq.	Ref.
		Minimum	Maximum	%	Minimum	Maximum	%	
Steroids	Genistein					2.9	9%	[4]
	Progesterone	ND	3.1	21%	ND	0.57	6%	[7]
	Testosterone	ND	1.2	11%	ND	ND	0%	[7]
Surfactants	4-*Tert*-octylphenol	ND	<1,000					[5]
	PFOA		112	76%		104	76%	[11]
			100	57%				[12]
		ND	31		ND	30		[13]
		<10	137					[14]
			100	76%	ND	4,300		[15]
	PFOS		48.3	88%		36.9	80%	[11]
			43	30%				[12]
		ND	41		ND	57		[13]
		<25	346					[14]
			400	50%	<0.25	15		[15]
	Surfynol	ND	818	31%	ND	161	27%	[2]
Sunscreens	4-n-Octylphenol				ND	ND	0%	[1]
	4-Tert-octylphenol				ND	ND	0%	[1]
	Benzophenone	50	99	58%	ND	130	58%	[1]
		ND	790	23%	ND	260	7%	[2]
	Hydrocinnamic acid	ND	20,300	23%	ND	20,100	20%	[2]
	OMC	ND	5,610	15%	ND	45	7%	[2]
Ulcer Treatments	Cimetidine				ND	ND	0%	[1]
		ND	ND	0%				[5]
	Ranitidine				ND	ND	0%	[1]
		ND	ND	0%				[5]
X-ray Contrast Media	Iohexal	73	960	29%				[9]
	Iopromide	<1	46		<1	31		[10]

WTP – Water treatment plant; TBEP – Tri(2-butoxyethyl) phosphate; TBP – Tributyl phosphate; TCEP – Tris(2-chloroethyl) phosphate; TCPP – Tris-(2-chloroisopropyl)-phosphate; TDCPP – Tris(dichloroisopropyl) phosphate; AHAN – Acetyl-hexamethyl-tetrahydro-naphthalene; BBP – Butyl benzyl phthalate; BHA – Butylated hydroxyanisole; BHT – Butylated hydroxytoluene; NPEO2-total – 4-nonylphenol diethoxylate; DEET – N,N-diethyl-meta-toluamide; DBP – Dibutyl phthalate; DEHP – Di-(2-ethylhexyl) phthalate; DEP – Diethyl phthalate; DMP – Dimethyl phthalate; DOP – Dioctyl phthalate; OMC – Octyl methoxycinnamate; 2,4-D – 2,4-dichlorophenoxyacetic acid; PFOA – perfluorooctanoic acid; PFOS – perfluorooctanesulfonic acid.

[1] Stackelberg et al., 2004; [2] Loraine et al., 2006; [3] Fram and Belitz, 2011; [4] Snyder et al., 2008; [5] Focazio et al., 2008; [6] Guo and Krasner, 2009; [7] Benotti et al., 2009; [8] Padhye et al., 2014; [9] Oppenheimer et al., 2011; [10] Snyder et al., 2007; [11] Boone et al., 2019; [12] Post et al., 2013; [13] Quiñones and Snyder, 2009; [14] Sun et al., 2016; [15] Crone et al., 2019.

Table S8. Properties of Analgesics

Analgesics	Acetaminophen	Ibuprofen
Structure[6,7]	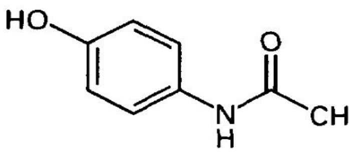	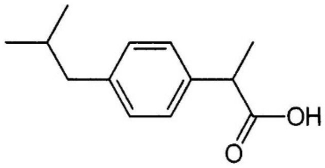
Chemical Name	N-(4-hydroxyphenyl)-acetamide	α-methyl-4-(2-methylpropyl)-benzeneacetic acid
Molecular Formula[2]	$C_8H_9NO_2$	$C_{13}H_{18}O_2$
Aqueous Solubility[1,5]	Value: 1.4×10^4 mg/L<br>Temp: 25 ºC	Value: 21 mg/L<br>Temp: 25 ºC
Boiling Point[8]	387.8±25.0 ºC	319.6±11.0 ºC
Molecular Weight[2]	151.17	206.23
Vapor Pressure[8]	1.43×10^{-6} Torr<br>Temp: 25 ºC	1.39×10^{-4} Torr<br>Temp: 25 ºC
Log K_{ow}[3]	0.46	3.97
pK_a[2,3]	9.38	4.91
k_{O3}[9,10,12]	2.70×10^5 $M^{-1}s^{-1}$ (pH 7)	9.6 (±1) $M^{-1}s^{-1}$ (pH 7, 20ºC)
K_{OH}[9,10,11]	–	6.5×10^9-7.4 (±1.2)$\times10^9$ $M^{-1}s^{-1}$<br>(pH 7, 25ºC)
K_{oc}[4,13]	170-1300 mL·g C^{-1}	18-155 mL·g C^{-1}
Structural Features[1]	Phenol, amide	Aromatic ring, carboxylic acid

[1] Snyder et al. (2007); [2] Gros et al. (2006); [3] Schwab et al. (2005); [4] Scheytt et al., 2005; [5] DrugBank (2018); [6] Daughton (1999); [7] Tixier et al. (2003); [8] Calculated using Advanced Chemistry Development (ACD/Labs) Software V11.02 (© 1994-2016 ACD/Labs); [9] Huber et al. (2003); [10] Huber et al. (2005); [11] Nanaboina et al. (2010); [12] Javier Rivas et al. (2010); [13] Yamamoto et al., 2009.

Table S9. Properties of Antibiotics

Antibiotics	Erythromycin	Sulfamethoxazole	Trimethoprim
Structure[4]	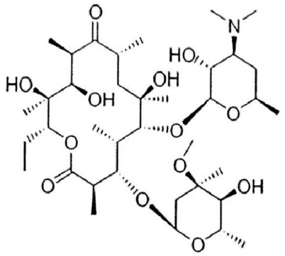	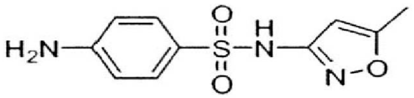	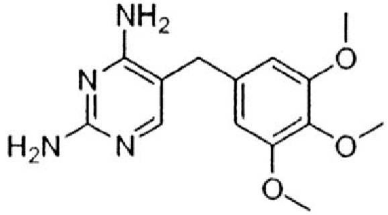
Chemical Name	Erythromycin	4-amino-N-(5-methyl-3-isoxazolyl)-benzenesulfonamide	5-[(3,4,5-trimethoxyphenyl)methyl]-2,4-Pyrimidinediamine
Molecular Formula[1]	$C_{37}H_{67}O_{13}$	$C_{10}H_{11}N_3O_3S$	$C_{14}H_{18}N_4O_3$
Aqueous Solubility[1]	Value: 1.44 mg/L<br>Temp: 25 ºC	Value: 610 mg/L<br>Temp: 37 ºC	Value: 400 mg/L<br>Temp: 25 ºC
Boiling Point[5]	818.4±65.0 ºC	482.1±55.0 ºC	526.0±60.0 ºC
Molecular Weight[4]	733.93 g/mol	253.28 g/mol	290.32 g/mol
Vapor Pressure[5]	4.94×10^{-31} Torr<br>Temp: 25 ºC	1.89×10^{-9} Torr<br>Temp: 25 ºC	3.74×10^{-11} Torr<br>Temp: 25 ºC
Log K_{ow}[2]	3.06	0.89	0.91
pK_a[3]	8.8	6.0	7.12
k_{O3}[6,7,8]	–	5.5×10^{5}-2.5×10^{6} $M^{-1}s^{-1}$<br>(pH 7, 20ºC)	2.7×10^{5} $M^{-1}s^{-1}$ (pH 7, 20ºC)
K_{OH}[6,7]	–	5.5 (±0.7)$\times10^{9}$ $M^{-1}s^{-1}$ (pH 7, 25ºC)	6.9 (±0.2)$\times10^{9}$ $M^{-1}s^{-1}$ (pH 7, 25ºC)
K_{oc}[9]	570 mL·g C^{-1}	72 mL·g C^{-1}	75 mL·g C^{-1}
Structural Features[1]	Complex aliphatic structure, ketone, alcohols, ester, ethers, tertiary amine, heterocyclic rings	Sulfone, primary amine, secondary amine, aromatic ring, isoxazole ring	Aromatic ring, pyrimidine ring, primary amines, methoxy groups

[1] Snyder et al. (2007); [2] Schwab et al. (2005); [3] Gros et al. (2006); [4] Yargeau and Leclair, (2008); [5] Calculated using Advanced Chemistry Development (ACD/Labs) Software V11.02 (© 1994-2016 ACD/Labs); [6] Huber et al. (2003); [7] Huber et al. (2005); [8] Dodd et al. (2006); [9] Hazardous Substances Data Bank (HSDB).

Table S10. Properties of Antidepressants

Antidepressants	Diazepam	Fluoxetine	Meprobamate
Structure[5]	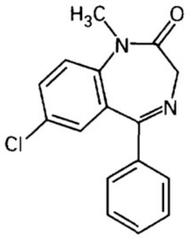	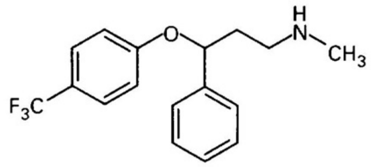	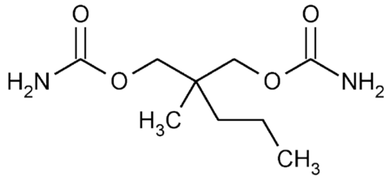
Chemical Name	7-chloro-1,3-dihydro-1-methyl-5-phenyl-2H-1,4-benzodiazepin-2-one	N-methyl-γ-[4-(trifluoromethyl)phenoxy]-benzenepropanamine	2-methyl-2-propylpropane-1,3-diol dicarbamate
Molecular Formula[5]	$C_{16}H_{13}ClN_2O$	$C_{17}H_{18}F_3NO$	$C_9H_{18}N_2O_4$
Aqueous Solubility[1, 7]	Value: 50 mg/L<br>Temp: 25 ºC	Value: 60.3 mg/L<br>Temp: 25 ºC	Value: 4,700 mg/L
Boiling Point[6]	297.4±45.0 ºC	395.1±42.0 ºC	344.5±89.0 ºC
Molecular Weight[5]	284.74 g/mol	309.3 g/mol	218.25g/mol
Vapor Pressure[6]	4.98×10^{-10} Torr<br>Temp: 25 ºC	1.88×10^{-6} Torr<br>Temp: 25 ºC	3.05×10^{-3} Torr<br>Temp: 25 ºC
Log K_{ow}[1,2,7]	2.70	2.60	0.70
pK_a[3,4,6]	2.4	8.7	9.2
k_{O3}[8]	0.75±0.15 $M^{-1}s^{-1}$	-	-
K_{OH}[8]	7.2 (±1.0)$\times10^9$ $M^{-1}s^{-1}$ (pH 7, 25ºC)	-	-
Structural Features[1]	Aromatics rings, heterocyclic ring, cyclic amide	Aromatic rings, secondary amine, fluorines	Aliphatic, carbamates

[1] Snyder et al. (2007); [2] Schwab et al. (2005); [3] Gros et al. (2006); [4] Yoon et al. (2007); [5] Daughton (1999); [6] Calculated using Advanced Chemistry Development (ACD/Labs) Software V11.02 (© 1994-2016 ACD/Labs); [7] Kwon and Armbrust, (2008); [8] Huber et al., 2003.

Table S11. Properties of Antiepileptic

**Antiepileptic**	**Carbamazepine**
Structure	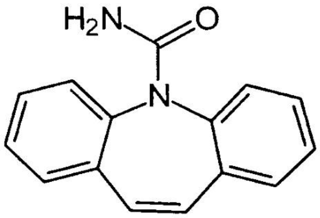
Chemical Name	5H-dibenzazepine-5-carboxamide
Molecular Formula[2]	$C_{15}H_{12}\ N_2O$
Aqueous Solubility[1]	Value: 17.7 mg/L<br>Temp: 25 ºC
Boiling Point[4]	411.0±48.0 ºC
Molecular Weight[2]	236.27 g/mol
Vapor Pressure[4]	5.78×10^{-7} Torr<br>Temp: 25 ºC
Log K_{ow}[2]	2.47
pK_a[2]	7
k_{O3}[5,6]	$3\times10^{5}\ M^{-1}s^{-1}$ (pH 7, 20 ºC)
K_{OH}[5,6]	8.8 (±1.2)$\times10^{9}\ M^{-1}s^{-1}$ (pH 7, 25 ºC)
K_{oc}[7]	510 mL·g C^{-1}
Structural Features	Aromatic rings, heterocyclic ring, amide

[1] Snyder et al. (2007); [2] Gros et al. (2006); [3] Tixier et al. (2003); [4] Calculated using Advanced Chemistry Development (ACD/Labs) Software V11.02 (© 1994-2016 ACD/Labs); [5] Huber et al. (2003); [6] Huber et al. (2005); [7] Hazardous Substances Data Bank (HSDB).

Table S12. Properties of β-Blockers

Beta Blockers	Atenolol	Propranolol
Structure[5]	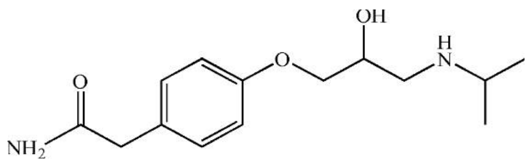	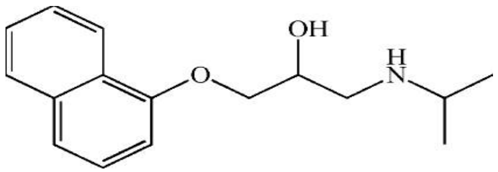
Chemical Name	4-[2-hydroxy-3-[(1-methylethyl)amino]propoxy]-benzeneacetamide	1-[(1-methylethyl)amino]-3-(1-naphthalenyloxy)- 2-propanol
Molecular Formula[1]	$C_{14}H_{22}N_2O_3$	$C_{16}H_{21}NO_2$
Aqueous Solubility[3,4]	26.7 mg/L	70 mg/L
Boiling Point[6]	508.0±50.0 ºC	434.9±30.0 ºC
Molecular Weight[1]	266.34	259.80
Vapor Pressure[6]	3.82×10^{-11} Torr<br>Temp: 25 ºC	2.48×10^{-8} Torr<br>Temp: 25 ºC
Log K_{ow}[1,2]	0.16	3.48
pK_a[1,2]	9.6	9.58
k_{O3}[7,8]	1.7 (±0.4)$\times10^{3}$ $M^{-1}s^{-1}$ (pH 7, 20-22 ºC)	1.0×10^{5} $M^{-1}s^{-1}$
K_{OH}[7,8]	8.0 (±0.5)$\times10^{9}$ $M^{-1}s^{-1}$ (pH 7, 20-22 ºC)	1.0×10^{10} $M^{-1}s^{-1}$
K_{oc}[9]	148-1700 mL·g C^{-1}	–
Structural Features	Aromatic ring, carboxylic acid, ether, amine	Aromatic ring, carboxylic acid, ether, amine

[1] Gros et al. (2006); [2] de Ridder et al. (2009); [3] Hatem et al. (1996); [4] DrugBank (2018); [5] Lavén et al. (2009); [6] Calculated using Advanced Chemistry Development (ACD/Labs) Software V11.02 (© 1994-2016 ACD/Labs); [7] Hollender et al. (2009); [8] Reungoat et al. (2010); [9] Yamamoto et al., 2009; [10] Drori et al., 2005.

Table S13. Properties of Blood Lipid Regulators

**Blood Lipid Regulators**	**Gemfibrozil**	**Clofibric Acid**
Structure[1, 3]	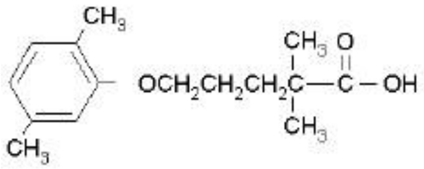	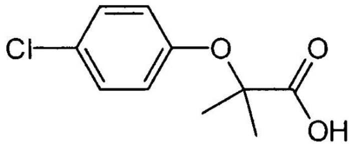
Chemical Name[2]	5-(2,5-dimethylphenoxy)-2,2-dimethyl-Pentanoic acid	2-(4-chlorophenoxy)-2-methyl-propanoic acid
Molecular Formula[1, 4]	$C_{15}H_{22}O_3$	$C_{10}H_{11}O_3Cl$
Aqueous Solubility[1]	19 mg/L	Value: 583 mg/L<br>Temp: 25 ºC
Boiling Point[2]	394.7±30.0 ºC	324.1±17.0 ºC
Molecular Weight[1, 4]	250.16	214.5
Vapor Pressure[2]	6.13×10^{-7} Torr<br>Temp: 25 ºC	1.03×10^{-4} Torr<br>Temp: 25 ºC
Log K_{ow}[1, 4, 5]	4.77	2.88
pK_a[1]	4.42	3.2
k_{O3}[3, 5]	2.0×10^{3} $M^{-1}s^{-1}$	20 $M^{-1}s^{-1}$
K_{OH}[3]	1.0×10^{10} $M^{-1}s^{-1}$	4.7×10^{9} $M^{-1}s^{-1}$
K_{oc}[4]	430 mL·g C^{-1}	-
Bioconcentration Factor[2]	1100-6.39 (1≤pH≤7, 25°C);<br>1 (8≤pH≤10, 25°C)	40.7-5.44 (1≤pH≤4, 25°C);<br>1 (5≤pH≤10, 25°C)
Structural Features[1]	Aromatic ring, carboxylic acid, ether	Aromatic ring, carboxylic acid, ether

[1] Snyder et al. (2007); [2] Calculated using Advanced Chemistry Development (ACD/Labs) Software V11.02 (© 1994-2016 ACD/Labs); [3] Nanaboina et al. (2010); [4] Hazardous Substances Data Bank (HSDB); [5] Huber et al. (2005).

Table S14. Properties of Pesticides

Pesticides	Atrazine	Metolachlor	DEET	Aminotriazole
Structure[1]	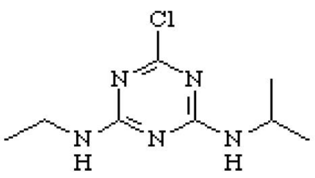	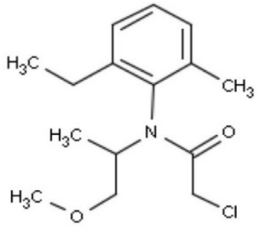	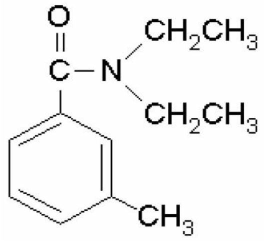	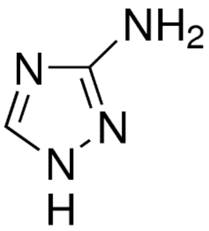
Chemical Name	6-chloro-N-2-ethyl-N4-(1-methylethyl)-1,3,5-triazine-2,4-diamine	2-chloro-N-(2-ethyl-6-methylphenyl)-N-(2-methoxy-1-methylethyl)-acetamide	N,N-diethyl-3-methyl-benzamide	3-amino-1,2,4-triazole
Molecular Formula[1]	$C_8H_{14}ClN_5$	$C_{15}H_{22}ClNO_2$	$C_{12}H_{17}NO$	$C_2H_4N_4$
Aqueous Solubility[1,2]	Value: 34.7 mg/L<br>Temp: 26 ºC	Value: 530 mg/L<br>Temp: 20 ºC	Value: 912 mg/L<br>Temp: 25 ºC	Values: 280,000 mg/L<br>Temp: 25 ºC
Boiling Point[3]	368.5±25.0 ºC	406.8±45.0 ºC	297.5±0.0 ºC	347.2±25.0 °C
Molecular Weight[1]	215.1 g/mol	283.8 g/mol	191.13 g/mol	84.08 g/mol
Vapor Pressure[3]	1.27×10^{-5} Torr<br>Temp: 25 ºC	7.91×10^{-7} Torr<br>Temp: 25 ºC	1.53×10^{-3} Torr<br>Temp: 25 ºC	5.45×10^{-5} Torr<br>Temp: 25 ºC
Log K_{ow}[1,2]	2.61	3.13	2.18	-0.97
pK_a[1,3]	1.7	-1.34	0.67	11.14
k_{O3}[4,6,7]	6.0-7.9 $M^{-1}s^{-1}$	3.0 $M^{-1}s^{-1}$	1.0 $M^{-1}s^{-1}$	–
K_{OH}[4,5,6,7]	2.4×10^9-3.0×10^9 $M^{-1}s^{-1}$	–	5.0×10^9 $M^{-1}s^{-1}$	–
K_{oc}[8,9,10]	23-101 mL·g C^{-1}	–	300 mL·g C^{-1}	–
Structural Features[1]	Triazine ring, secondary amines, chlorine	Aromatic ring, amide, methoxy, chlorine	Aromatic ring, amide	Triazine ring, amines

[1] Snyder et al. (2007); [2] Fontecha-Cámara et al. (2007); [3] Calculated using Advanced Chemistry Development (ACD/Labs) Software V11.02 (© 1994-2016 ACD/Labs); [4] Hollender et al. (2009); [5] Huber et al. (2003); [6] Westerhoff et al. (2005); [7] Acero et al. (2000); [8] Nkedi-Kizza et al. (2006); [9] Drori et al. (2005); [10] Hazardous Substances Data Bank (HSDB).

Table S15. Properties of Steroids

Steroids	Cholesterol	17β-estradiol
Structure[6]	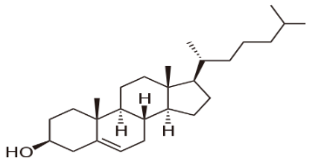	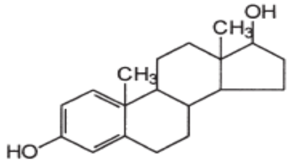
Chemical Name	(3β)-cholest-5-en-3-ol	(17β)- Estra-1,3,5(10)-triene-3,17-diol
Molecular Formula	$C_{27}H_{46}O$	$C_{18}H_{24}O_2$
Aqueous Solubility[1,4,8]	Value: 0.095 mg/L<br>Temp: 30 º C	Value: 3.6 mg/L
Boiling Point[7]	480.6±14.0 º C	445.9±45.0 ºC
Molecular Weight	386.65 g/mol	272.38g/mol
Vapor Pressure[7]	2.95×10^{-11} Torr<br>Temp: 25 ºC	9.82×10^{-9} Torr<br>Temp: 25 ºC
Log K_{ow}[2,3,5,6]	8.74	4.01
pK_a[3,7]	15.03	10.4
k_{O3}[9]	–	1.0×10^{6} $M^{-1}s^{-1}$ (20 ºC)
K_{OH}[9]	–	1.41×10^{10} $M^{-1}s^{-1}$
K_{oc}[10,11,12]	–	3700-10000 mL·g C^{-1}
Structural Features[6]	Alcohol, aliphatic rings	Phenol, alcohol, aliphatic rings

[1] Human Metabolome Database (2018); [2] Elkins and Mullis (2006); [3] Nghiem et al. (2004); [4] DrugBank (2018); [5] Yoon et al. (2007); [6] Snyder et al. (2007); [7] Calculated using Advanced Chemistry Development (ACD/Labs) Software V11.02 (© 1994-2016 ACD/Labs); [8] Hakk et al. (2005); [9] Broséus et al. (2009); [10] Yamamoto et al. (2005); [11] Carballa et al. (2008); [12] Karnjanapiboonwong et al. (2010).

Table S16. Properties of Other PPCPs

Compounds	TCEP	Caffeine	PFOA	PFOS	Cotinine	Iopromide
Structure[1,17]	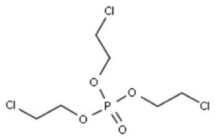	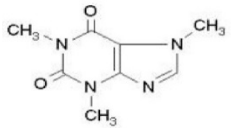	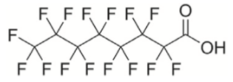	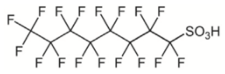	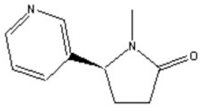	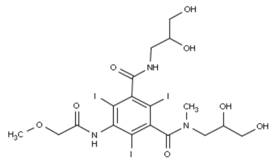
Chemical Name	2-chloro-phosphate (3:1) ethanol	3,7-dihydro-1,3,7-trimethyl-1H-Purine-2,6-dione	Perfluorooctanoic acid	Perfluorooctane sulfonic acid	1-methyl-5-(3-pyridinyl)-, (5S)-2-Pyrrolidinone	N1,N3-bis(2,3-dihydroxypropyl)-2,4,6-triiodo-5-[(2-methoxyacetyl)amino]-N1-methyl-1,3-Benzenedicarboxamide
Molecular Formula[1,15]	$C_6H_{12}Cl_3O_4P$	$C_8H_{10}N_4O_2$	$C_7F_{15}COOH$	$C_8F_{17}SO_3H$	$C_{10}H_{12}N_2O$	$C_{18}H_{24}I_3N_3O_8$
Aqueous Solubility[1,3,4,15]	Value: 7,000 mg/L	Value: 2.16 × 10^4mg/L<br>Temp: 25 º C	Value: 2.29 × 10^3mg/L<br>Temp: 24 º C	Value: 3.2 × 10^{-3}mg/L<br>Temp: 25 º C	Value: 1.17 × 10^5 mg/L	Value: 23.8 mg/L<br>Temp: 25 ºC
Boiling Point[6,15]	347.4±0.0 º C	416.8±37.0 ºC	192 º C	249 º C	316.0±0.0 º C	840.9±65.0 ºC
Molecular Weight[1,15]	285.5 g/mol	194.1 g/mol	414.07	500.126	176.1 g/mol	791.11 g/mol
Vapor Pressure[6,15]	1.08×10^{-4} Torr<br>Temp: 25 º C	3.72×10^{-7} Torr<br>Temp: 25 º C	3.16×10^{-2} mm Hg<br>Temp: 25 º C	2×10^{-3} mm Hg<br>Temp: 25 º C	4.21×10^{-4} Torr<br>Temp: 25 º C	5.00×10^{-30} Torr<br>Temp: 25 ºC
Log K_{ow}[1,4,15]	1.44	-0.07	4.81	4.49	0.04	-2.05
pK_a[1,2,4,15]	7.6	10.4	-0.5—4.2	<1	4.72	-2.60
k_{O3}[7,8,9,11]	–	6.50 (±0.2)×10^2 $M^{-1}s^{-1}$ (20 ºC)	–	–	–	< 0.8 $M^{-1}s^{-1}$ (pH 7, 20ºC)
K_{OH}[7,8,9,10,11,12]	5.60 (±0.21)×10^8 $M^{-1}s^{-1}$	5.9×10^9-6.9×10^9 $M^{-1}s^{-1}$	–	–	–	3.3 (±0.6)×10^9 $M^{-1}s^{-1}$ (pH 7, 25ºC)
K_{oc}[13,14,16]	67 mL·g C^{-1}	22 mL·g C^{-1}	29.51 mL·g C^{-1}	125.89 mL·g C^{-1}	130 mL·g C^{-1}	0.005 mL·g C^{-1}
Structural Features[1]	Phosphate, chlorines, aliphatic structure	Xanthine ring	Carbon-fluorine bond	Carbon-fluorine bond	Ketone, pyridine ring	Aromatic ring, iodines, alcohols, methoxy, amides

[1] Snyder et al. (2007); [2] Dmitrenko et al. (2007); [3] Trenholm et al. (2006); [4] Cone and Huestis (2007); [5] Human Metabolome Database (2018); [6] Calculated using Advanced Chemistry Development (ACD/Labs) Software V11.02 (© 1994-2016 ACD/Labs); [7] Hollender et al. (2009); [8] Huber et al. (2003); [9] Huber et al. (2005); [10] Westerhoff et al. (2005); [11] Broséus et al. (2009); [12] Watts and Linden (2009); [13] Hazardous Substances Data Bank (HSDB); [14] Carballa et al., 2008; [15] Gagliano et al., 2020; [16] Rahman et al., 2014; [17] Trojanowicz et al., 2018.

References:

Acero, J.L., Stemmler, K., Von Gunten, U., 2000. Degradation kinetics of atrazine and its degradation products with ozone and OH radicals: A predictive tool for drinking water treatment. Environ. Sci. Technol. 34 (4), 591-597.

Anderson, R.H., Long, G.C., Porter, R.C., Anderson, J.K., 2016. Occurrence of select perfluoroalkyl substances at U.S. Air Force aqueous film-forming foam release sites other than fire-training areas: Field-validation of critical fate and transport properties. Chemosphere 150, 678-685.

Bai, X., Son, Y., 2021. Perfluoroalkyl substances (PFAS) in surface water and sediments from two urban watersheds in Nevada, USA. Sci. Total Environ. 751,141622.

Bartelt-Hunt, S.L., Snow, D.D., Damon, T., Shockley, J., Hoagland, K., 2009. The occurrence of illicit and therapeutic pharmaceuticals in wastewater effluent and surface waters in Nebraska. Environ. Pollut. 157 (3), 786-791.

Batt, A.L., Kincaid, T.M., Kostich, M.S., Lazorchak, J.M., Olsen, A.R., 2016. Evaluating the extent of pharmaceuticals in surface waters of the United States using a National-scale Rivers and Streams Assessment survey. Environ. Toxicol. Chem. 35 (4), 874-881.

Batt, A.L., Kostich, M.S., Lazorchak, J.M., 2008. Analysis of ecologically relevant pharmaceuticals in wastewater and surface water using selective solid-phase extraction and UPLC-MS/MS. Anal. Chem. 80 (13), 5021-5030.

Benotti, M.J., Trenholm, R.A., Vanderford, B.J., Holady, J.C., Stanford, B.D., Snyder, S.A., 2009. Pharmaceuticals and endocrine disrupting compounds in U.S. drinking water. Environ. Sci. Technol. 43 (3), 597-603.

Bisceglia, K.J., Yu, J.T., Coelhan, M., Bouwer, E.J., Roberts, A.L., 2010. Trace determination of pharmaceuticals and other wastewater-derived micropollutants by solid phase extraction and gas chromatography/mass spectrometry. J. Chromatogr. A 1217 (4), 558-564.

Blair, B.D., Crago, J.P., Hedman, C.J., Klaper, R.D., 2013. Pharmaceuticals and personal care products found in the Great Lakes above concentrations of environmental concern. Chemosphere 93 (9), 2116-2123.

Boone, J.S., Vigo, C., Boone, T., Byrne, C., Ferrario, J., Benson, R., Donohue, J., Simmons, J.E., Kolpin, D.W., Furlong, E.T., Glassmeyer, S.T., 2019. Per- and polyfluoroalkyl substances in source and treated drinking waters of the United States. Sci. Total Environ. 653, 359-369.

Boyd, G.R., Grimm, D.A., 2001. Occurrence of pharmaceutical contaminants and screening of treatment alternatives for southeastern Louisiana. Ann. N. Y. Acad. Sci. 948, 80-89.

Boyd, G.R., Reemtsma, H., Grimm, D.A., Mitra, S., 2003. Pharmaceuticals and personal care products (PPCPs) in surface and treated waters of Louisiana, USA and Ontario, Canada. Sci. Total Environ. 311 (1-3), 135-149.

Broséus, R., Vincent, S., Aboulfadl, K., Daneshvar, A., Sauvé, S., Barbeau, B., Prévost, M., 2009. Ozone oxidation of pharmaceuticals, endocrine disruptors and pesticides during water treatment. Water Res. 43 (18), 4707-4717.

Bulloch, D.N., Nelson, E.D., Carr, S.A., Wissman, C.R., Armstrong, J.L., Schlenk, D., Larive, C.K., 2015. Occurrence of halogenated transformation products of selected pharmaceuticals and personal care products in secondary and tertiary treated wastewaters from Southern California. Environ. Sci. Technol. 49 (4), 2044-2051.

Carballa, M., Fink, G., Omil, F., Lema, J.M., Ternes, T., 2008. Determination of the solid–water distribution coefficient (Kd) for pharmaceuticals, estrogens and musk fragrances in digested sludge. Water Res. 42 (1-2), 287-295.

Chase, D.A., Karnjanapiboonwong, A., Fang, Y., Cobb, G.P., Morse, A.N., Anderson, T.A., 2012. Occurrence of synthetic musk fragrances in effluent and non-effluent impacted environments. Sci. Total Environ. 416, 253-260.

Chiaia, A.C., Banta-Green, C., Field, J., 2008. Eliminating solid phase extraction with large-volume injection LC/MS/MS: Analysis of illicit and legal drugs and human urine indicators in US wastewaters. Environ. Sci. Technol. 42 (23), 8841-8848.

Crone, B.C., Speth, T.F., Wahman, D.G., Smith, S.J., Abulikemu, G., Kleiner, E.J., Pressman, J.G., 2019. Occurrence of per- and polyfluoroalkyl substances (PFAS) in source water and their treatment in drinking water. Crit. Rev. Environ. Sci. Technol. 49 (24), 2359-2396.

Cone, E. J., Huestis, M. A. (2007) Interpretation of oral fluid tests for drugs of abuse. Ann. N. Y. Acad. Sci. 1098, 51-103.

Daughton, C.G., Ternes, T.A., 1999. Pharmaceuticals and personal care products in the environment: agents of subtle change? Environ. Health Perspect. 107 (6), 907-938.

de Ridder, D.J., McConville, M., Verliefde, A.R.D., Van De Aa, L.T.J., Heijman, S.G., Verberk, J.Q.J.C., Rietveld, L.C., Van Dijk, J.C., 2009. Development of a predictive model to determine micropollutant removal using granular activated carbon. Drinking Water Eng. Sci. 2 (2), 57-62.

Dmitrenko, O., Thorpe, C., Bach, R.D., 2007. Mechanism of SN2 disulfide bond cleavage by phosphorus nucleophiles. Implications for biochemical disulfide reducing agents. J. Org. Chem. 72 (22), 8298-8307.

Dodd, M.C., Buffle, M.-O., Von Gunten, U., 2006. Oxidation of antibacterial molecules by aqueous ozone: moiety-specific reaction kinetics and application to ozone-based wastewater treatment. Environ. Sci. Technol. 40 (6), 1969-1977.

Dougherty, J.A., Swarzenski, P.W., Dinicola, R.S., Reinhard, M., 2010. Occurrence of herbicides and pharmaceutical and personal care products in surface water and groundwater around Liberty Bay, Puget Sound, Washington. J. Environ. Qual. 39 (4), 1173-1180.

Drori, Y., Aizenshtat, Z., Chefetz, Benny., 2005. Sorption-desorption behavior of atrazine in soils irrigated with reclaimed wastewater. Soil Sci. Soc. Am. J. 69 (6), 1703-1710.

DrugBank. DrugBank version 5.1.0, http://www.drugbank.ca (accessed April 2, 2018).

Elkins, C.A., Mullis, L.B., 2006. Mammalian steroid hormones are substrates for the major RND- and MFS-type tripartite multidrug efflux pumps of Escherichia coli. J. Bacteriol. 188 (3), 1191-1195.

Elmoznino, J., Vlahos, P., Whitney, M., 2018. Occurrence and partitioning behavior of perfluoroalkyl acids in wastewater effluent discharging into the Long Island Sound. Environ. Pollut. 243, 453-461.

Fontecha-Cámara, M.Á., López-Ramón, M.V., Álvarez-Merino, M.A., Moreno-Castilla, C., 2007. Temperature dependence of herbicide adsorption from aqueous solutions on activated carbon fiber and cloth. Langmuir 22 (23), 9586-9590.

Fram, M.S., Belitz, K., 2011. Occurrence and concentrations of pharmaceutical compounds in groundwater used for public drinking-water supply in California. Sci. Total Environ. 409 (18), 3409-3417.

Focazio, M.J., Kolpin, D.W., Barnes, K.K., Furlong E.T., Meyer, M.T., Zaugg, S.D., Barber, L.B., Thurman, M.E., 2008. A national reconnaissance for pharmaceuticals and other organic wastewater contaminants in the United States - II) Untreated drinking water sources. Sci. Total Environ. 402 (2-3), 201-216.

Gagliano, E., Sgroi, M., Falciglia, P.P., Vagliasindi, F.G.A., Roccaro, P., 2020. Removal of poly- and perfluoroalkyl substances (PFAS) from water by adsorption: Role of PFAS chain length, effect of organic matter and challenges in adsorbent regeneration. Water Res. 171,115381.

Gao, P., Ding, Y., Li, H., Xagoraraki, I., 2012. Occurrence of pharmaceuticals in a municipal wastewater treatment plant: Mass balance and removal processes. Chemosphere 88 (1), 17-24.

Gerrity, D., Gamage, S., Holady, J.C., Mawhinney, D.B., Quiñones, O., Trenholm, R.A., Snyder, S.A., 2011. Pilot-scale evaluation of ozone and biological activated carbon for trace organic contaminant mitigation and disinfection. Water Res. 45 (5), 2155-2165.

Glassmeyer, S.T., Furlong, E.T., Kolpin, D.W., Cahill, J.D., Zaugg, S.D., Werner, S.L., Meyer, M.T., Kryak, D.D., 2005. Transport of chemical and microbial compounds from known wastewater discharges potential for use as indicators of human fecal contamination. Environ. Sci. Technol. 39 (14), 5157-5169.

Goodrow, S.M., Ruppel, B., Lippincott, R.L., Post, G.B., Procopio, N.A., 2020. Investigation of levels of perfluoroalkyl substances in surface water, sediment and fish tissue in New Jersey, USA. Sci. Total Environ. 729, 138839.

Gros, M., Petrović, M., Barceló, D., 2006. Development of a multi-residue analytical methodology based on liquid chromatography-tandem mass spectrometry (LC-MS/MS) for screening and trace level determination of pharmaceuticals in surface and wastewaters. Talanta 70 (4), 678-690.

Gross, B., Montgomery-Brown, J., Naumann, A., Reinhard, M., 2004. Occurrence and fate of pharmaceuticals and alkylphenol ethoxylate metabolites in an effluent-dominated river and wetland. Environ. Toxicol. Chem. 23 (9), 2074-2083.

Grube, A., Donaldson, D., Kiely, T., Wu, L. *Pesticides Industry Sales and Usage – 2006 and 2007 Market Estimates*; U.S. Environmental Protection Agency: Washington, DC, 2011.

Guo, Y.C., Krasner, S.W., 2009. Occurrence of primidone, carbamazepine, caffeine, and precursors for N-nitrosodimethylamine in drinking water sources impacted by wastewater. J. Am. Water Resour. Assoc. 45 (1), 58-67.

Hakk, H., Millner, P., Larsen, G., 2005. Decrease in water-soluble 17β-estradiol and testosterone in composted poultry manure with time. J. Environ. Qual. 34 (3), 943-950.

Hatem, A., Marton, S., Csóka, G., Rácz, I., 1996. Preformulation studies of atenolol in oral liquid dosage form. I. Effect of pH and temperature. Acta Pharm. Hung. 66 (4), 177-180.

Hazardous Substances Data Bank (HSDB), United States National Library of Medicine, http://toxnet.nlm.nih.gov/cgi-bin/sis/htmlgen?HSDB (accessed March 30, 2018).

Hollender, J., Zimmermann, B.G., Koepke, S., Krauss, M., Mcardell, C.S., Ort, C., Singer, H., Von Gunten, U., Siegrist, H., 2009. Elimination of organic micropollutants in a municipal wastewater treatment plant upgraded with a full-scale post-ozonation followed by sand filtration. Environ. Sci. Technol. 43 (20), 7862-7869.

Huber, M.M., Canonica, S., Park, G.-U., Von Gunten, U., 2003. Oxidation of pharmaceuticals during ozonation and advanced oxidation processes. Environ. Sci. Technol. 37 (5), 1016-1024.

Huber, M.M., Göbel, A., Joss, A., Hermann, N., Löffler, D., McArdell, C.S., Ried, A., Siegrist H., Ternes, T.A., Von Gunten, U., 2005. Oxidation of pharmaceuticals during ozonation of municipal wastewater effluents: A pilot plant. Environ. Sci. Technol. 39 (11), 4290-4299.

Huggett, D.B., Khan, I.A., Foran, C.M., Schlenk, D., 2003. Determination of beta-adrenergic receptor blocking pharmaceuticals in United States wastewater effluent. Environ. Pollut. 121 (2), 199-205.

Human Metabolome Database, HMDB version 4.0, http://www.hmdb.ca/ accessed March 30, 2018).

IMS Institute for Healthcare Informatics. *The Use of Medicines in the United States: Review of 2011*; Parsippany, NJ, 2012.

Javier Rivas, F., Sagasti, J., Encinas, A., Gimeno, O., 2010. Contaminants abatement by ozone in secondary effluents. Evaluation of second-order rate constants. J. Chem. Technol. Biotechnol. 86 (8), 1058-1066.

Karnjanapiboonwong, A., Morse, A.N., Maul, J.D., Anderson. T.A., 2010. Sorption of estrogens, triclosan, and caffeine in a sandy loam and a silt loam soil. J. Soils Sediments 10 (7), 1300-1307.

Kiely T., Donaldson D., Grube, A. *Pesticides Industry Sales andUusage – 2006 and 2007 Market Estimates*; U.S. Environmental Protection Agency: Washington, DC, 2004.

Kim, S.-C., Carlson, K., 2006. Occurrence of ionophore antibiotics in water and sediments of a mixed-landscape watershed. Water Res. 40 (13), 2549-2560.

Kolpin, D. W., Furlong, E. T., Meyer, M. T., Thurman, E. M., Zaugg, S. D., Barber, L. B., Buxton, H. T., 2002. Pharmaceuticals, Hormones, and Other Organic Wastewater Contaminants in U.S. Streams, 1999 - 2000: A National Reconnaissance. Environ. Sci. Technol. 36 (6), 1202-1211.

Konwick, B.J., Tomy, G.T., Ismail, N., Peterson, J.T., Fauver, R.J., Higginbotham, D., Fisk, A.T., 2008. Concentrations and patterns of perfluoroalkyl acids in Georgia, USA surface waters near and distant to a major use source. Environ. Toxicol. Chem. 27 (10), 2011-2018.

Kostich, M.S., Batt, A.L., Lazorchak, J.M., 2014. Concentrations of prioritized pharmaceuticals in effluents from 50 large wastewater treatment plants in the US and implications for risk estimation. Environ. Pollut. 184, 354-359.

Kwon, J.-W., Armbrust, K.L. (2008) Aqueous solubility, n-octanol-water partition coefficient, and sorption of five selective serotonin reuptake inhibitors to sediments and soils. Bull. Environ. Contam. Toxicol. 81 (2), 128-135.

Kwon, J.-W., Rodriguez, J.M., 2014. Occurrence and removal of selected pharmaceuticals and personal care products in three wastewater-treatment plants. Arch. Environ. Contam. Toxicol. 66 (4), 538-548.

Lara-Martín, P.A., González-Mazo, E., Petrovic, M., Barceló, D., Brownawell, B.J., 2014. Occurrence, distribution and partitioning of nonionic surfactants and pharmaceuticals in the urbanized Long Island Sound Estuary (NY). Mar. Pollut. Bull. 85 (2), 710-719.

Lavén, M., Alsberg, T., Yu, Y., Adolfsson-Erici, M., Sun, H., 2009. Serial mixed-mode cation- and anion-exchange solid-phase extraction for separation of basic, neutral and acidic

pharmaceuticals in wastewater and analysis by high-performance liquid chromatography-quadrupole time-of-flight mass spectrometry. J. Chromatogr. A 1216 (1), 49-62.

Lienert, Judit., Guedel, Karin., Escher, Beate I., 2007. Screening Method for Ecotosicological Hazard Assessment of 42 Pharmaceuticals Considering Human Metabolism and Excretory Routes. Environ. Sci. Technol. 41 (12), 4471-4478.

Loganathan, B.G., Sajwan, K.S., Sinclair, E., Senthil Kumar, K., Kannan, K., 2007. Perfluoroalkyl sulfonates and perfluorocarboxylates in two wastewater treatment facilities in Kentucky and Georgia. Water Res. 41 (20), 4611-4620.

Loraine, G.A., Pettigrove, M.E., 2006. Seasonal variations in concentrations of pharmaceuticals and personal care products in drinking water and reclaimed wastewater in Southern California. Environ. Sci. Technol. 40 (3), 687-695.

Luo, Y., Guo, W., Ngo, H.H., Nghiem, L.D., Hai, F.I., Zhang, J., Liang, S., Wang, X.C., 2014. A review on the occurrence of micropollutants in the aquatic environment and their fate and removal during wastewater treatment. Sci. Total Environ. 473-474, 619-641.

Maruya, K.A., Dodder, N.G., Sengupta, A., Smith, D.J., Lyons, J.M., Heil, A.T., Drewes, J.E., 2016. Multimedia screening of contaminants of emerging concern (CECs) in coastal urban watersheds in southern California (USA). Environ. Toxicol. Chem. 35 (8), 1986-1994.

Nanaboina, V., Korshin, G.V., 2010. Evolution of absorbance spectra of ozonated wastewater and its relationship with the degradation of trace-level organic species. Environ. Sci. Technol. 44 (16), 6130-6137.

Nghiem, L.D., Manis, A., Soldenhoff, K., Schafer, A.I., 2004. Estrogenic hormone removal from wastewater using NF/RO membranes. J. Membr. Sci. 242 (1-2), 37-45.

Nkedi-Kizza, P., Shinde, D., Savabi, M.R., Ouyang, Y., Nieves, L., 2006. Sorption Kinetics and Equilibria of Organic Pesticides in Carbonatic Soils from South Florida. J. Environ. Qual. 35 (1), 268-276.

Oppenheimer, J., Eaton, A., Badruzzaman, M., Haghani, A.W., Jacangelo, J.G., 2011. Occurrence and suitability of sucralose as an indicator compound of wastewater loading to surface waters in urbanized regions. Water Res. 45 (13), 4019-4027.

Padhye, L.P., Yao, H., Kung'u, F.T., Huang, C.-H., 2014. Year-long evaluation on the occurrence and fate ofpharmaceuticals, personal care products, andendocrine disrupting chemicals in an urban drinking water treatment plant. Water Res. 51, 266-276.

Post, G.B., Louis, J.B., Lippincott, R.L., Procopio, N.A., 2013. Occurrence of perfluorinated compounds in raw water from New Jersey public drinking water systems. Environ. Sci. Technol. 47 (23), 13266-13275.

Procopio, N.A., Karl, R., Goodrow, S.M., Maggio, J., Louis, J.B., Atherholt, T.B., 2017. Occurrence and source identification of perfluoroalkyl acids (PFAAs) in the Metedeconk River Watershed, New Jersey. Environ. Sci. Pollut. Res. 24 (35), 27125-27135.

Quiñones, O., Snyder, S.A., 2009. Occurrence of perfluoroalkyl carboxylates and sulfonates in drinking water utilities and related waters from the United States. Environ. Sci. Technol. 43 (24), 9089-9095.

Rahman, M.F., Peldszus, S., Anderson, W.B., 2014. Behaviour and fate of perfluoroalkyl and polyfluoroalkyl substances (PFASs) in drinking water treatment: A review. Water Res. 50, 318-340.

Reungoat, J., Macova, M., Escher, B.I., Carswell, S., Mueller, J.F., Keller, J., 2010. Removal of micropollutants and reduction of biological activity in a full scale reclamation plant using ozonation and activated carbon filtration. Water Res. 44 (2), 625-637.

Scheytt, T., Mersmann, P., Lindstädt, R., Heberer, T., 2005. Determination of sorption coefficients of pharmaceutically active substances carbamazepine, diclofenac, and ibuprofen, in sandy sediments. Chemosphere 60 (2), 245-253.

Schwab, B.W., Hayes, E.P., Fiori, J.M., Mastrocco, F.J., Roden, N.J., Cragin, D., Meyerhoff, R.D., D'Aco, V.J., Anderson, P.D., 2005. Human pharmaceuticals in US surface waters: A human health risk assessment. Regul. Toxicol. Pharm. 42 (3), 296-312.

Sengupta, A., Lyons, J.M., Smith, D.J., Drewes, J.E., Snyder, S.A., Heil, A., Maruya, K.A., 2014. The occurrence and fate of chemicals of emerging concern in coastal urban rivers receiving discharge of treated municipal wastewater effluent. Environ. Toxicol. Chem. 33 (2), 350-358.

Sinclair, E., Mayack, D.T., Roblee, K., Yamashita, N., Kannan, K., 2006. Occurrence of perfluoroalkyl surfactants in water, fish, and birds from New York State. Arch. Environ. Contam. Toxicol. 50 (3), 398-410.

Singh, S.P., Azua, A., Chaudhary, A., Khan, S., Willett, K.L., Gardinali, P.R., 2010. Occurrence and distribution of steroids, hormones and selected pharmaceuticals in South Florida coastal environments. Ecotoxicology 19 (2), 338-350.

Snyder, S.A., 2008. Occurrence, treatment, and toxicological relevance of EDCs and pharmaceuticals in water. Ozone: Sci. Eng. 30 (1), 65-69.

Snyder, S.A., Wert, E.C., Lei, H., Westerhoff, P., Yoon, Y. *Removal of EDCs and Pharmaceuticals in Drinking and Reuse Treatment Processes*; American Water Works Association Research Foundation: Denver, CO, 2007.

Spongberg, A.L., Witter, J.D., 2008. Pharmaceutical compounds in the wastewater process stream in Northwest Ohio. Sci. Total Environ. 397 (1-3), 148-157.

Stackelberg, P.E., Furlong, E.T., Meyer, M.T., Zaugg, B.D., Henderson, A.K., Reissman, D.B., 2004. Persistence of pharmaceutical compounds and other organic wastewater contaminants in a conventional drinking-water-treatment plant. Sci. Total Environ. 329 (1-3), 99-113.

Subedi, B., Kannan, K., 2015. Occurrence and fate of select psychoactive pharmaceuticals and antihypertensives in two wastewater treatment plants in New York State, USA. Sci. Total Environ. 514, 273-280.

Sun, M., Arevalo, E., Strynar, M., Lindstrom, A., Richardson, M., Kearns, B., Pickett, A., Smith, C., Knappe, D.R.U., 2016. Legacy and Emerging Perfluoroalkyl Substances Are Important Drinking Water Contaminants in the Cape Fear River Watershed of North Carolina. Environ. Sci. Technol. Lett. 3 (12), 415-419.

Tabe, S., Jamal, T., Seth, R., Yue, C., Yang, P., Zhao, X., Schweitzer, L. *ECs and EDC – Occurrence in the Detroit River and Their Removal by Ozonation*; Water Research Foundation: Denver, CO, 2009.

ter Laak, T.L., van der Aa, M., Houtman, C.J., Stoks, P.G., van Wezel, A.P., 2010. Relating environmental concentrations of pharmaceuticals to consumption: A mass balance approach for the river Rhine. Environ. Int. 36 (5), 403-409.

Tixier, C., Singer, H.P., Oellers, S., Müller, S.R., 2003. Occurrence and fate of carbamazepine, clofibric acid, diclofenac, ibuprofen, ketoprofen, and naproxen in surface waters. Environ. Sci. Technol. 37 (6), 1061-1068.

Trenholm, R.A., Vanderford, B.J., Holady, J.C., Rexing, D.J., Snyder, S.A., 2006. Broad range analysis of endocrine disruptors and pharmaceuticals using gas chromatography and liquid chromatography tandem mass spectrometry. Chemosphere 65 (11), 1990-1998

Trojanowicz, M., Bojanowska-Czajka, A., Bartosiewicz, I., Kulisa, K., 2018. Advanced Oxidation/Reduction Processes treatment for aqueous perfluorooctanoate (PFOA) and perfluorooctanesulfonate (PFOS) – A review of recent advances. Chem. Eng. J. 336, 170-199.

Vanderford, B.J., Snyder, S.A., 2006. Analysis of Pharmaceuticals in Water by Isotope Dilution Liquid Chromatography/Tandem Mass Spectrometry. Environ. Sci. Technol. 40 (23), 7312-7320.

Vedagiri, U.K., Anderson, R.H., Loso, H.M., Schwach, C.M., 2018. Ambient levels of PFOS and PFOA in multiple environmental media. Remediation 28 (2), 9-51.

Watts, M.J., Linden, K.G., 2009. Advanced oxidation kinetics of aqueous trialkyl phosphate flame retardants and plasticizers. Environ. Sci. Technol. 43(8), 2937-2942.

Westerhoff, P., Yoon, Y., Snyder, S., Wert, E., 2005. Fate of endocrine-disruptor, pharmaceutical, and personal care product chemicals during simulated drinking water treatment processes. Environ. Sci. Technol. 39 (17), 6649-6663.

Writer, J.H., Ferrer, I., Barber, L.B., Thurman, E.M., 2013. Widespread occurrence of neuro-active pharmaceuticals and metabolites in 24 Minnesota rivers and wastewaters. Sci. Total Environ. 461-462, 519-527.

Yamamoto, H., Hayashi, A., Nakamura, Y., Sekizawa, J., 2005. Fate and partitioning of selected pharmaceuticals in aquatic environment. Environ. Sci. 12 (6), 347-358.

Yamamoto, H., Nakamura, Y., Moriguchi, S., Nakamura, Y., Honda, Y., Tamura, I., Hirata, Y., Hayashi, A., Sekizawa, J., 2009. Persistence and partitioning of eight selected pharmaceuticals in the aquatic environment: Laboratory photolysis, biodegradation, and sorption experiments. Water Res. 43 (2), 351-362.

Yang, X., Flowers, R.C., Weinberg, H.S., Singer, P.C., 2011. Occurrence and removal of pharmaceuticals and personal care products (PPCPs) in an advanced wastewater reclamation plant. Water Res. 45 (16), 5218-5228.

Yargeau, V., Leclair, C., 2008. Impact of operating conditions on decomposition of antibiotics during ozonation: A review. Ozone: Sci. Eng. 30 (3), 175-188.

Yoon, Y., Westerhoff, P., Snyder, S.A., Wert, E.C., Yoon, J., 2007. Removal of endocrine disrupting compounds and pharmaceuticals by nanofiltration and pharmaceuticals. Desalination 202 (1-3), 16-23.

Yu, J.T., Bouwer, E.J., Coelhan, M., 2006. Occurrence and biodegradability studies of selected pharmaceuticals and personal care products in sewage effluent. Agric. Water Manage. 86 (1-2), 72-80.

Yu, Y., Wu, L., Chang, A.C., 2013. Seasonal variation of endocrine disrupting compounds, pharmaceuticals and personal care products in wastewater treatment plants. Sci. Total Environ. 442, 310-316.

Zhang, X., Lohmann, R., Dassuncao, C., Hu, X.C., Weber, A.K., Vecitis, C.D., Sunderland, E.M., 2016. Source Attribution of Poly- and Perfluoroalkyl Substances (PFASs) in Surface Waters from Rhode Island and the New York Metropolitan Area. Environ. Sci. Technol. Lett. 3 (9), 316-321.
